# A genome-wide association scan implicates *DCHS2*, *RUNX2*, *GLI3*, *PAX1* and *EDAR* in human facial variation

**DOI:** 10.1038/ncomms11616

**Published:** 2016-05-19

**Authors:** Kaustubh Adhikari, Macarena Fuentes-Guajardo, Mirsha Quinto-Sánchez, Javier Mendoza-Revilla, Juan Camilo Chacón-Duque, Victor Acuña-Alonzo, Claudia Jaramillo, William Arias, Rodrigo Barquera Lozano, Gastón Macín Pérez, Jorge Gómez-Valdés, Hugo Villamil-Ramírez, Tábita Hunemeier, Virginia Ramallo, Caio C. Silva de Cerqueira, Malena Hurtado, Valeria Villegas, Vanessa Granja, Carla Gallo, Giovanni Poletti, Lavinia Schuler-Faccini, Francisco M. Salzano, Maria- Cátira Bortolini, Samuel Canizales-Quinteros, Michael Cheeseman, Javier Rosique, Gabriel Bedoya, Francisco Rothhammer, Denis Headon, Rolando González-José, David Balding, Andrés Ruiz-Linares

**Affiliations:** 1 Department of Genetics, Evolution and Environment, UCL Genetics Institute, University College London, London WC1E 6BT, UK; 2 Departamento de Tecnología Médica, Facultad de Ciencias de la Salud, Universidad de Tarapacá, Arica 1000009, Chile; 3 Centro Nacional Patagónico, CONICET, Unidad de Diversidad, Sistematica y Evolucion, Puerto Madryn U912OACD, Argentina; 4 Laboratorios de Investigación y Desarrollo, Facultad de Ciencias y Filosofía, Universidad Peruana Cayetano Heredia, Lima 31, Perú; 5 Laboratorio de Genética Molecular, Escuela Nacional de Antropologia e Historia, México City 14030, México; 6 GENMOL (Genética Molecular), Universidad de Antioquia, Medellín 5001000, Colombia; 7 Unidad de Genómica de Poblaciones Aplicada a la Salud, Facultad de Química, UNAM-Instituto Nacional de Medicina Genómica, México City 4510, México; 8 Departamento de Anatomía, Facultad de Medicina, Universidad Nacional Autónoma de México (UNAM), México City 04510, México; 9 Departamento de Genética, Universidade Federal do Rio Grande do Sul, Porto Alegre 91501-970, Brasil; 10 Division of Developmental Biology, The Roslin Institute and Royal (Dick) School of Veterinary Studies, University of Edinburgh, Midlothian EH25 9RG, UK; 11 Departamento de Antropología, Universidad de Antioquia, Medellín 5001000, Colombia; 12 Instituto de Alta Investigación, Universidad de Tarapacá, Arica 1000000, Chile; 13 Schools of BioSciences and Mathematics and Statistics, University of Melbourne, Melbourne, Victoria 3010, Australia

## Abstract

We report a genome-wide association scan for facial features in ∼6,000 Latin Americans. We evaluated 14 traits on an ordinal scale and found significant association (*P* values<5 × 10^−8^) at single-nucleotide polymorphisms (SNPs) in four genomic regions for three nose-related traits: columella inclination (4q31), nose bridge breadth (6p21) and nose wing breadth (7p13 and 20p11). In a subsample of ∼3,000 individuals we obtained quantitative traits related to 9 of the ordinal phenotypes and, also, a measure of nasion position. Quantitative analyses confirmed the ordinal-based associations, identified SNPs in 2q12 associated to chin protrusion, and replicated the reported association of nasion position with SNPs in *PAX3*. Strongest association in 2q12, 4q31, 6p21 and 7p13 was observed for SNPs in the *EDAR*, *DCHS2*, *RUNX2* and *GLI3* genes, respectively. Associated SNPs in 20p11 extend to *PAX1*. Consistent with the effect of *EDAR* on chin protrusion, we documented alterations of mandible length in mice with modified Edar funtion.

## Abstract (web summary)

Humans show great diversity in facial appearance and this variation is highly heritable. Here, Andres Ruiz-Linares and colleagues examined facial features in admixed Latin Americans and identify genome-wide associations for 14 facial traits, including four gene loci (*RUNX2*, *GLI3*, *DCHS2* and *PAX1*) influencing nose morphology.

## Graphical abstract

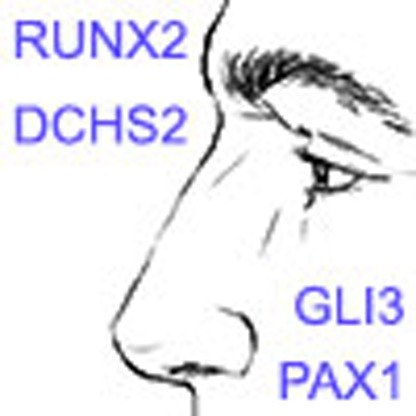

Humans show extensive variation in facial features, physical anthropologists having long used this variation to examine human population diversification, including the possibility that these features have been influenced by adaptation to the environment^1^,^2^,^3^. It has also been proposed that the diversity of human faces could have evolved partly to facilitate individual recognition, a key aspect of social interaction^4^. Other than their considerable evolutionary interest, a range of categorical and quantitative craniofacial traits have been used in forensics for the purpose of human identification and estimation of ancestry^5^,^6^. Consistent with facial variation being under strong genetic control, heritabilities of ∼60–90% have been estimated for facial phenotypes^7^,^8^,^9^. The characterization of gene mutations in patients with dysmorphologies and in animal models has enabled the identification of rare genetic variants with major effects on facial development^10^. However, our current understanding of the molecular genetic basis of variable face appearance in the general human population is scant. Only two genome-wide association studies (GWAS) for facial features have so far been published^9^,^11^. These were carried out in Europeans and only one gene region (*PAX3*) was consistently associated with a facial feature in both studies (position of the nasion, the deepest point on the nasal bridge)^9^,^11^.

We recently reported the successful identification of genes influencing outer ear (pinna) morphology using a three-point ordinal phenotyping approach^12^. Here, we extend this methodology to other aspects of facial variation. In addition, in a subsample of individuals, we obtained quantitative measures related to the ordinal phenotypes examined. Our analyses allowed us to replicate the reported *PAX3*/nasion position association and to identify five other gene regions impacting on face (mostly nose) morphology (*EDAR, DCHS2, RUNX2, GLI3* and *PAX1*). These genes are known to play important roles in craniofacial development.

## Results

### Study sample and ordinal phenotypes

**Figure 1:**
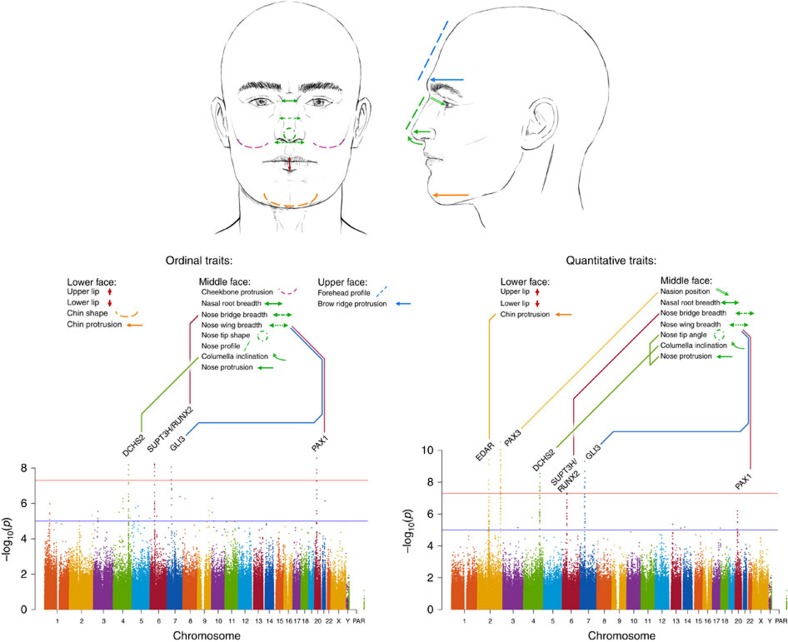
Overview of GWAS for facial features in the CANDELA sample. We first carried out a GWAS using data for 14 ordinal facial features from the lower, middle and upper face in 5,958 individuals. For follow-up, we obtained quantitative proxies for 9 of the 14 ordinal traits initially examined (and also obtained a measure of nasion position) in a subset of 2,955 individuals, and performed another GWAS. For convenience, we summarize results across traits on a single ‘composite' Manhattan plot shown at the bottom of the figure (ordinal traits on the left and quantitative traits on the right). Each Manhattan plot displays all the SNPs with *P* values exceeding thresholds for genome-wide suggestive (10^−5^, blue line) or genome-wide significance (5 × 10^−8^, red line) for any trait. To avoid cluttering the figure, *P* values not reaching the suggestive threshold (that is, whose significance can be disregarded) are shown only for one trait (upper lip thickness). The names of the candidate genes closest to each association peak are provided (Table 1). These genes are connected with the list of associated facial features via lines of different colour. The location of these features is illustrated on the face drawings shown at the top of the figure. Face drawings were prepared by Emiliano Bellini. PAR, pseudo-autosomal region.

Our study sample is part of the CANDELA cohort collected in Latin America^13^ (Supplementary Table 1). Using facial photographs of 6,275 individuals, we assessed 14 facial features on an ordered categorical scale reflecting the distinctiveness of each trait (Fig. 1, Supplementary Table 2). We included features of the lower face: chin shape, chin protrusion and upper/lower lip thickness; the middle face: cheekbone protrusion, breadth of nasal root, bridge and wing, columella inclination, nose protrusion, nose profile and nose tip shape; and the upper face: brow-ridge protrusion and forehead profile. These features were selected based on their documented variation in Europeans^5^. We found them to be reliably scored (Supplementary Table 3) and to also show extensive variation in the CANDELA sample (Supplementary Fig. 1). Individuals were genotyped on Illumina's OmniExpress BeadChip and imputation performed using 1000 Genomes data. After quality-control filters, final analyses were carried out on 671,038 genotyped single-nucleotide polymorphisms (SNPs) and 9,117,642 imputed SNPs in 5,958 individuals. On the basis of the genome-wide SNP data, average autosomal admixture proportions for the full sample were estimated as: 50% European, 45% Native American and 5% African (Supplementary Fig. 2).

Significant correlations were observed between the ordinal phenotypes (using a Bonferroni-adjusted permutation *P* value threshold for significance of 6 **×** 10^−4^, Supplementary Table 4A). Strongest correlation was observed between upper and lower lip thickness (*r*=0.72), followed by forehead profile and brow ridge protrusion (*r*=0.57). The three traits related to nose width (root, bridge and wing breadth) show positive correlations among them (*r*=0.16–0.37) and negative correlations with nose protrusion (*r*=−0.08 to −0.25). Several of the facial traits examined also show moderate (and significant) correlations with age, sex, body mass index (BMI) and genetic ancestry (Supplementary Table 4B). The strongest correlation with sex was seen for brow ridge protrusion and forehead profile (*r=*−0.62 and *r=*−0.47, respectively). Age correlates most strongly with upper and lower lip thickness (*r=*−0.19 and *r=*−0.24, respectively), while the strongest correlation for BMI was seen with brow-ridge protrusion (*r=*0.17). Genetic ancestry has strongest correlation with lip thickness (European ancestry being negatively correlated with upper and lower lip thickness, *r=*−0.25 and *r=*−0.16, respectively). European ancestry is also significantly correlated with all the nose features examined, particularly with nose protrusion (*r=*0.18) and nose wing breadth (*r=*−0.15). On the basis of a kinship matrix derived from the SNP data^14^, we estimated narrow-sense heritability for the facial traits using GCTA^15^. We found moderate (and significant) values for all traits, with the highest heritability being estimated for nose protrusion (0.47) and the lowest for columella inclination (0.20; Supplementary Table 5). Similar (or higher) heritabilities have been estimated for a range of facial traits using family data^7^,^8^,^16^.

### GWAS for ordinal phenotypes

**Figure 2:**
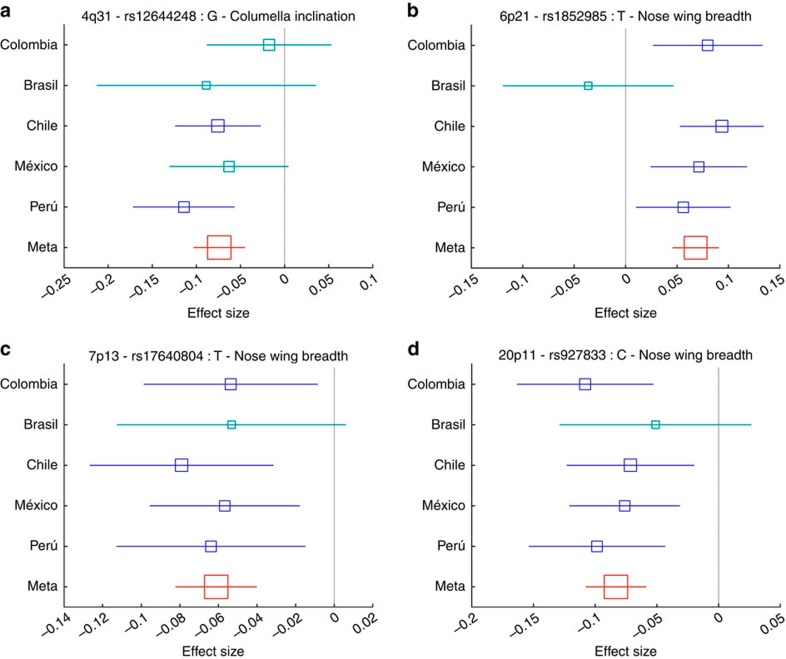
Effect sizes (regression coefficients) for the derived allele at index SNPs in the genome regions associated with ordinal face traits. (**a**) 4q31 rs12644248, (**b**) 6p21 rs1852985, (**c**) 7p13 rs17640804, (**d**) 20p11 rs927833. Estimates obtained in each country are shown as blue boxes. Red boxes indicate estimates obtained in the meta-analysis. Box size is proportional to sample size. Horizontal bars indicate confidence intervals representing 2 × standard errors. Intervals that include zero (that is, non-significant effects) are shown in light blue.

We performed genome-wide association tests using multivariate linear regression, as implemented in PLINK^17^, using an additive genetic model adjusting for: age, sex, BMI and the first five principal components (PCs, Supplementary Fig. 3) computed from the SNP data. The resulting statistics showed no evidence of residual population stratification for any of the traits (Supplementary Fig. 3). Three of the nose traits examined (columella inclination, nose bridge and wing breadth) showed genome-wide significant association (*P* values<5 × 10^−8^) with SNPs in at least one genomic region (Fig. 1, Table 1). Columella inclination and nose bridge breadth show association with SNPs in a single region (4q31 and 6p21, respectively), while nose wing breadth shows association with SNPs in two genomic regions (7p13 and 20p11). To account for the multiple phenotypes tested, we performed a global false-discovery rate test across all traits and SNPs and identified the same significantly associated regions (Supplementary Table 6). We examined association for each index SNP (the variant with the lowest *P* value in a chromosomal region; Table 1) in all countries sampled separately and combined results as a meta-analysis using METAL (Supplementary Table 7) (ref. 18). For all associations, significant effects were in the same direction in all countries, the variability of effect size across countries reflecting sample size (Fig. 2). There was no significant effect size heterogeneity across countries for any of the associations. To exploit the correlations observed between various facial traits, we performed a multivariate GWAS^19^, but this approach did not identify any additional associated regions (Supplementary Table 8).

### Follow-up analyses

Subsequent to the GWAS described above, we obtained data from an additional set of 501 individuals from the same countries as for the GWAS and used this as a replication sample (descriptive features of this sample are presented in Supplementary Fig. 4). These individuals were phenotyped and genotyped as for the GWAS sample. Association tests for the four index SNPs in Table 1 were performed using the same regression model as for the GWAS, with a Bonferroni-adjusted threshold for significance of 0.05/4=0.0125. All tests were found to be significant in this replication sample (Table 1).

We also followed-up the ordinal facial trait GWAS by obtaining facial measurements (distances and angles) related to the ordinal traits initially examined and performing a GWAS on these quantitative data. These measurements were obtained mainly using three-dimensional (3D) anatomical landmark coordinates available for 2,955 of the individuals included in the ordinal trait GWAS^20^ (Supplementary Fig. 5a). These landmarks allowed us to define quantitative proxies for seven of the ordinal facial traits, the other traits having no appropriate 3D landmarks allowing related measurements to be obtained (Supplementary Table 9). Since the ordinal assessment of nose root and bridge breadth produced genome-wide significant associations (but could not be measured with the 3D landmarks available), we carried out 2D landmarking of the frontal photographs of these 2,955 individuals and also obtained measurements for these two traits (Supplementary Table 10, Supplementary Fig. 5b). In addition, we used the 3D landmark coordinates to obtain a measure of nasion position so as to evaluate in our sample the reported association of this feature with SNPs in the *PAX3* gene region^9^,^11^.

The ordinal variables showed a moderate-to-high (and significant) correlation with the quantitative variables (all permutation *P* values<0.0005; Supplementary Table 11 and Supplementary Fig. 6). Correlation between ordinal and quantitative traits was strongest for nose wing breadth and lower lip thickness (both with *r*=0.70) and lowest for columella inclination (*r*=0.16). The pattern of correlation among quantitative traits was similar to that observed for the ordinal traits, as was the correlation between quantitative traits and covariates (Supplementary Table 12). As expected for continuous variables, heritability estimates based on the quantitative phenotypes (Supplementary Table 13) are higher than obtained for the ordinal phenotypes and more in line with published estimates^7^,^8^,^16^.

As before, we performed a GWAS for the quantitative traits using an additive multivariate regression model adjusting for age, sex, BMI and the first five PCs. We replicated the reported association of nasion position with SNPs in 2q35 overlapping the *PAX3* gene region, with strongest association seen for rs7559271 (*P* value of 4 × 10^−11^, Fig. 1, Table 2, Supplementary Fig. 7a). This is the same SNP producing strongest association in the Paternoster *et al*.^11^ GWAS. In addition, we observed genome-wide significant association for six of the nine quantitative proxies of the ordinal traits initially examined (Fig. 1, Table 2). As for the ordinal assessments, the quantitative analysis of columella inclination, nose bridge breadth and nose wing breadth produced genome-wide significant associations with SNPs in 4q31, 6p21 and 7p13, respectively (Fig. 1, Tables 1 and 2). In addition, the 4q31 region also showed genome-wide significant association to two other measurements related to nose morphology: nose protrusion and nose tip angle, with strongest *P* values for SNPs rs2045323 of 1 × 10^−8^ and 2 × 10^−8^, respectively. SNPs in 4q31 produced small but not genome-wide significant *P* values in the ordinal assessment of nose protrusion and nose tip angle (strongest *P* values of 4 × 10^−4^ and 3 × 10^−4^, respectively). The 20p11 region, showing genome-wide significant association in the ordinal assessment of nose wing breadth, showed genome-wide suggestive association in the quantitative trait GWAS (strongest *P* value of 6 **×** 10^−7^ for SNP rs927833). Other than reproducing the associations detected with ordinal traits, the quantitative analyses detected a genome-wide significant association to chin protrusion for markers in 2q12 (strongest *P* value of 4 × 10^−10^, for rs3827760; Fig. 1 and Table 2). This marker had an association *P* value of 1 **×** 10^−4^ in the ordinal assessment of chin protrusion.

A regression model similar to the one used in the GWAS analyses explains up to ∼30% of the phenotypic variation for the traits with significant SNP associations, with each of the associated SNPs explaining about 1% of variation in the trait (Tables 1 and 2, Supplementary Table 14). The estimates of trait variance explained by associated SNPs are similar to those calculated for other anthropometric traits and are very close to the estimates obtained in a previous GWAS for facial features^11^.

To assess independent evidence of association for the regions implicated here, we examined SNPs that produced at least genome-wide suggestive *P* values in the two GWAS for facial features that have been published^9^,^11^. We found that SNP rs2108166, 5.5 kb from and in high LD (*r*^2^=0.77, D′=1) with the index SNP of the 7p13 region we found associated with nose wing breadth (rs17640804), produced an association *P* value of 5 **×** 10^−7^ with the same trait in the study of Liu *et al*.^9^ In addition, evidence of association between rs3827760 and chin shape has recently been reported in a candidate gene study of a Central Asian population^21^.

It has been suggested that gene regions associated with non-syndromic cleft lip and palate (NSCL/P) might impact on normal variation in facial morphology^9^,^22^. Although the regions reported to be associated with NSCL/P do not overlap with those identified here, we selected index SNPs in each NSCL/P region and tested for association of these SNPs with the facial traits that we examined (Supplementary Table 15). Few tests survived Bonferroni correction, mostly involving SNPs associated with quantitative nose-breadth traits (nose root, nose bridge and nose wing breadth; Supplementary Table 15A). A global one-sided Kolmogorov–Smirnoff test was significant both for ordinal and quantitative traits (*P* value ∼10^−3^; Supplementary Table 15B) and a polygenic risk score test combining all 15 index SNPs was significant for the nose-breadth traits (Supplementary Table 15C). A more precise evaluation of the impact of NSCL/P-associated variants on facial variation in the general population requires further investigation.

### Candidate genes in regions associated with facial morphology

**Figure 3:**
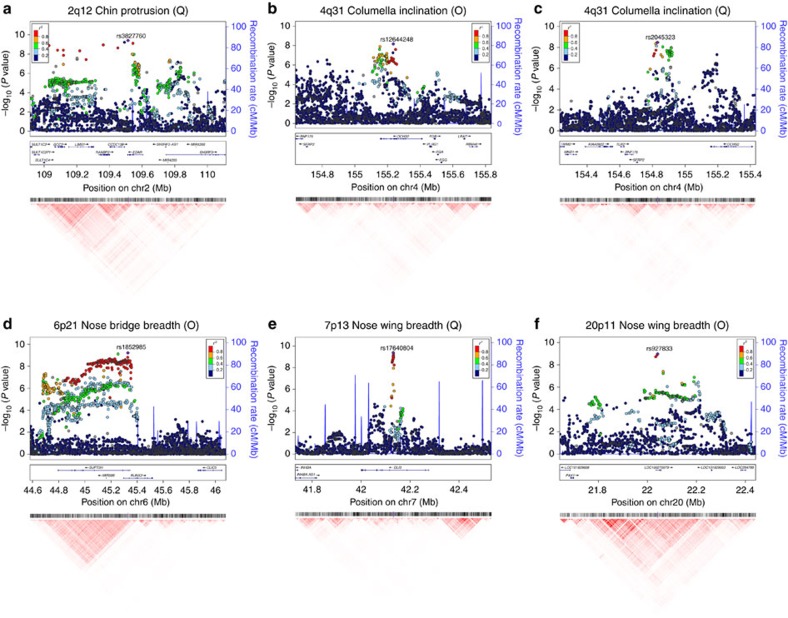
Genomic regions showing genome-wide significant association to face traits. For each facial feature we show the results that achieved strongest statistical significance regardless of the type of variable analysed (ordinal, O; or quantitative, Q). (**a**) 2q12 (Q), (**b**) 4q31 (O), (**c**) 4q31 (Q), (**d**) 6p21 (O), (**e**) 7p13(Q), (**f**) 20p11 (O). Plots not shown here are shown in Supplementary Fig. 7. Association results (on a −log_10_ P scale; left *y*-axis) are shown for SNPs ∼500 kb on either side of the index SNP (purple diamond; Table 1) with the marker (dot) colour indicating the strength of LD (*r*^2^) between the index SNP and that SNP in the 1000 genomes AMR data set. Local recombination rate in the AMR data is shown as a continuous blue line (scale on the right *y*-axis). Genes in each region, their intron–exon structure, direction of transcription and genomic coordinates (in Mb, using the NCBI human genome sequence, Build 37, as reference) are shown at the bottom. Plots were produced with LocusZoom^68^. Below each region we also show an LD heatmap (using *r*^2^, ranging from red indicating *r*^2^=1 to white indicating *r*^2^=0) produced using a MATLAB^59^ implementation similar to Haploview^69^.

SNPs in 2q12 associated with chin protrusion show extensive LD and overlap the 3′-half of the EctodysplasinA (EDA) receptor gene (*EDAR*; Fig. 3a). The derived G allele at the index SNP in this region (rs3827760) encodes a functional substitution in the intracellular death domain of EDAR (370A) and is associated with reduced chin protrusion (Table 2). EDAR is part of the EDA signalling pathway (comprising EDA, EDAR and EDARADD (the EDAR-binding death domain adaptor protein)) which specifies prenatally the location, size and shape of ectodermal appendages (such as hair follicles, teeth and glands)^23^. The death domain has been shown to be involved in the interaction of EDAR with EDARADD, the 370A form having higher activity than the ancestral variant^24^. The G allele at rs3827760 is not present in Europeans and Africans but is seen at high frequency in East Asians and is essentially fixed in Native Americans (Table 3). This SNP has been associated in East Asians with characteristic tooth morphologies, hair type and sweat gland density^25^,^26^,^27^. Recently, we showed, in the same study sample examined here, that rs3827760 impacts on aspects of pinna morphology, including: lobe size and attachment, ear protrusion and helix rolling^12^. Mutations in the EDA pathway cause hypohidrotic ectodermal dysplasia^28^. This disorder is characterized by a reduced number of sweat glands, oligodontia, decrease in the amount of hair and facial dysmorphia, including a markedly protrusive chin^29^.

**Figure 4:**
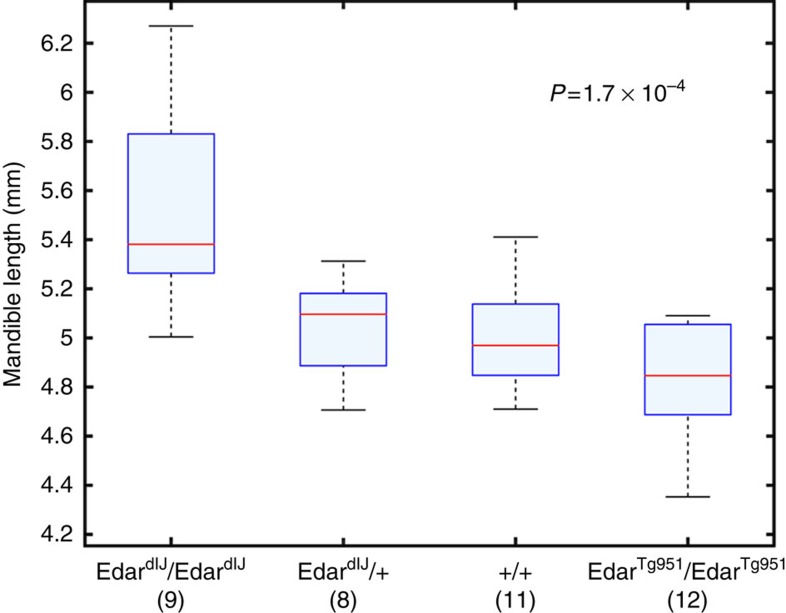
Effect of *Edar* genotype on mouse mandible length. We show boxplots of mandible length (*y*-axis) in mice with different *Edar* genotypes (*x*-axis). The measure of mandible length shown is the projected distance between head landmarks 5 and 10 (Supplementary Figs 8 and 9). Regression analysis indicates a significant effect of *Edar* genotype on mandible length (*P* value 1.7 × 10^−4^). Significant results were also obtained for other measurements of mandible length (Supplementary Table 16). Boxplot whiskers extend to data points within 1.5 times the interquartile range on both sides. The numbers in parenthesis below genotypic categories refer to the number of mice examined for each genotype.

Mouse *Edar* mutant and transgenic lines with either abolished or increased expression of *Edar* have been described and these mice show features related to several of the phenotypes associated with *EDAR* in humans^12^,^30^,^31^. Of particular interest, we recently documented that these mice show changes in ear morphology consistent with the effects of *EDAR* on human ear shape variation^12^. We therefore compared mandible length in *Edar* wild-type mice with *Edar*^*dlJ*^ and *Edar*^*Tg951*^ mutant mice (Supplementary Figs 8 and 9), which have a loss and a gain of *Edar* function, respectively^31^,^32^. We found a significant association of mandible length with genotype, with the length decreasing at greater *Edar* function, consistent with the association of the 370A variant with decreased chin protrusion detected in the CANDELA sample (Fig. 4, Supplementary Table 16). Consistent with the mandible length changes we detect in *Edar* mutant lines, it has been reported that *Eda* mouse mutants also show mandibular morphology alterations^33^. The impact of the *Eda* pathway on mandibular morphology has been interpreted as resulting from epithelial–mesenchymal interactions during mouse craniofacial development^33^.

SNPs in the 4q31 region with *P* values above the suggestive association threshold in the ordinal trait assessment of columella inclination extend over ∼400 kb from the 3′-half of the *Dachsous Cadherin-Related 2* gene (*DCHS2*) into the *DCHS2–SFRP2* (*Secreted Frizzled-related protein 2*) intergenic region (Fig. 3b), with strongest association seen for SNP rs12644248 within *DCHS2* (*P* value 7 × 10^−9^). Noticeably, although association analyses based on the quantitative assessment of columella inclination also show genome-wide significant association for rs12644248 (*P* value of 4 × 10^−8^), the quantitative analyses show that SNPs in the *DCHS2–SFRP2* intergenic region have an even stronger association, peaking at rs2045323 (*P* value of 3 × 10^−9^, Table 2, Fig. 3c). A similar pattern of association is seen for the quantitative assessments of nose protrusion and nose tip angle, with strongest association for both traits being observed for rs2045323 (*P* values of 1 × 10^−8^ and 2 × 10^−8^, respectively, Table 2, Supplementary Fig. 7), association with rs12644248 only exceeding the genome-wide suggestive threshold (*P* values of 8 × 10^−6^ and of 6 × 10^−6^ for nose protrusion and nose tip angle, respectively). SNP rs2045323 is not in strong LD with rs12644248 and tests conditioned on either SNP attenuate the signal of association at the other SNP but do not abolish it entirely (Supplementary Fig. 10). These observations suggest that the signal of association around rs2045323 in the *DCHS2–SFRP2* intergenic region is somewhat independent from that peaking at rs12644248 within *DCHS2*. Intergenic SNP rs2045323 is located in an evolutionarily conserved region (Supplementary Fig. 11), suggesting that this SNP could play a role in the regulation of genes in the region. *DCHS2* is a calcium-dependent cell-adhesion protein which has recently been shown to participate in a regulatory network controlling cartilage differentiation and polarity during vertebrate craniofacial development^34^. This network includes *SOX9*, a well-known regulator of cartilage differentiation, mutations of which lead in humans to Campomelic Dysplasia (OMIM #114290) a disorder characterized by a range of craniofacial defects. Although *DCHS2* seems the strongest candidate in the 4q31 region, *SFRP2* is also an interesting candidate, in that it has been shown that this gene is expressed in osteoblasts, participates in the regulation of Wnt signaling^35^ and craniofacial malformations have been reported in *Sfrp2* mutant mice^36^.

The 6p21.1 region associated with nose bridge breadth extends across ∼500 kb overlapping the suppressor of Ty 3 homologue (*S. cerevisiae*; *SUPT3H*) gene and the 5′-half of the *Runt-related transcription factor 2* (*RUNX2*) gene (Fig. 3d). Strongest association is seen for SNPs in the region of *SUPT3H/RUNX2* overlap, peaking at SNP rs1852985 for both the ordinal and the quantitative assessment of nose bridge breadth (Fig. 3d, Supplementary Fig. 7). This region is known to contain key *RUNX2* regulatory elements^37^ (Supplementary Fig. 12). Rare mutations in *RUNX2* cause Cleidocranial dysplasia, an autosomal dominant disorder involving alterations of cranial ossification (OMIM #119600). *Runx2* has been shown to participate in the differentiation of mouse osteoblasts, chondrocyte and mesenchymal stem cells and bone development^38^, null *Runx2* mutants showing a range of chondrocyte proliferation and maturation defects^39^. Interestingly, the length of a functional glutamine/alanine repeat in *RUNX2* has been shown to correlate strongly with the evolution of facial length in dog breeds and, more broadly, in Carnivora^40^.

SNPs in the 7p13 region associated with nose wing breadth extend over ∼80 kb within the third intron of the *GLI Family Zinc-Finger 3* gene (*GLI3*; Fig. 3e), a DNA-binding transcription factor. Strongest association for both the ordinal and quantitative assessments of nose wing breadth is observed for SNP rs17640804 (Tables 1 and 2, Fig. 3e, Supplementary Fig. 7), located in a genomic region with strong evolutionary conservation (Supplementary Fig. 13). Chromatin immunoprecipitation experiments have shown that rs17640804 can affect the binding of regulatory proteins^41^. *GLI3* is known to act both as activator and repressor in the sonic hedgehog signalling pathway, a key regulatory of chondrocyte differentiation^42^. Interestingly, it has been shown experimentally that *Gli3* interacts with *Runx2* in the regulation of mouse osteoblast differentiation^43^. We therefore tested for statistical interaction between the *GLI3* and *RUNX2* index SNPs on nose bridge breadth and found it to be significant (*P* value=0.004, Supplementary Table 17), even though the *GLI3* index SNP by itself does not have a significant effect on nose bridge breadth. Mutations in *GLI3* have been shown to cause several Mendelian disorders associated with craniofacial and limb abnormalities, including GCPS (Greig cephalopolysyndactyly syndrome). GCPS is characterized by a range of craniofacial abnormalities including a broad nose^44^. A mouse null *Gli3* mutant has been reported to show a range of craniofacial abnormalities, including a wider nose^45^.

Strongest association in 20p11 with the ordinal assessment of nose wing breadth was observed for SNP rs927833 located in LOC100270679, a long intergenic non-protein coding RNA (LINC01432). There is substantial LD around this SNP and suggestive evidence of association (that is, *P* values <10^−5^), for SNPs over a region of ∼400 kb extending to the *Paired-box gene 1* (*PAX1*; Fig. 3f), a strong candidate gene in this region. *PAX1* is a key developmental transcription factor which has been shown experimentally to affect chondrocyte differentiation through its participation in a regulatory pathway that also includes *RUNX2* and *SOX9* (ref. 46). More broadly, a *Pax*-*Six*-*Eya*-*Dach* (*Dachshund*) network, involving protein–protein and protein–DNA interactions impacting on a range of basic developmental processes has been described^47^. As indicated above, another *PAX* gene (*PAX3*) has been twice reported to impact on nasion position^9^,^11^, and we replicate that association here. A missense mutation in *PAX1* has been shown to cause autosomal recessive oto-facio-cervical syndrome, a disorder characterized by various skeletal and facial abnormalities^48^. It has also been reported that mouse embryos with *Gli3*-null mutations display drastically reduced *Pax1* expression, possibly mediated through *Gli3*'s involvement in the sonic hedgehog signalling pathway^49^. Consistent with these experimental findings, we observe a significant statistical interaction of the *GLI3* and *PAX1* index SNPs on nose wing breadth (*P* value=0.005, Supplementary Table 17).

## Discussion

Since quantitative traits are expected to provide higher power for detecting genetic effects than categorical traits, most recent efforts to identify genes for facial features have focused on quantitative assessments from 3D image data^9^,^11^,^50^,^51^. However, thus far the use of these phenotyping tools has not resulted in many robust genetic finds, mainly the *PAX3*-nasion position association replicated here^9^,^11^. Rather surprisingly, because of the comparatively lower power of non-quantitative phenotyping, we recently reported that using a simple ordinal phenotyping approach based on standard 2D photographs we were able to identify loci influencing pinna morphology^12^. Similar categorical rating scales have been used previously for the identification of genes for other anthropological features, such as pigmentation, hair type and tooth morphology^25^,^52^. Here, we confirm that categorical scales can be used to identify gene loci impacting on morphological features akin to those examined in certain anthropological and forensics settings^6^,^53^,^54^. We believe that our ability to detect genetic effects for such categorical traits arises from the high statistical power of the CANDELA sample for association testing of anthropological features, due to its comparatively large sample size, and particularly because of its extensive phenotypic and genetic diversity. This diversity relates to the admixed nature of this sample, admixture having involved continental populations with a relatively large genetic and phenotypic differentiation (mainly Europeans and Native Americans). This sample, thus, represents a sort of natural experiment facilitating the detection of genetic effects, especially for phenotypes differentiated between Europeans and Native Americans. Consistent with admixture having provided added power for association testing of the facial features examined, allele frequencies at the index SNPs in the novel face loci identified here show large differences between Europeans and East Asians/Native Americans and intermediate frequencies in the CANDELA sample (Table 3). Detection of genetic effects for these alleles would thus have relatively lower power in un-admixed continental populations. Consistent with this, independent evidence of association of chin shape with SNPs in the *EDAR* region has been recently reported in a Central Asian population with both Eastern and Western Eurasian ancestry^21^. Furthermore, power for the quantitative analyses performed here was likely increased by the preceding categorical analyses in that we focused on quantitative measures related to the ordinal traits, thus avoiding the considerable multiple-testing problem that can arise from the agnostic use of facial landmarks^51^.

Interestingly, we find no overlap between the gene regions affecting nose shape identified here and those we identified previously for pinna morphology in the same study sample^12^. Although, our current analyses certainly detect only some of the loci affecting these structures, our observations are consistent with the suggestion that facial features could be influenced by numerous genes with independent effects on different structures^22^. In fact, anatomical studies have placed the nose and the pinna in different developmental modules^55^. The possibility that variation in specific craniofacial structures could result from the action of different genes might also contribute to explain why quantitative analyses using whole-face shape summaries from 3D images have had limited success in detecting significant genetic effects^9^,^11^,^50^,^51^. If different genes act mainly on different facial structures (and on specific aspects of those structures) higher power to detect these genetic effects could be provided by more narrowly defined variables (for example, distances) than by broad-shape summaries (for example, PCs).

Four of the gene regions identified here (*DCHS2, RUNX2, GLI3* and *PAX1*) affect nose morphology. These results are consistent with the relatively high heritability of central middle face structures^8^ and the findings of the two published face-features GWAS, which also implicated mainly nose-related traits^9^,^11^. The shape of the human nose results from the coordinated development of mid-face bones and cartilages, including several in the nasal cavity^56^. Appropriately, the most compelling candidate genes in the regions we identified have well-established effects in cartilage and bone differentiation and have been shown to impact on craniofacial development in animal models. Interestingly, the analysis of genome sequences from modern and archaic humans (Neanderthals and Denisova) have identified *DCHS2*, *GLI3* and *RUNX2* among the top candidate genes harbouring highly differentiated variants and signatures of recent selection in the branches leading to these groups^57^,^58^. This observation has been interpreted as suggesting that these genes could be involved in the phenotypic differentiation of modern and archaic humans. Furthermore, using ancestry information and tests for accelerated evolution Claes *et al*.^50^ identified *GLI3* as a gene undergoing rapid evolution in modern humans. The effect of *EDAR* on chin protrusion adds to the developing picture of this gene having a multitude of phenotypic effects in populations with East Asian and Native American ancestry, the 370A allele having been associated so far with: increased sweat gland density^27^, straight hair^12^,^26^, lower beard and eye-brow thickness^52^, increased incisor shovelling^25^and a range of pinna features^12^.

In conclusion, we have identified five gene regions influencing normal variation in facial features. These regions harbour strong candidate genes, which independent evidence implicates in craniofacial development and evolution. It will be interesting to examine further the role that these gene regions might play in the evolutionary diversification of facial features in mammals, including the appearance of derived features in archaic and modern humans, as well as their potential involvement in the evolution of adaptive features of facial anatomy. The results presented here (and in related publications^12^,^52^) illustrate the high power provided by the CANDELA sample for the genetic analysis of phenotypes differentiated between Native Americans and Europeans. Further work on this sample, including additional quantitative trait analyses and the exploitation of 3D imaging techniques, should help delineate more fully the genetic architecture of the human face, including the possible overlap with gene regions implicated in common, complex alterations of facial development, such as NSCL/P.

## Methods

### Study subjects

In all, 6,275 volunteers from 5 countries (Colombia, *N*=1,402; Brasil, *N*=658; Chile, *N*=1,760; Mexico, *N*=1,200; and Peru, *N*=1,255), part of the CANDELA consortium sample (http://www.ucl.ac.uk/silva/candela)^13^, aged between 18 and 45 years were included in this study (Supplementary Table 1). Ethics approval was obtained from: Universidad Nacional Autónoma de México (México), Universidad de Antioquia (Colombia), Universidad Perúana Cayetano Heredia (Perú), Universidad de Tarapacá (Chile), Universidade Federal do Rio Grande do Sul (Brasil) and University College London (UK). All participants provided written informed consent. Individuals with dysmorphologies, a history of facial surgery or trauma, or with BMI over 33 were excluded (due to the effect of obesity on facial features). Blood samples were collected by a certified phlebotomist and DNA extracted following standard laboratory procedures. Subsequent to the GWAS, an additional 501 individuals were recruited to serve as a replication sample (Supplementary Fig. 4). These individuals were recruited following the same procedures as for the sample included in the GWAS.

### Ordinal phenotyping

This was carried out in the same way for the GWAS and replication samples. Right side and frontal photographs were used to score 14 facial traits. This included: chin shape and protrusion, cheekbone and brow-ridge protrusion, forehead profile, upper and lower lip thickness and seven nose features (breadth of nasal root, bridge and wing, columella inclination, nose protrusion, nose profile and nose tip shape). These features were selected based on their reported variation in European populations^5^. Software to assist scoring of photographs was developed in MATLAB (ref. 59). Intraclass correlation coefficients (ICCs)^60^ calculated by repeated scoring of photographs of 450 subjects by two independent raters (M.F.-G. and I.P.A.) indicate a moderate–to-high intra-rater reliability of the trait scores (Supplementary Table 3), with relatively lower inter-rater reliability for certain traits. Photographs for all the volunteers were scored by the same rater (M.F.-G.).

### Quantitative phenotyping

Quantitative phenotypes were obtained using Procrustes-adjusted 3D facial landmark coordinates available for 2,955 of the individuals included in the ordinal trait GWAS. These coordinates were obtained for 34 anatomical landmarks as detailed in ref. 20 (Supplementary Fig. 5). Briefly, landmarks were placed and raw 3D coordinates obtained using Photomodeler software and five facial photographs (taken at 0°, 45°, 90°, 135° and 180°, where 0° is the left side view). The raw 3D landmark coordinates were Procrustes-adjusted using the MorphoJ software^61^. Quantitative measurements (distances and angles) were defined corresponding to seven of the ordinal traits initially examined (Supplementary Table 9). Since no 3D landmarks allowing quantitative proxies for nose root and bridge breadth were available we placed 2D landmarks on the frontal photographs of the same individuals with 3D landmarks (Supplementary Fig. 5, Supplementary Table 10): two landmarks were added each for nasal root and for nose bridge width, in addition to the major frontally visible 3D landmarks. Since the 3D coordinates are free of head tilts and rotations (thus allowing more accurate measurements) the 2D coordinates were calibrated with reference to the 3D coordinates using corresponding frontal landmarks (having both 2D and 3D coordinates) (Supplementary Fig. 5a,b).

### DNA genotyping and quality control

DNA samples from participants were genotyped on the Illumina HumanOmniExpress chip including 730,525 SNPs. PLINK v1.9 (ref. 62) was used to exclude SNPs and individuals with >5% missing data, markers with minor-allele frequency <1%, related individuals (Plink IBD estimate>0.1), and those who failed the X-chromosome sex concordance check (sex estimated from X-chromosome heterozygosity not matching recorded sex information). After applying these filters 671,038 SNPs and 5,958 individuals (1,303 from Colombia, 608 from Brasil, 1,651 from Chile, 1,165 from Mexico, 1,231 from Peru) were retained for further analysis. Due to the admixed nature of the study sample (Supplementary Fig. 2) there is an inflation in Hardy–Weinberg *P* values. We therefore did not exclude markers based on Hardy–Weinberg deviation, but performed stringent quality controls at software and biological levels, and checked the genotyping cluster plots for each index SNP manually (Supplementary Fig. 14). The replication sample was genotyped in the same way and the genotype data submitted to the same quality controls as for the GWAS sample.

### SNP genotype imputation

The chip genotype data was phased using SHAPEIT2 (ref. 63). IMPUTE2 (ref. 64) was then used to impute genotypes at untyped SNPs using variant positions from the 1000 Genomes Phase I data. The 1000 Genomes reference data set includes haplotype information for 1,092 individuals across the world for 36,820,992 variant positions. Positions that are monomorphic in 1000 Genomes Latin American samples (CLM, MXL and PUR) were excluded, leading to 11,025,002 SNPs being imputed in our data set. Of these, 48,695 had imputation quality scores <0.4 and were excluded. Chip genotyped SNPs having a low concordance value (<0.7) or a large gap between info and concordance values (info_type0—concord_type0>0.1), which might be indicators of poor genotyping, were also removed, both from the imputed and chip data set. The IMPUTE2 genotype probabilities at each locus were converted into best-guess genotypes using PLINK^62^ (at the default setting of <0.1 uncertainty). SNPs with proportion of samples with uncalled genotypes>5% and minor-allele frequency<1% were excluded. The final imputed data set contained genotypes for 9,117,642 SNPs.

### Statistical genetic analyses

Narrow-sense heritability (defined as the additive phenotypic variance explained by a Genetic Relatedness Matrix, GRM, computed from the SNP data) was estimated using GCTA^15^ by fitting an additive linear model with a random-effect term whose variance is given by the GRM, with age, sex and BMI as covariates. The GRM was obtained using the LDAK approach^14^, which accounts for LD between SNPs. An LD-pruned set of 93,328 autosomal SNPs was used to estimate European, African and Native American ancestry using supervised runs of ADMIXTURE^65^ (Supplementary Fig. 2). Reference parental populations included in the ADMIXTURE analyses consisted of Africans and Europeans from HAPMAP and selected Native Americans, as described in Ruiz-Linares *et al*.^13^

PLINK 1.9 (ref. 62) was used to perform the primary genome-wide association tests for each phenotype using multiple linear regression with an additive genetic model incorporating age, sex, BMI and five genetic PCs as covariates. Association analyses were performed on the imputed data set with two approaches: using the best-guess imputed genotypes in PLINK and using the IMPUTE2 genotype probabilities in SNPTEST v2.5 (ref. 66). Both were consistent with each other and with the results from the chip genotype data. For analysis of the X chromosome an inactivation model was used (male genotypes encoded as 0/2 and female genotypes as 0/1/2). The genetic PCs were obtained (using PLINK 1.9 (ref. 62) from an LD-pruned dataset of 93,328 SNPs. They were selected by inspecting the proportion of variance explained and checking scree and PC scatter plots (Supplementary Fig. 3a). Individual outliers were removed and PCs recalculated after each removal. The top PCs appear to be a good proxy for continental ancestry (Supplementary Fig. 3b). Using these PCs the Q–Q plots (Supplementary Fig. 3c) for all association tests showed no sign of inflation, the genomic control factor lambda being<1.02 in all cases (Supplementary Fig. 3d), thus confirming that we are appropriately accounting for population stratification^67^. Similar analyses were applied for association testing of the index SNPs followed-up in the replication sample. To account for multiple testing we also applied a global false-discovery rate test using the Benjamini–Hochberg procedure across all traits and SNPs (Supplementary Table 6). To account for the correlations between traits, a multivariate GWAS was also performed, testing for association with all facial traits simultaneously using a Wald test conditioned on all covariates (Supplementary Table 8). A meta-analysis was carried out for the index SNPs identified in the primary analyses by testing for association separately in each country sample and combining the results (using the PLINK implementation of the meta-analysis software METAL^18^). Forest plots were produced with MATLAB. Cochran's *Q*-statistic was computed for each trait to test for effect-size heterogeneity across country samples. The fraction of trait variance explained by the covariates, by each index SNP, and by all index SNPs altogether, were estimated from linear regression models implemented using R^2^ (Supplementary Table 14). To evaluate the role of NSCL/P loci on the facial traits examined we selected index SNPs in the 15 associated regions reported in the literature (Supplementary Table 15) and performed individual SNP associations, global Kolmogorov–Smirnov tests and Polygenic Risk Score tests using PLINK.

### Mouse analyses

Animal studies were reviewed and approved by The Roslin Institute Animal Welfare and Ethical Review Body (AWERB). The humane care and use of mice (*Mus musculus*) in this study was carried out under the authority of the appropriate UK Home Office Project License. The mouse samples and head photographs examined are from the same set described fully in Adhikari *et al*.^12^ Briefly, we included fourteen and 15-day-old animals (17 males and 23 female). The mouse genotypes were *Edar*^*dlJ*^ (a loss of function EDARp.E379K mutation^32^) as either homozygote or heterozygote, wild-type (+/+) and the homozygous *Edar*^*Tg951*^ line (which has ∼16 extra copies of *Edar* per haploid genome^31^). Thirteen 2D anatomical landmarks were placed on lateral photographs of the mouse heads, using TPSDig and TPSUtil (http://life.bio.sunysb.edu/morph/; Supplementary Fig. 8). Generalized procrustes analysis was carried out using the software MorphoJ^61^ to check whether the distribution of landmarks was homogeneous. No outliers were detected. Mouse mandible length was measured using the landmark coordinates (as detailed in Supplementary Figs 8 and 9) and mandible length (as a proportion of head size, measured directly on the heads) was regressed onto age, sex and *Edar* genotype. In this regression *Edar* genotype was coded as 1–4 based on increasing *Edar* expression: 1- *Edar*^*dlJ/dlJ*^ homozygotes, 2-*Edar*^*dlJ/+*^ heterozygotes, 3-wild-type^*+/+*^mice and 4-*Edar*^*Tg951/ Tg951*^ homozygotes (Supplementary Table 16).

## Additional information

**Accession codes:** The MATLAB program used to perform the ordinal scoring of facial features can be downloaded from http://www.ucl.ac.uk/silva/candela. *P* values for all SNPs tested in the GWAS analyses will be hosted in GWAS Central (http://www.gwascentral.org/), and also made available through http://www.ucl.ac.uk/silva/candela on the next data release of the GWAS Central database, scheduled for June 2016.

**How to cite this article:** Adhikari, K. *et al*. A genome-wide association scan implicates *DCHS2*, *RUNX2*, *GLI3*, *PAX1* and *EDAR* in human facial variation. *Nat. Commun.* 7:11616 doi: 10.1038/ncomms11616 (2016).

## Supplementary Material

Supplementary Information Supplementary Figures 1-14, Supplementary Tables 1-17 and Supplementary References

## Figures and Tables

**Table 1:** Properties of index SNPs in chromosomal regions showing genome-wide significant association to ordinal facial traits.

**Chromosomal region**	**Index SNP**	**Associated trait**	***P-*****value**	Candidate gene*	**Alleles**†	**Effect size**	**Percentage of variance explained**	**Replication** ***P-*****value**
4q31	rs12644248	Columella inclination	7 × 10^−9^	***DCHS2***	A>G	−8.40 × 10^−2^	0.49	4 × 10^−3^
6p21	rs1852985	Nose bridge breadth	6 × 10^−10^	***SUPT3H/RUNX2***	C>T	6.90 × 10^−2^	0.71	5 × 10^−3^
7p13	rs17640804	Nose wing breadth	9 × 10^−9^	***GLI3***	C>T	−6.50 × 10^−2^	0.62	6 × 10^−3^
20p11	rs927833	Nose wing breadth	1 × 10^−9^	*PAX1*	T>C	−7.70 × 10^−2^	0.66	4 × 10^−3^

SNP, single-nucleotide polymorphism.

^*^For intragenic SNPs, gene names are shown in bold.

^†^Derived alleles are shown after ancestral alleles.

**Table 2:** Properties of index SNPs in regions showing genome-wide significant association to quantitative facial traits.

**Chromosomal region**	**Index SNP**	**Associated trait**	***P-*****value**	Candidate gene*	**Alleles**†	**Effect size**	**Percentage of variance explained**
2q12	rs3827760	Chin protrusion	4 **×** 10^−10^	***EDAR***	A>G	−7.60 **×** 10^−3^	1.32
2q35	rs7559271	Nasion position	4 **×** 10^−11^	***PAX3***	A>G	8.20 **×** 10^−2^	1.33
4q31	rs2045323	Columella inclination‡	3 **×** 10^−9^	*DCHS2*	G>A	1.80 **×** 10^−2^	0.63
4q31	rs2045323	Nose protrusion	1 **×** 10^−8^	*DCHS2*	G>A	−5.90 **×** 10^−4^	0.95
4q31	rs2045323	Nose tip angle	2 **×** 10^−8^	*DCHS2*	G>A	1.60 **×** 10^−2^	1.08
6p21	rs1852985	Nose bridge breadth	2 **×** 10^−8^	***SUPT3H/RUNX2***	C>T	4.40 **×** 10^−4^	1.18
7p13	rs17640804	Nose wing breadth	5 **×** 10^−10^	***GLI3***	C>T	−4.90 **×** 10^−4^	1.15

SNP, single-nucleotide polymorphism.

^*^For intragenic SNPs, gene names are shown in bold.

^†^Derived alleles are shown after ancestral alleles.

^‡^Columella inclination was measured as an angle which decreases at greater ordinal columella inclination (Supplementary Table 9, Supplementary Fig. 6f). Therefore, the allelic effects for the quantitative and ordinal assessments of this trait (Table 1) are of opposite sign. rs12644248, the index SNP associated with categorical columella inclination has a *P* value of 4 **×** 10^−8^ for association with the quantitative assessment of columella inclination.

**Table 3:** Population frequency of derived alleles at index SNPs associated with facial features in the CANDELA sample.

**Region**	**SNP**	**Allele**	Frequency (%)*				
			**CEU**	**YRI**	**CHB**	**NAM**	**CAN**
2q12	rs3827760	G	0	0	94	98	42
2q35	rs7559271	G	39	57	62	90	62
4q31	rs12644248	G	0	2	15	46	26
4q31	rs2045323	A	9	3	18	66	34
6p21	rs1852985	T	13	20	24	55	30
7p13	rs17640804	T	72	82	96	36	61
20p11	rs927833	C	92	36	95	59	77

SNP, single-nucleotide polymorphism.

^*^CEU, YRI, CHB are Europeans, Yoruba and Chinese from the 1000 genomes project. NAM are Native Americans and CAN is the CANDELA sample examined here. NAM data are from populations included in Reich et al.^70^.
