# Supplementary material for: A genome-wide association scan implicates DCHS2, RUNX2, GLI3, PAX1 and EDAR in human facial variation
Source: Nat Commun. 2016 May 19;7:11616. doi: 10.1038/ncomms11616 (PMC4874031; doi:10.1038/ncomms11616)
Supplement: Supplementary Information — Supplementary Figures 1-14, Supplementary Tables 1-17 and Supplementary References [file ncomms11616-s1.pdf]

SUPPLEMENTARY FIGURES

Supplementary Figure 1: Frequency distribution of 14 ordinal face traits in the CANDELA sample

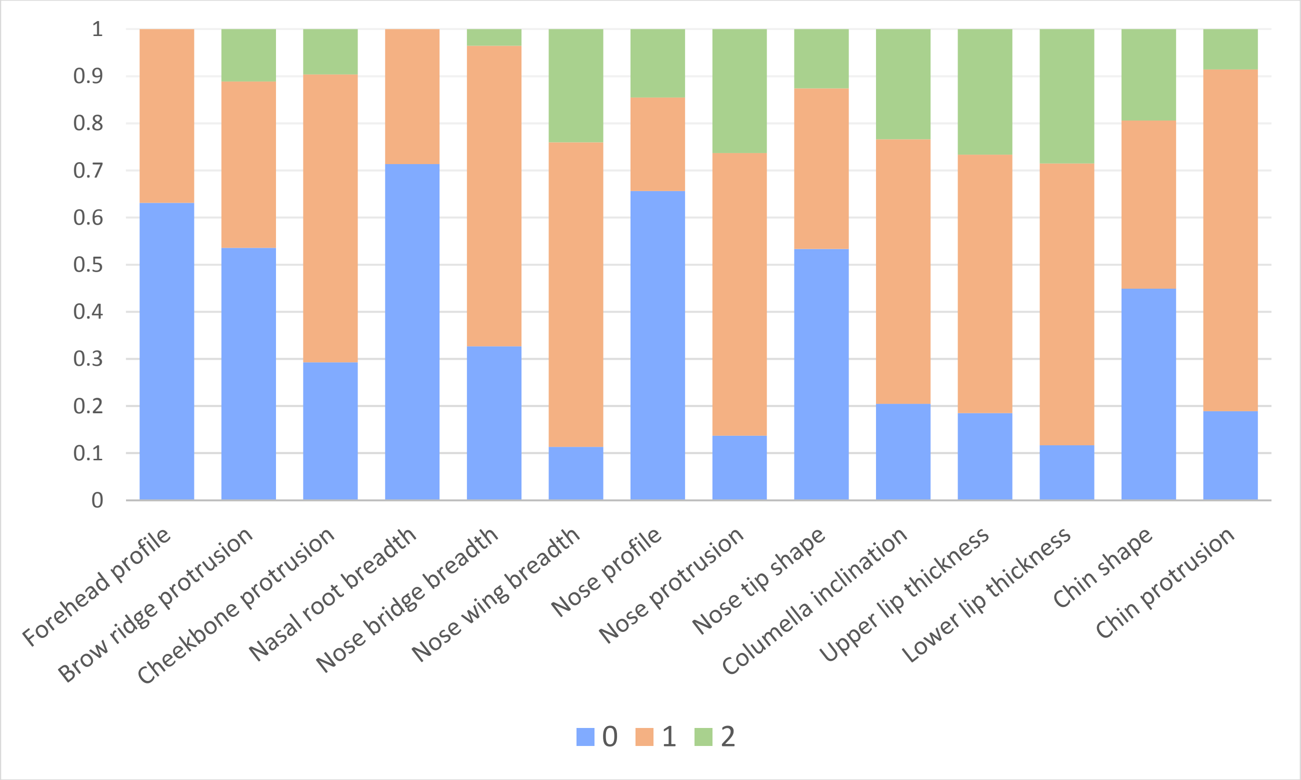

Supplementary Figure 2: Estimated African, European and Native American ancestry (%) in the CANDELA individuals included in the GWAS for ordinal face traits (N=5,958)

Individual ancestry barplots for each country are shown below. Individuals within each country are sorted by increasing European ancestry.

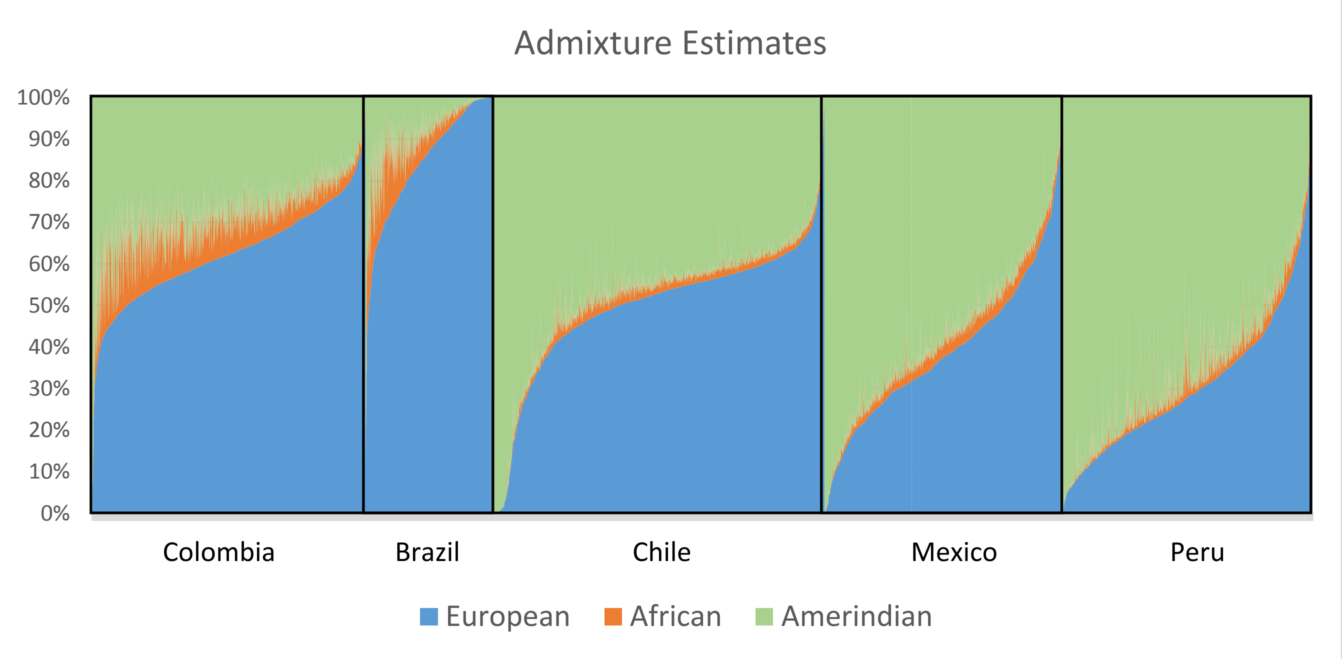

Mean ancestry estimates for each country and overall are:

	African	European	Amerindian
Colombia	8.7%	62.2%	29.1%
Brazil	6.7%	84.1%	9.2%
Chile	2.5%	49.4%	48.2%
Mexico	3.5%	38.9%	57.6%
Peru	3.5%	30.8%	65.8%
Overall	4.7%	50.1%	45.2%

Supplementary Figure 3: Selection of genetic Principal Components for inclusion in the GWAS analyses

A) Scree plot:

The proportion of variance explained by each PC is shown below.

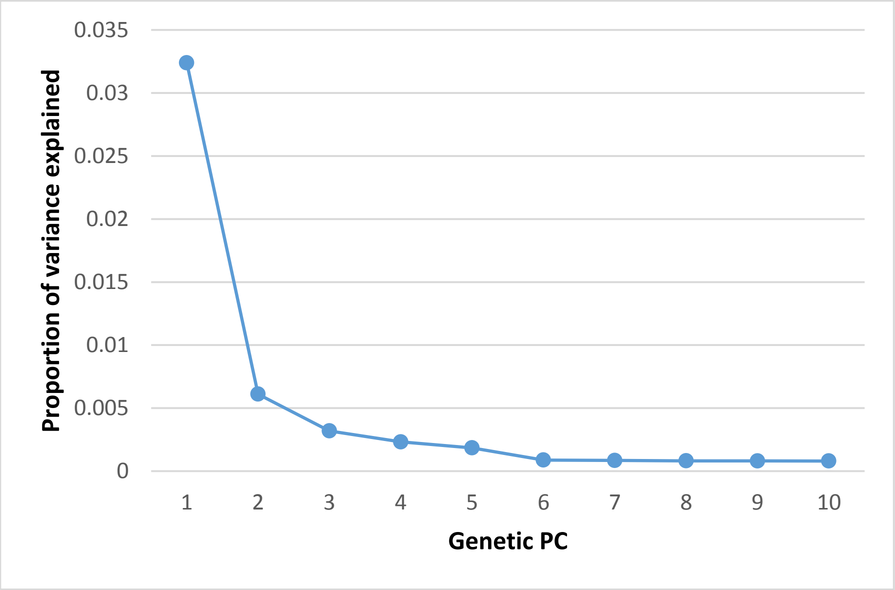

B) Correlation of genetic PCs and ancestry:

Correlations of the top 5 genetic PCs with the three continental ancestries (Supplementary Figure 2) are presented in the table below. Since European-Amerindian admixture is the main genetic component in our samples, PC1 is highly correlated with these ancestries with opposite signs. PC2 is highly correlated with African ancestry.

Ancestry	PC1	PC2	PC3	PC4	PC5
European	0.951	-0.307	0.001	-0.025	-0.008
Amerindian	-0.998	0.056	0.012	0.005	0.010
African	0.379	0.920	-0.053	0.072	-0.013

C) GWAS Q-Q plots:

Nose profile:

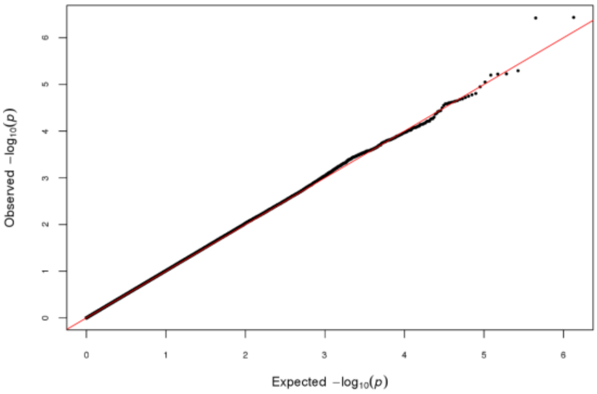

Forehead profile:

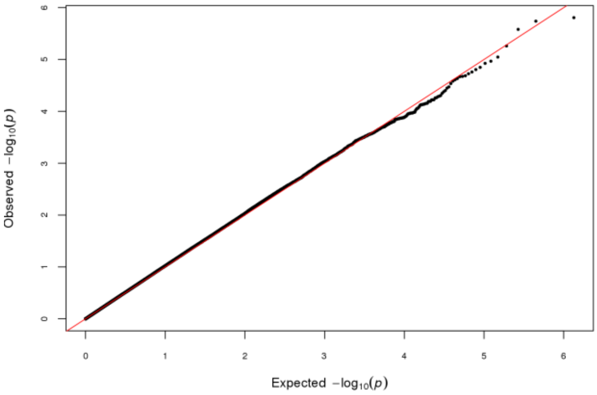

Brow ridge protrusion:

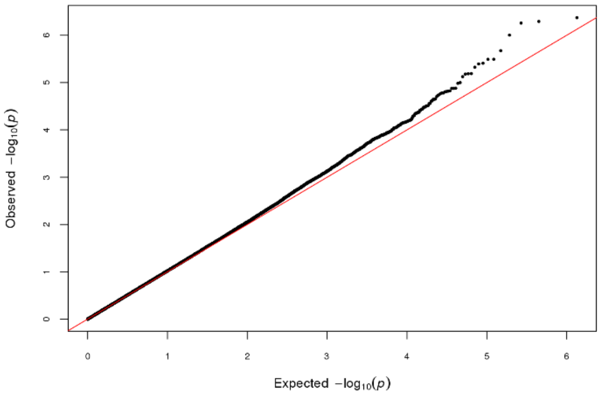

Cheekbone protrusion:

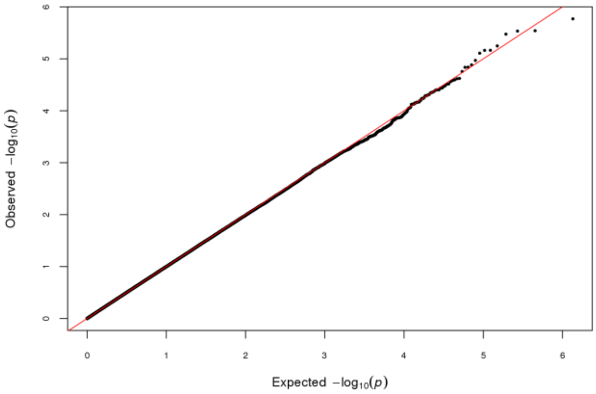

Nasal root breadth:

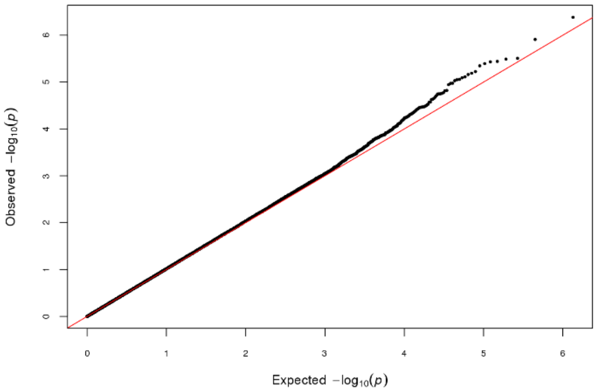

Nose bridge breadth:

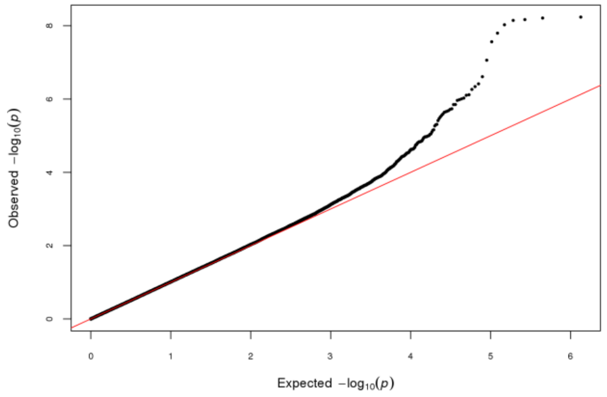

Nose wing breadth:

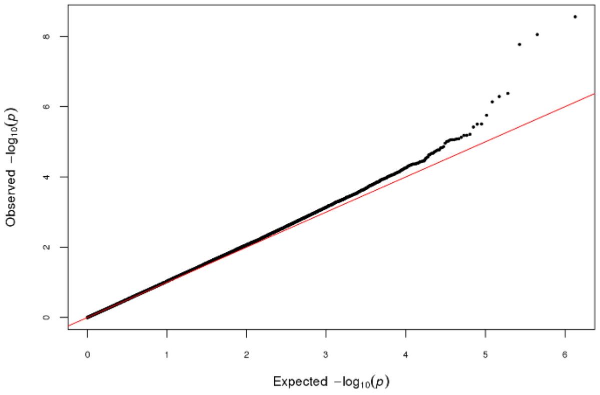

Nose protrusion:

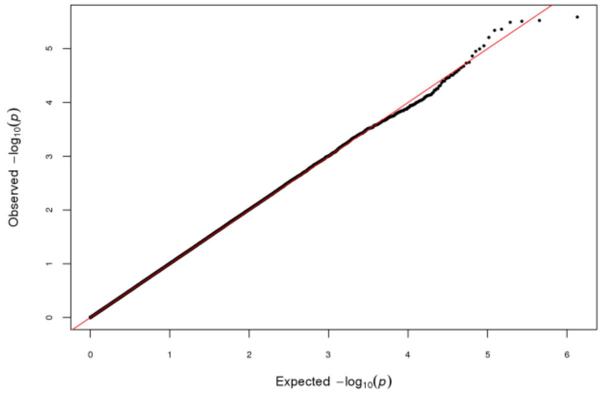

Nose tip shape:

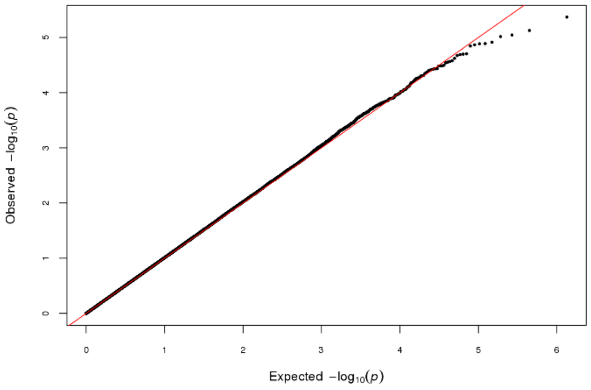

Columella inclination:

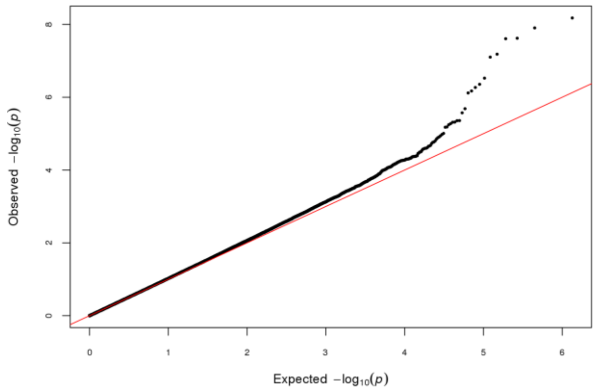

Upper lip thickness:

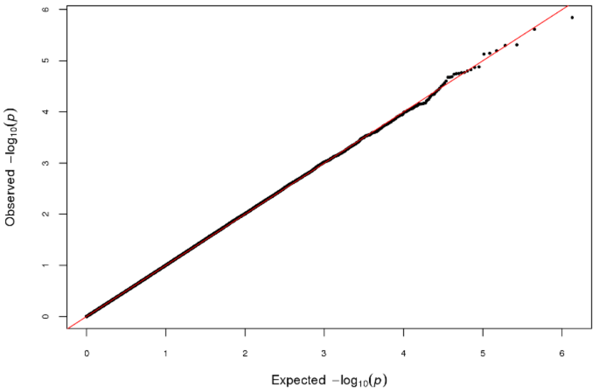

Lower lip thickness:

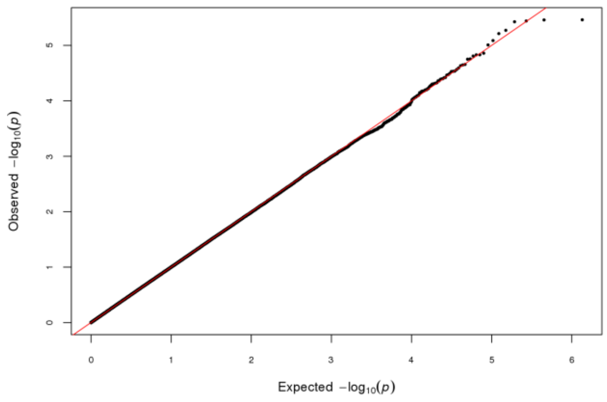

Chin shape:

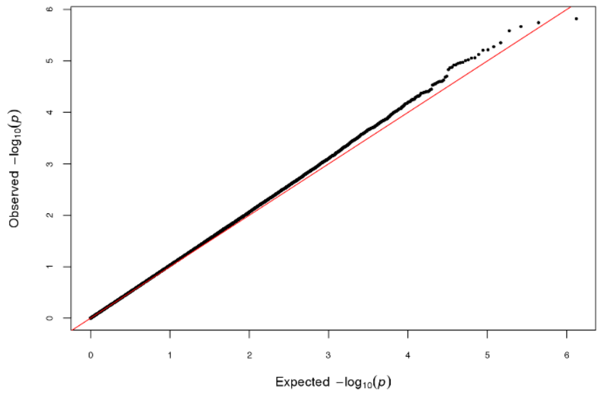

Chin protrusion:

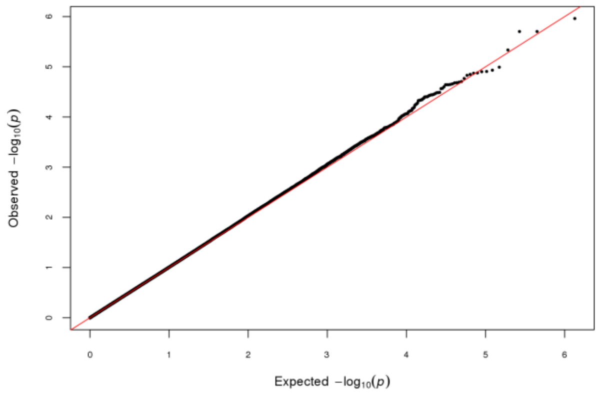

D) Genomic inflation factor for categorical traits:

Trait	Genomic Inflation Factor λ
Forehead profile	1.012
Brow ridge protrusion	1.002
Cheekbone protrusion	1.014
Nasal root breadth	1.013
Nose bridge breadth	1.008
Nose wing breadth	1.009
Nose profile	1.007
Nose protrusion	1.014
Nose tip shape	1.004
Columella inclination	1.015
Upper lip thickness	1.001
Lower lip thickness	1.002
Chin shape	1.016
Chin protrusion	1.014

Supplementary Figure 4: Features of the replication sample

A) Frequency distribution of categorical phenotypes

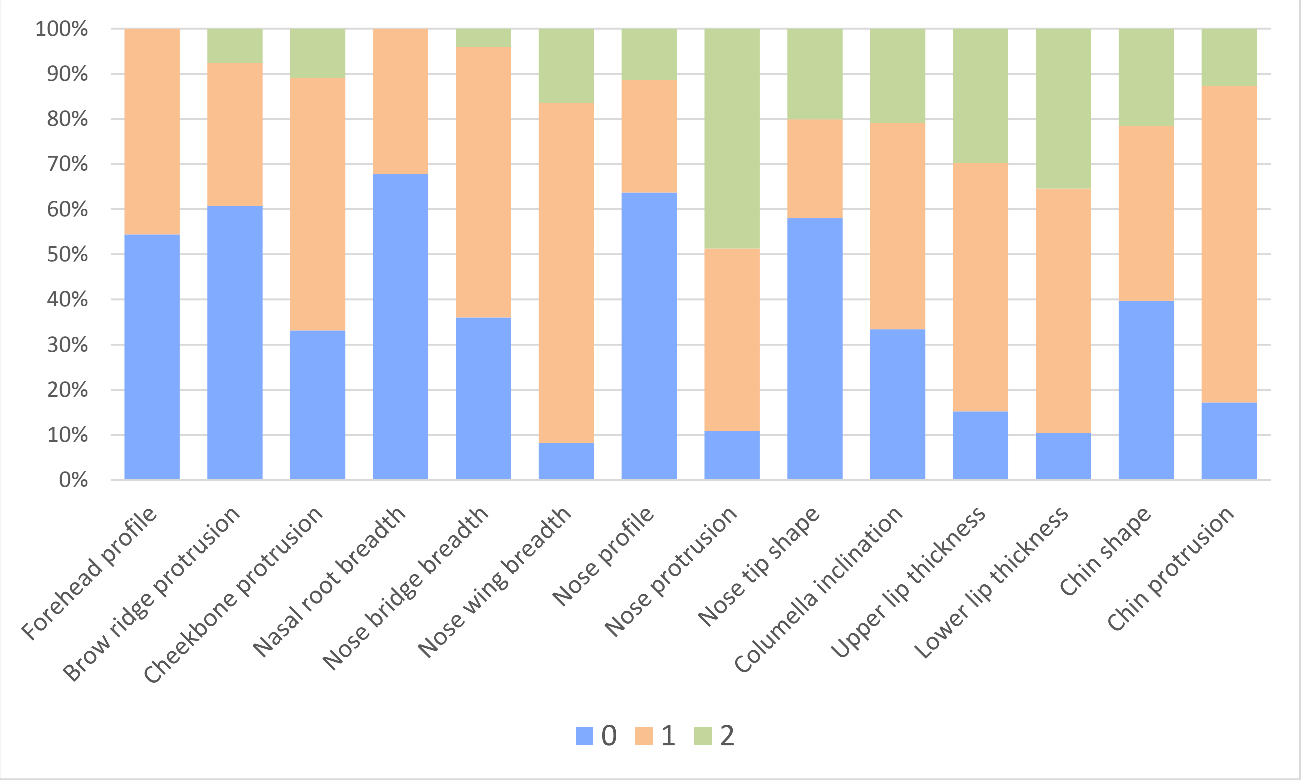

B) Individual ancestry

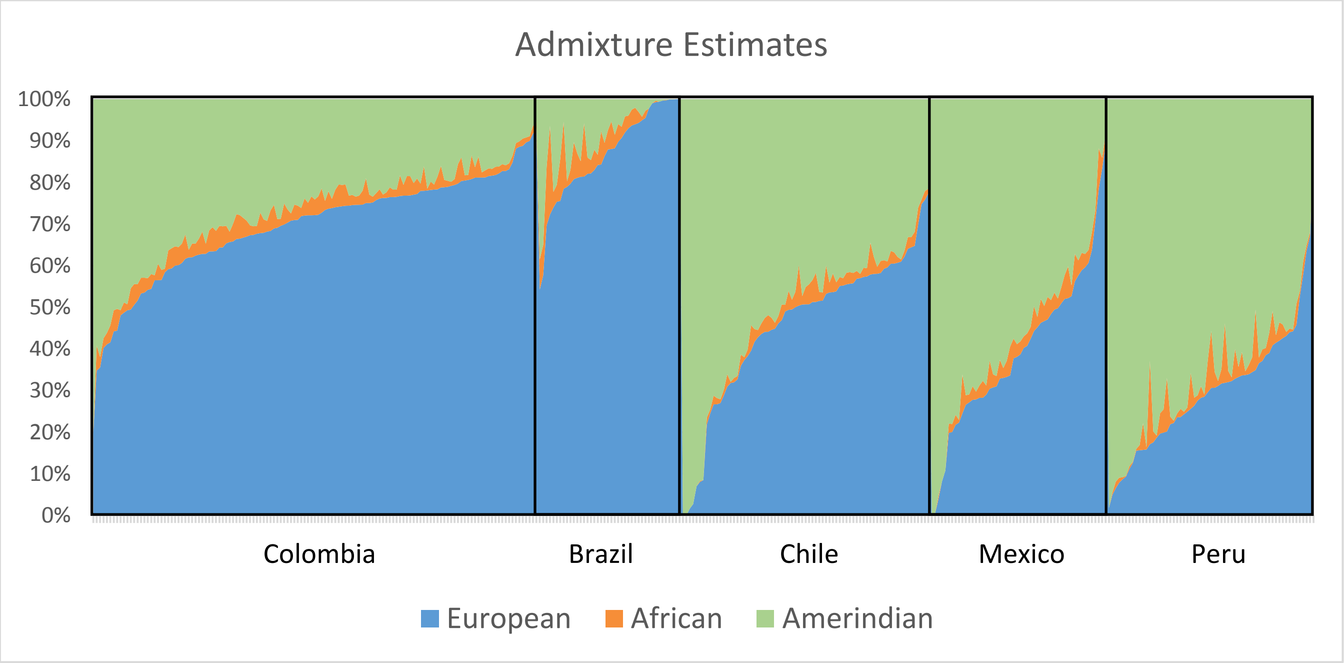

Mean ancestry estimates for each country and overall are:

	African	European	Amerindian
Colombia	3.2%	69.4%	27.4%
Brazil	4.5%	86.7%	8.9%
Chile	2.5%	45.7%	51.9%
Mexico	3.5%	39.5%	57.0%
Peru	3.4%	29.9%	66.7%
Overall	3.3%	55.6%	41.2%

C) Sample size and basic covariates

	Total	Colombia	Brazil	Chile	Mexico	Peru
**Sample size**	501	182	59	102	73	85
**Percentage**	100	36.3	11.8	20.4	14.6	17.0
**% Female**	61.1	56.7	66.7	61.3	77.8	70.0
**Age (years)**						
Min	18	18	18	18	18	18
Mean	25.7	24.7	26.6	31.3	26.2	21.3
Max	45	45	41	44	40	30
S.D.	7.2	6.3	6.8	9.5	6.2	3.1
**Age, for Males**						
Min	18	18	18	18	18	18
Mean	26.3	25.9	26.2	30.5	25.8	20.7
Max	45	45	37	42	37	28
S.D.	7.3	7.0	7.3	7.7	7.5	3.4
**Age, for Females**						
Min	18	18	18	18	18	18
Mean	25.3	23.8	26.8	31.7	26.3	21.6
Max	45	44	41	44	40	30
S.D.	7.2	5.5	6.9	10.5	5.9	2.9

Supplementary Figure 5: Facial landmarking protocols

A) 3-D landmarking

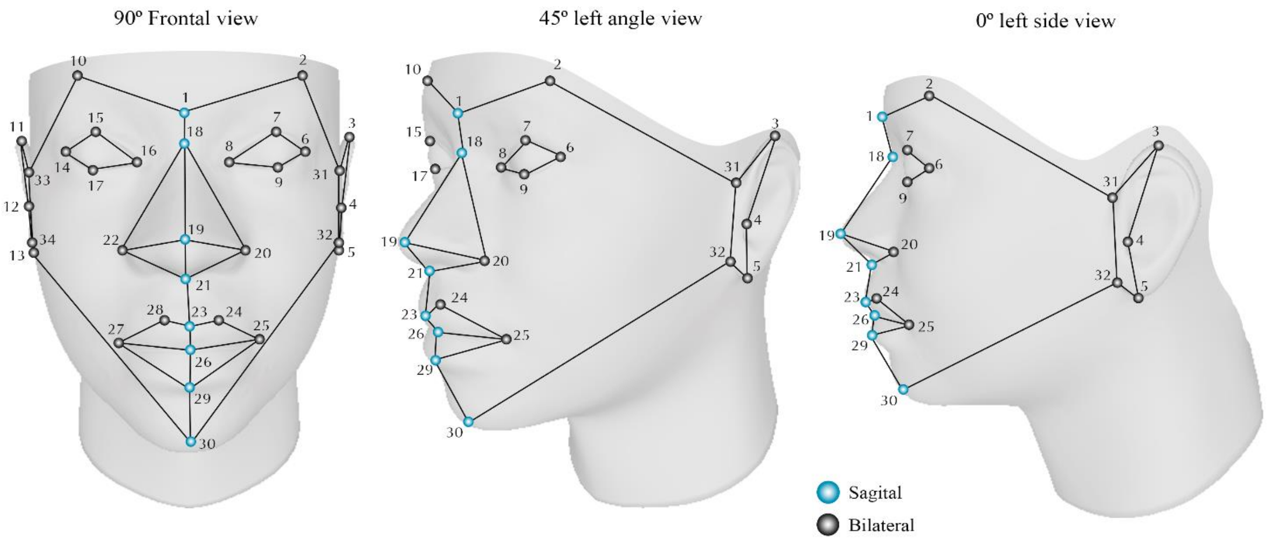

34 anatomical 3-D landmarks placed in photographs from 2,955 individuals from the CANDELA sample [1] were examined here.

B) 2-D landmarking

On frontal photographs, 2 landmarks were placed for nasal root (3,4) and for nose bridge width (5,6), in addition to the major frontally visible landmarks from the 3-D landmarking protocol. The 3-D landmarks were used to calibrate the 2-D derived distances.

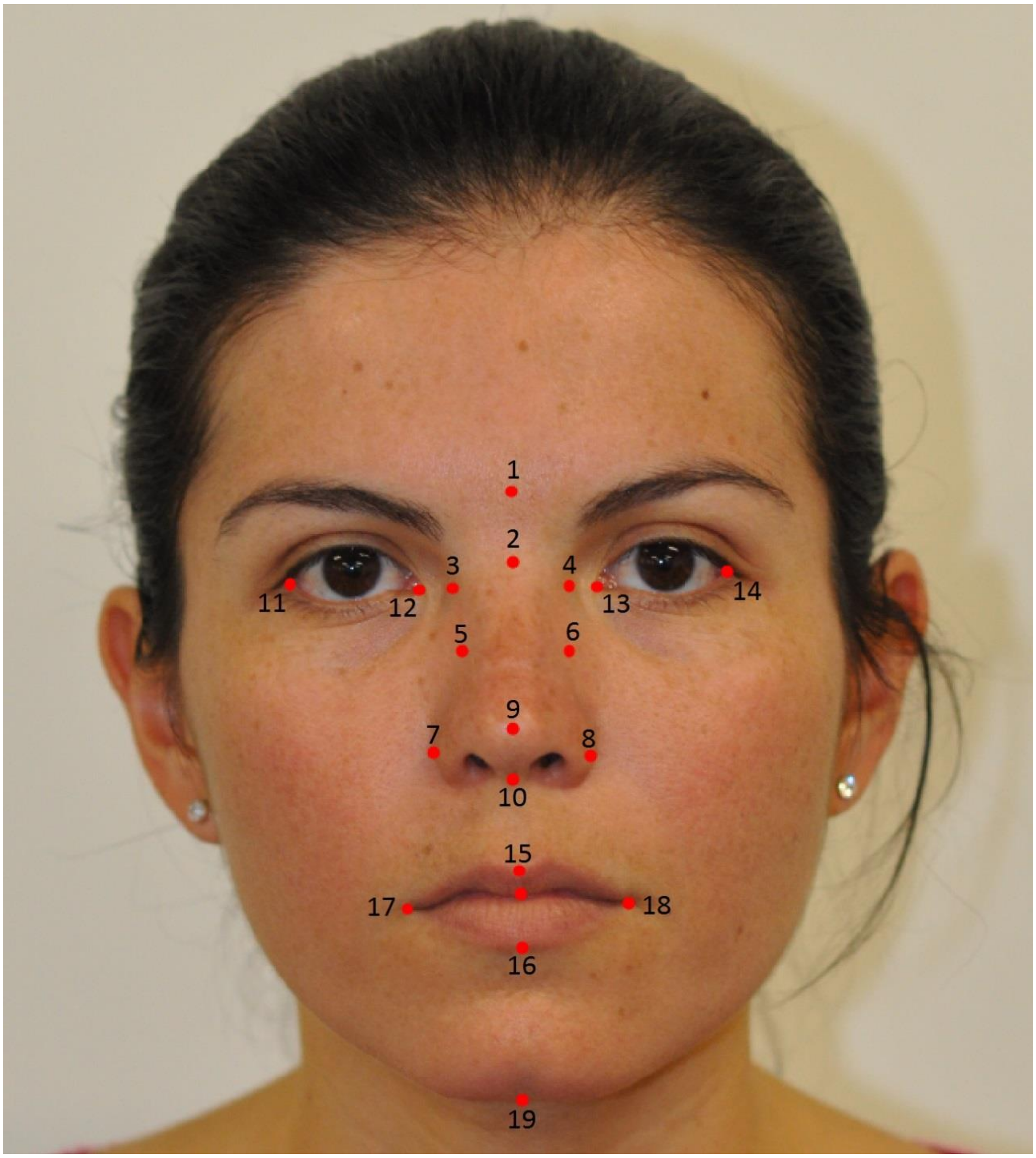

Supplementary Figure 6: Boxplots for categorical vs. quantitative traits

In each of the plots below, the top panel shows the boxplot of a quantitative trait versus its corresponding categorical trait. The lower panel shows a histogram of the categorical trait. The categories are color-coded.

A) Nasal root breadth:

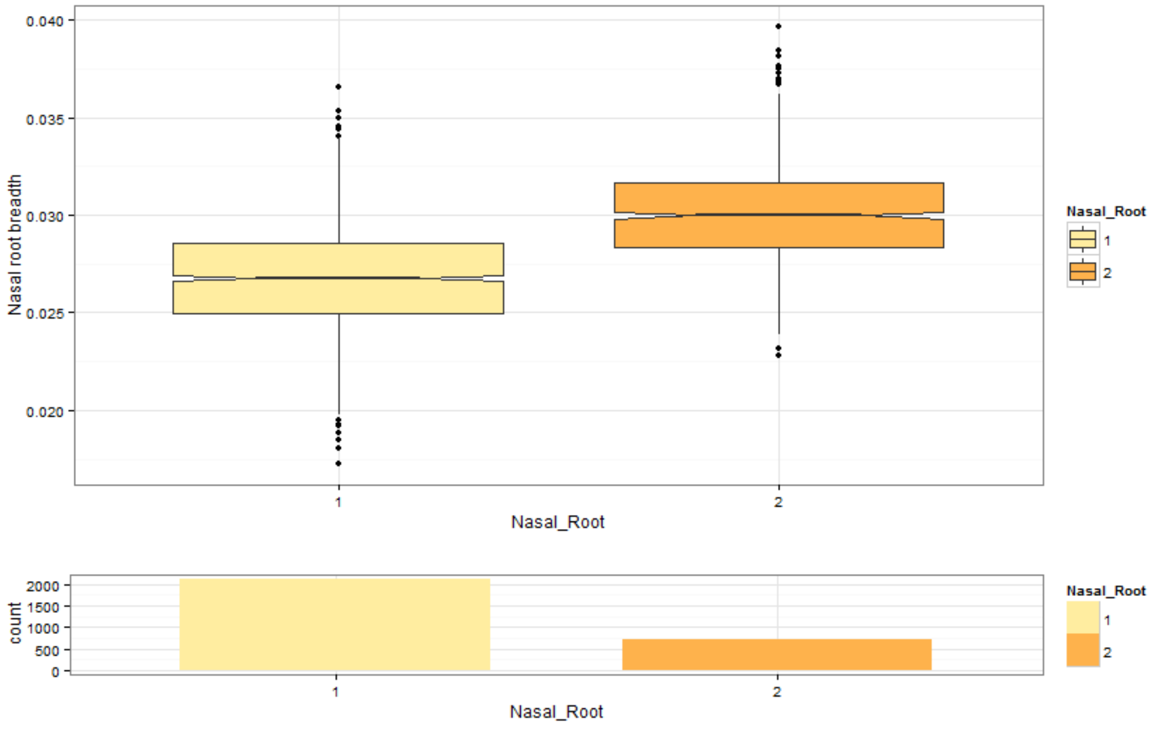

B) Nose bridge breadth:

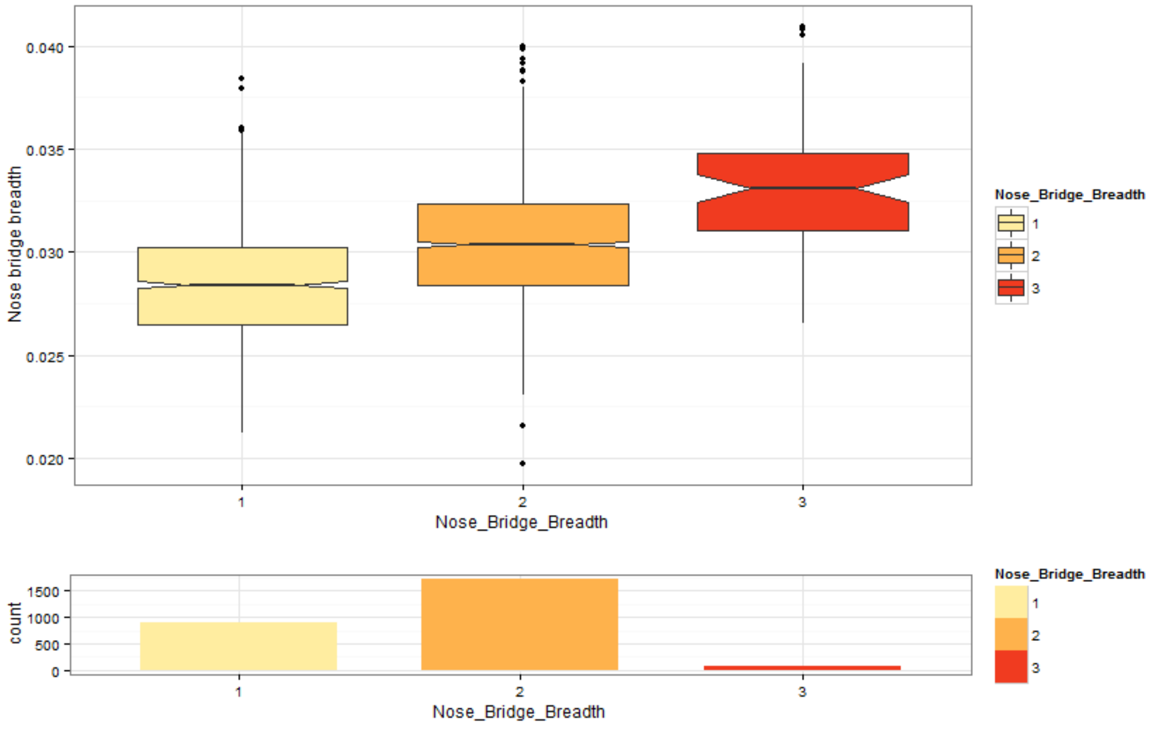

C) Nose wing breadth:

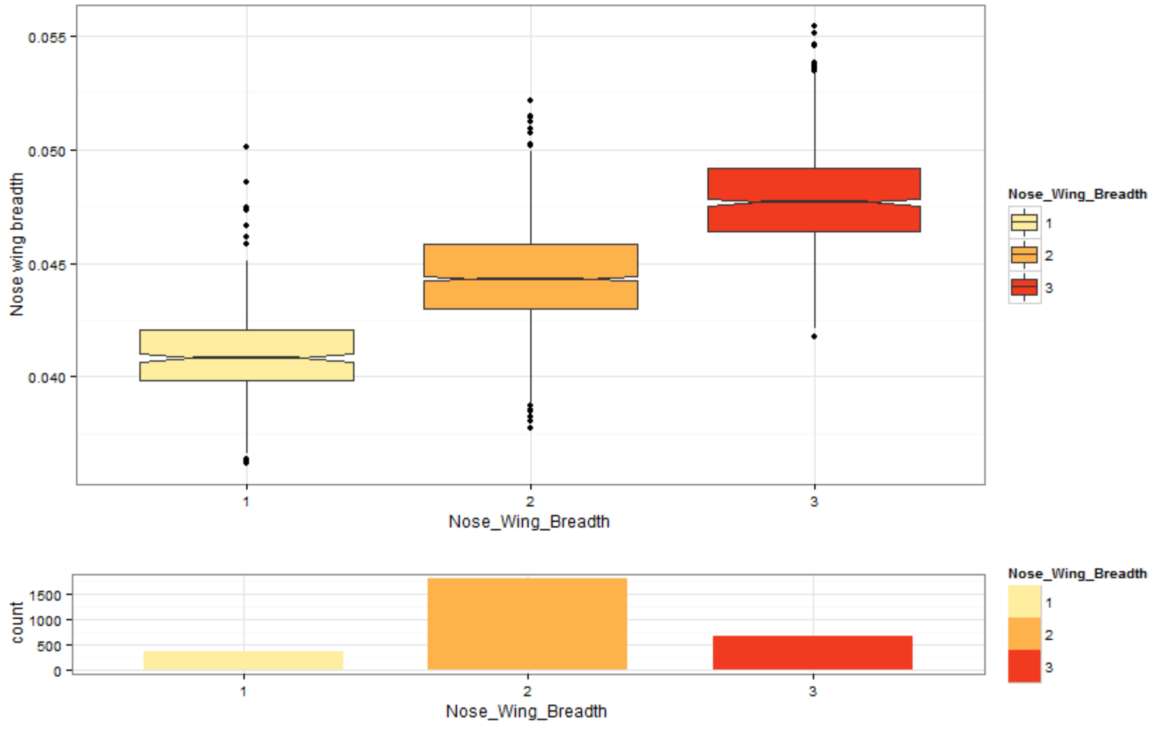

D) Nose protrusion:

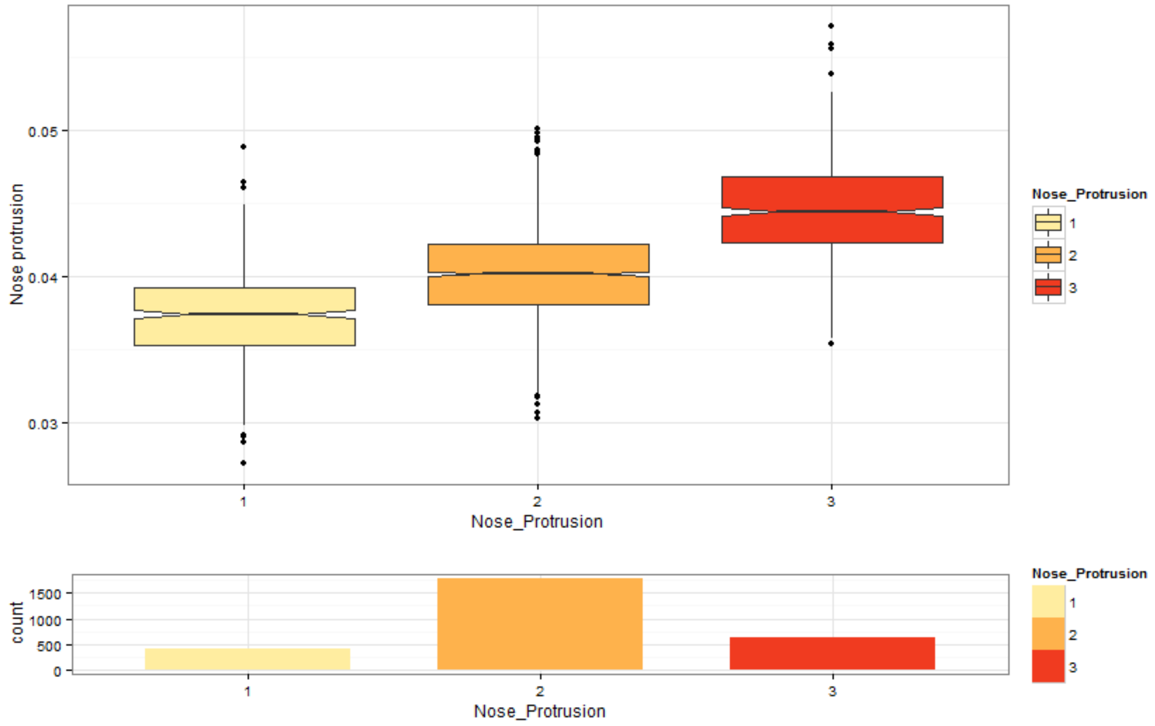

E) Nose tip angle:

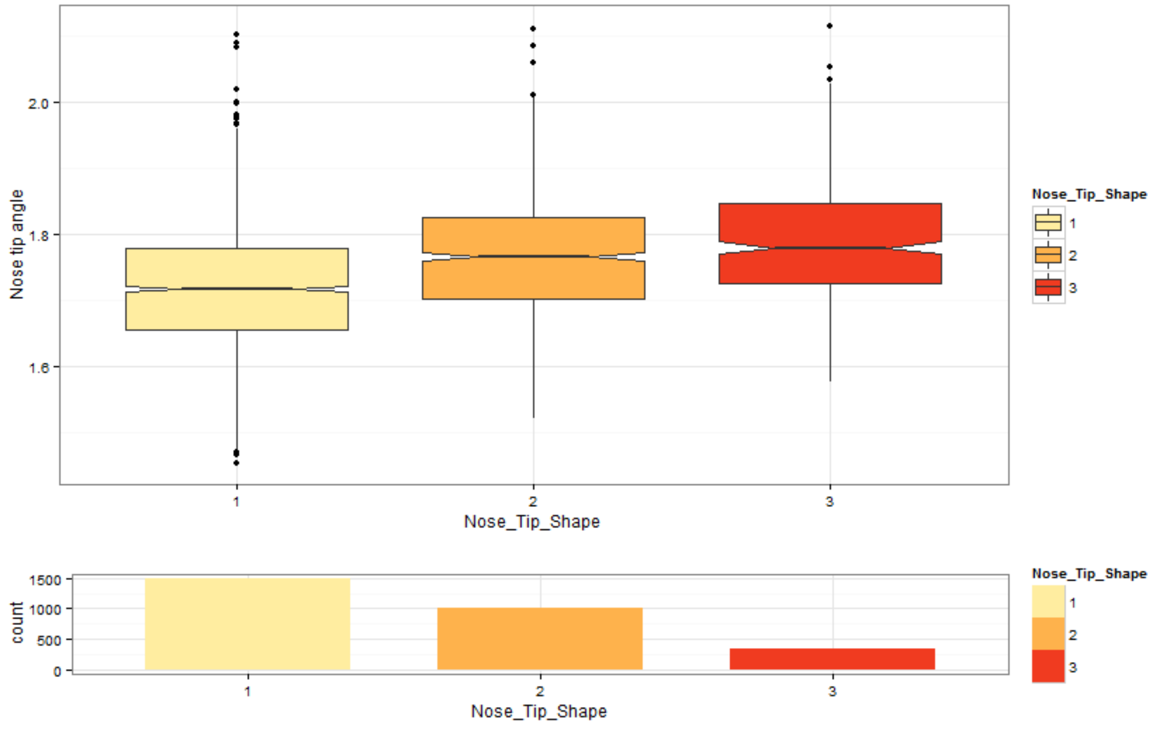

F) Columella inclination:

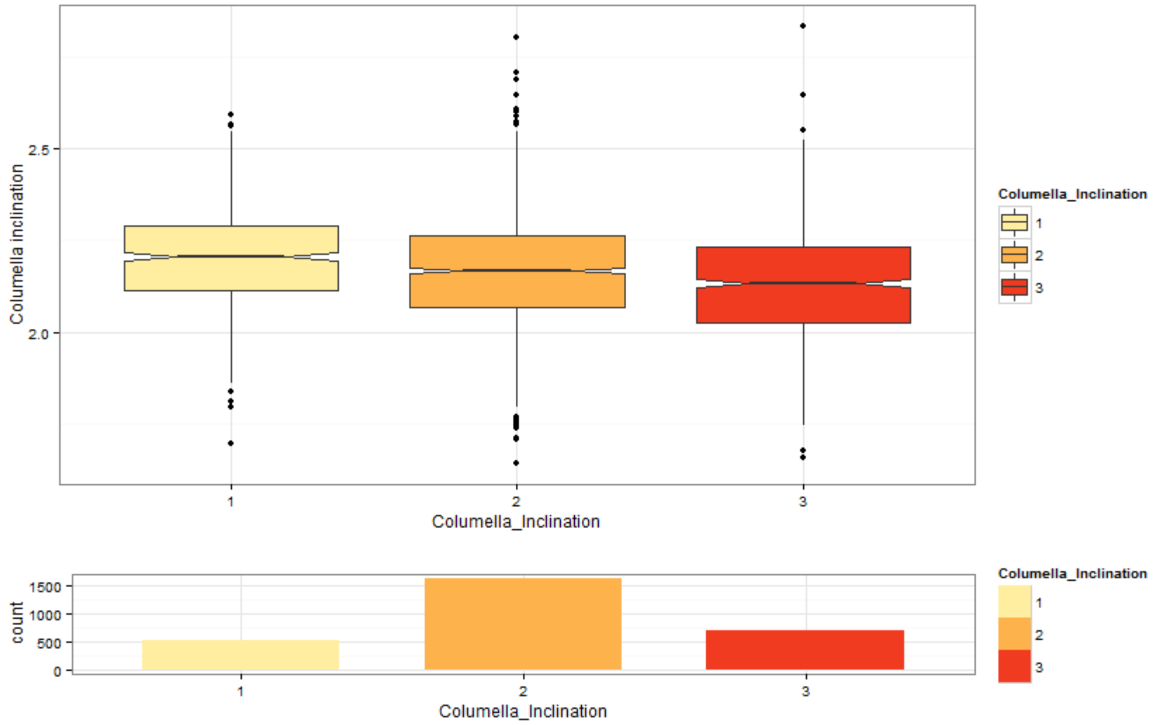

G) Upper lip thickness:

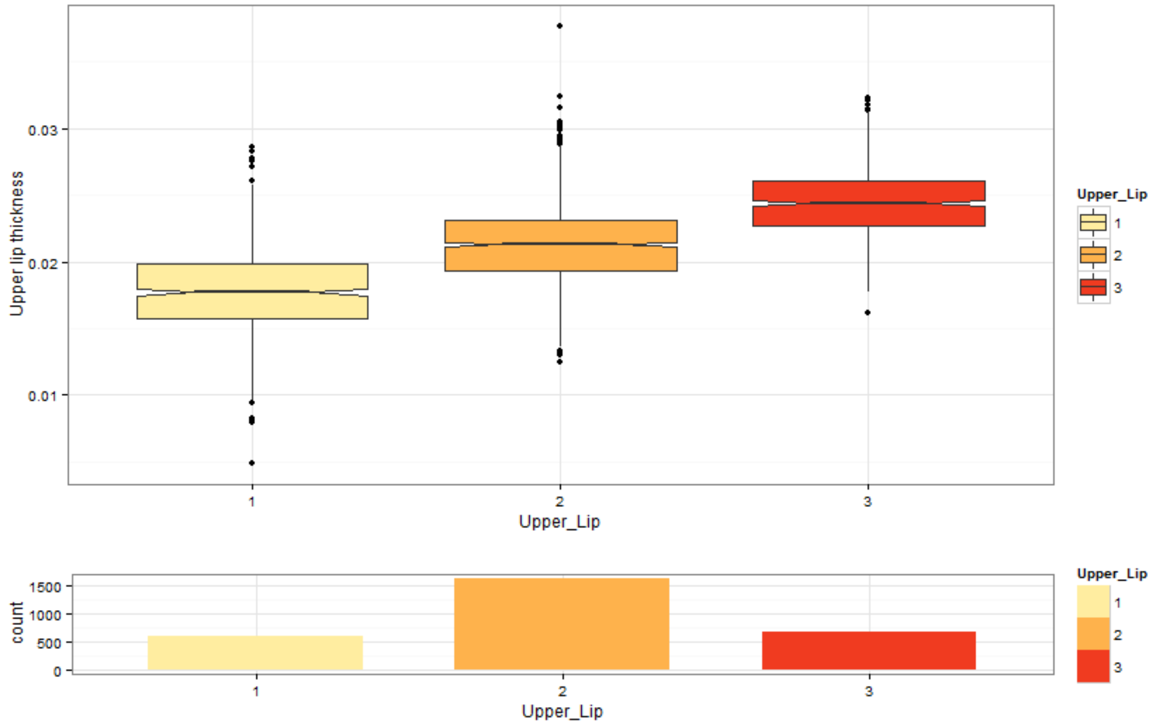

H) Lower lip thickness:

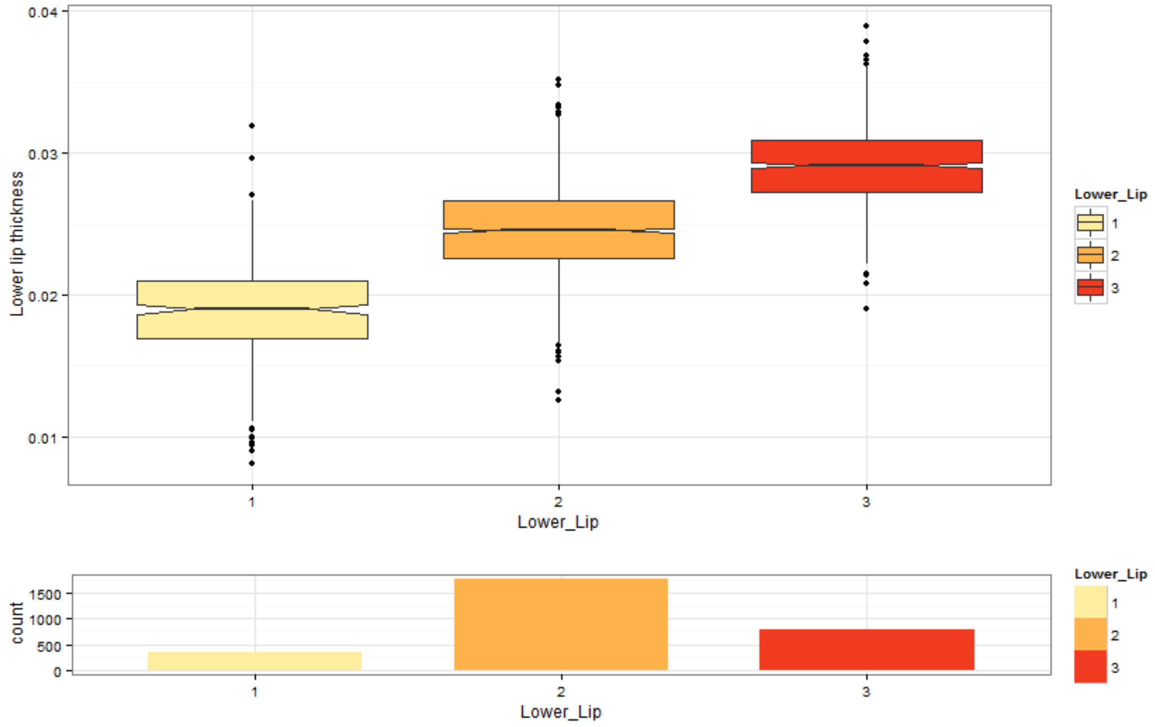

I) Chin protrusion:

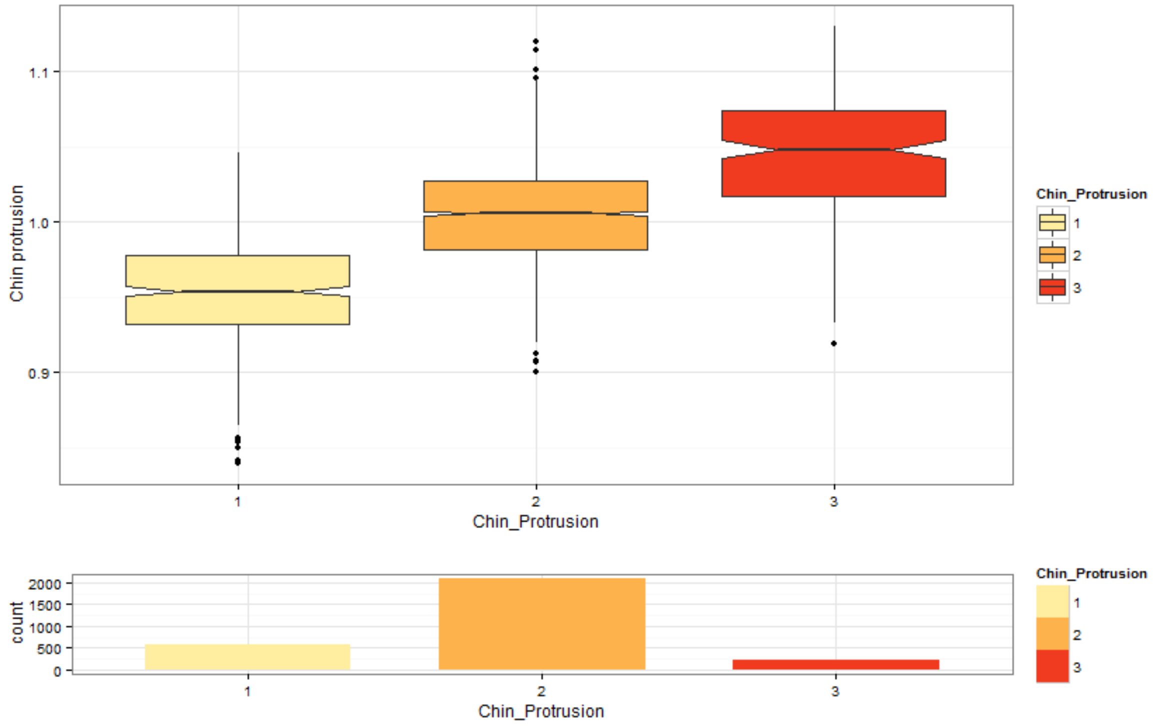

Supplementary Figure 7: Regional association plots not shown in Figure 3.

A) 2q35 and nasion position:

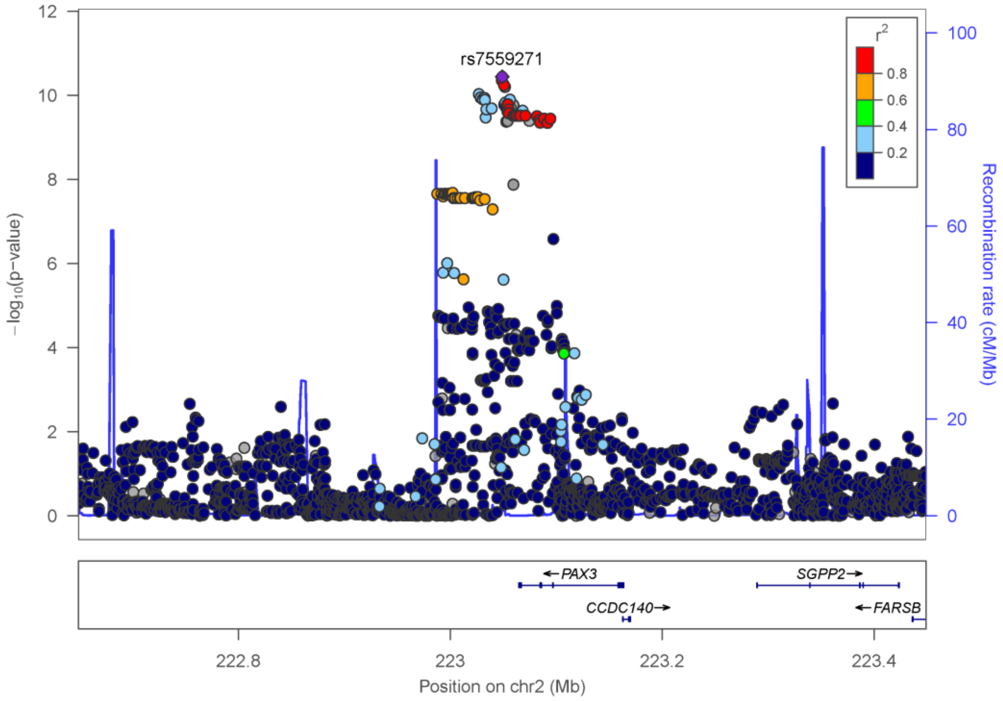

B) 4q31 and quantitative assessment of nose protrusion:

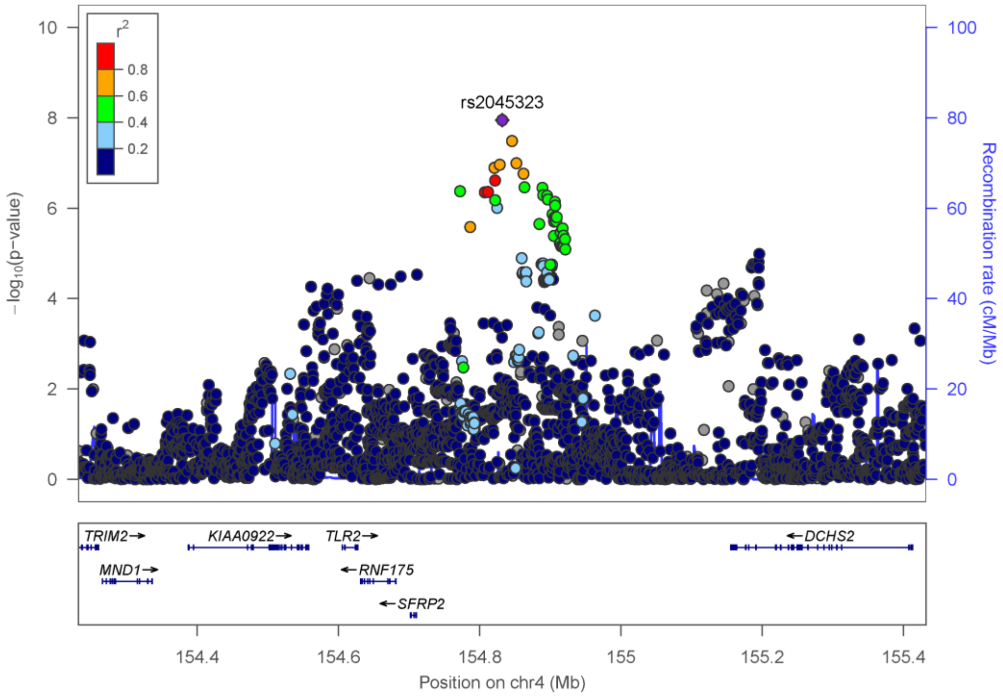

C) 4q31 and quantitative assessment of nose tip angle:

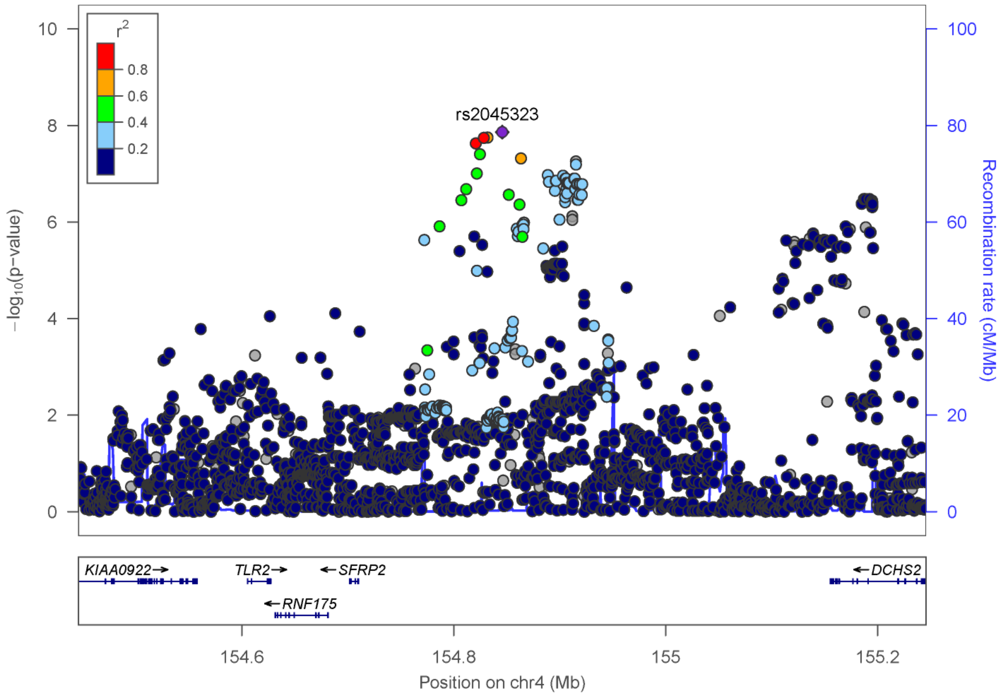

D) 6p21 and quantitative assessment of nose bridge breadth:

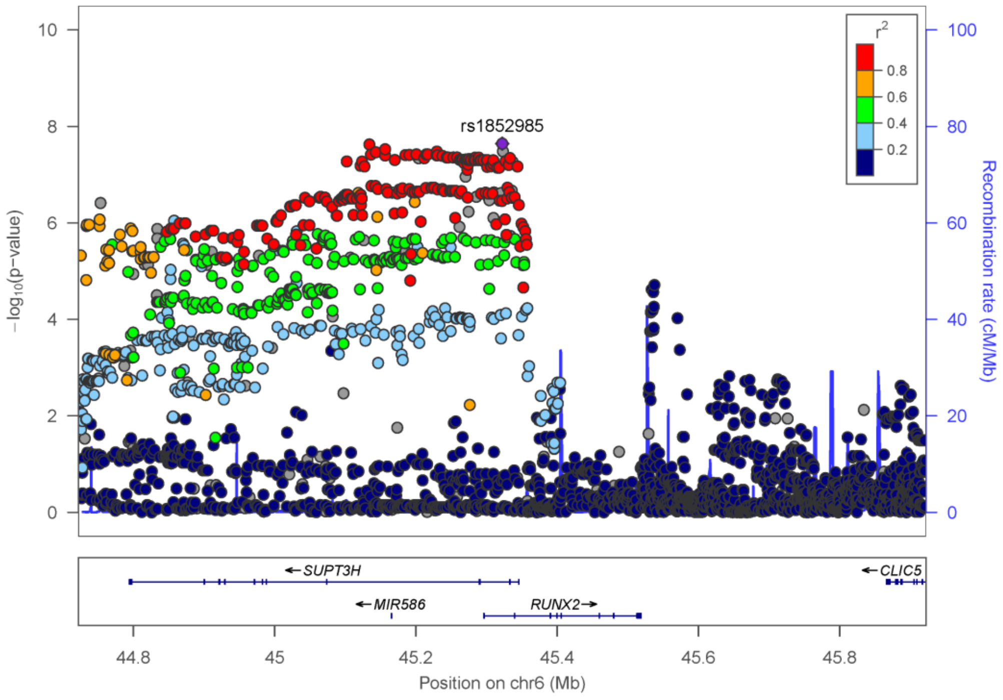

E) 7p13 and ordinal assessment of nose wing breadth:

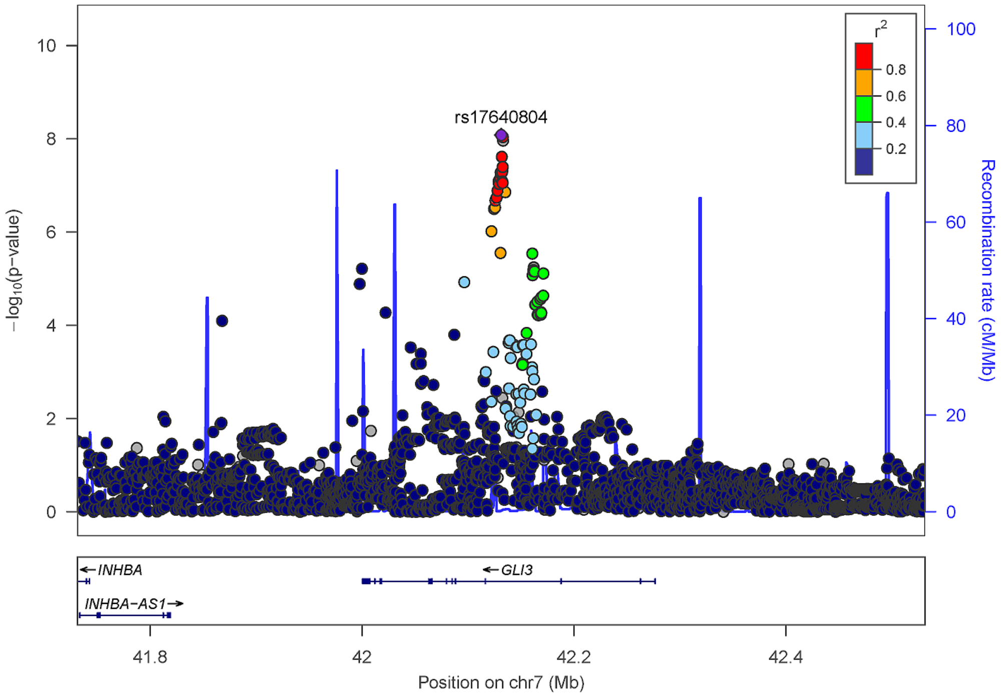

Supplementary Figure 8: Measurement of mouse mandible length

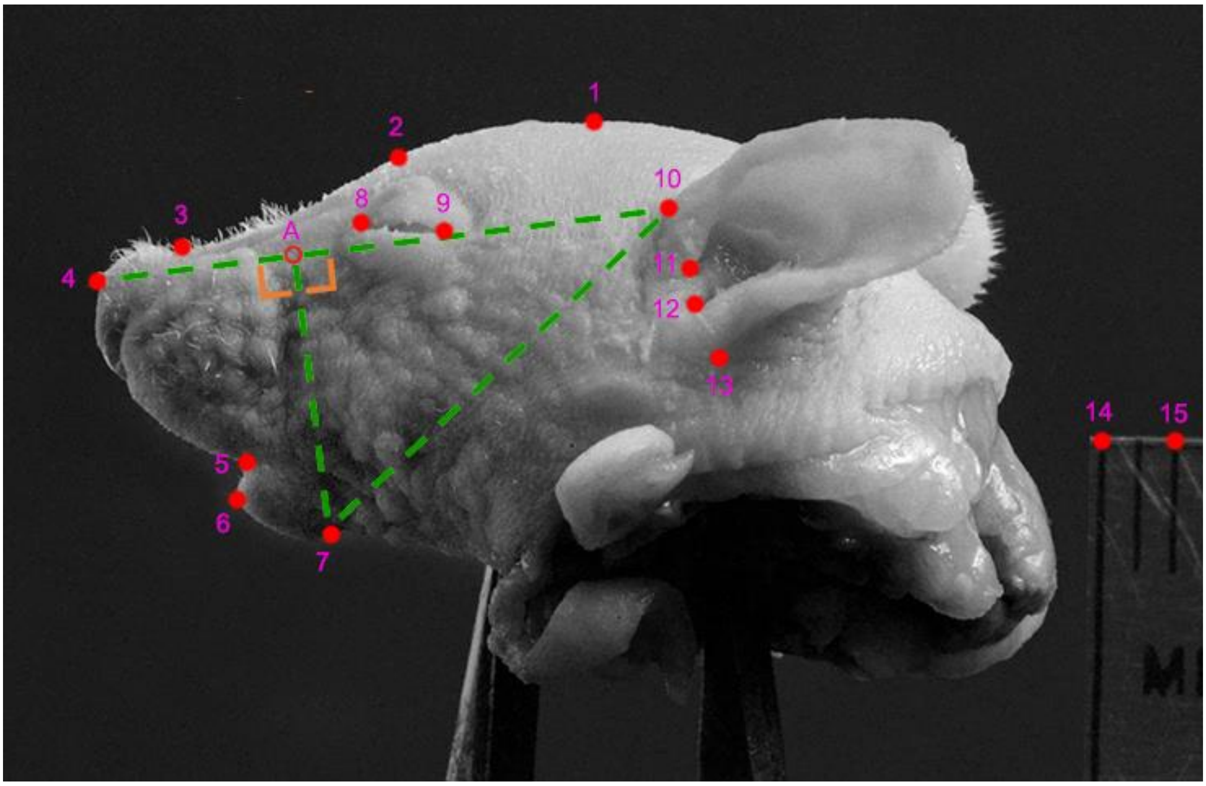

Each mouse head was mounted horizontally on spikes and the camera was positioned parallel to this plane. Photographs were taken from the left side. A scale was included in each photo for calibration. Thirteen landmarks were placed on the mouse head and two on the scale, as indicated by filled red circles. Of these, three landmarks were on the mandible – landmarks 5-7. Landmark 10 at the top of the ear was taken as reference for estimating mandible length. Direct distances were computed between this landmark and each mandible landmark (5-7). A front-to-back line of reference was drawn between landmarks 10 and 4 (tip of the snout). The projection of each mandible landmark onto this line was calculated. E.g. in the example shown above, mandible landmark 7 is perpendicularly projected onto the line joining landmarks 4 and 10, the projection point denoted by empty red circle A. The “projected distance” is the distance between this point A and landmark 10. Distances are obtained in pixel units and then converted to millimetres by calibrating with reference to the distance on the scale (between points 14 and 15). In addition, a measure of head length was obtained directly on the animal heads between landmarks 4 and 12.

Supplementary Figure 9: Mandible length in *Edar* mutant mice

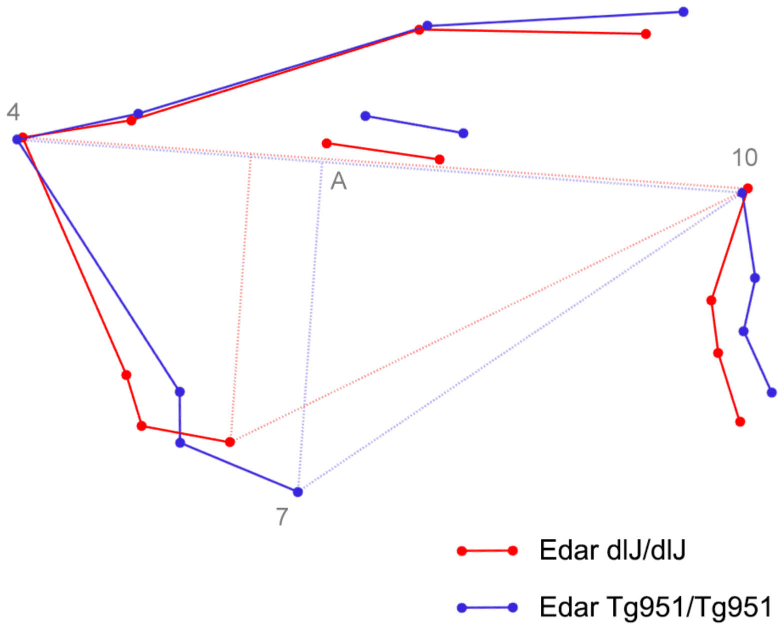

The image shows average wireframes for homozygous *Edar*$^{dlJ/dlJ}$ (red lines) and *Edar*$^{Tg951/Tg951}$ (blue lines) homozygous mice based on the 13 landmarks places on mouse heads (Supplementary Figure 8). Landmarks 4, 7, 10 and A have been labelled. Direct distance between landmarks 7 and 10 is the length of the dotted line joining them. Projected distance between landmarks 7 and 10 is the length of the dotted line between point A and landmark 10. The average wireframes for the two sets of mutants show that mandible length is smaller in *Edar*$^{Tg951/Tg951}$ mice.

Supplementary Figure 10: Conditional association analysis in 4q31 for the quantitative assessment of nose protrusion.

As seen in Figure 3b-c and Supplementary Figure 7B-C the 4q31 region shows two peaks of association for measurements of columella inclination, nose protrusion and nose tip angle. Strongest association at one peak occurs at SNP rs2045323 in the *DCHS2-SFRP2* intergenic region. The second peak has strongest association at rs12644248 in the *DCHS2* gene. This is the only genome-wide significant association peak for the ordinal assessment of columella inclination (Table 1, Figure 3). To evaluate independence of these two signals we performed regional association test conditioned on rs12644248 or on rs2045323 on the quantitative nose traits. Columella inclination, nose protrusion and nose tip angle all showed the same behaviour. Below we present the plots for nose protrusion, those for the other two traits being very similar.

A) Regional association for nose protrusion conditioned on rs12644248

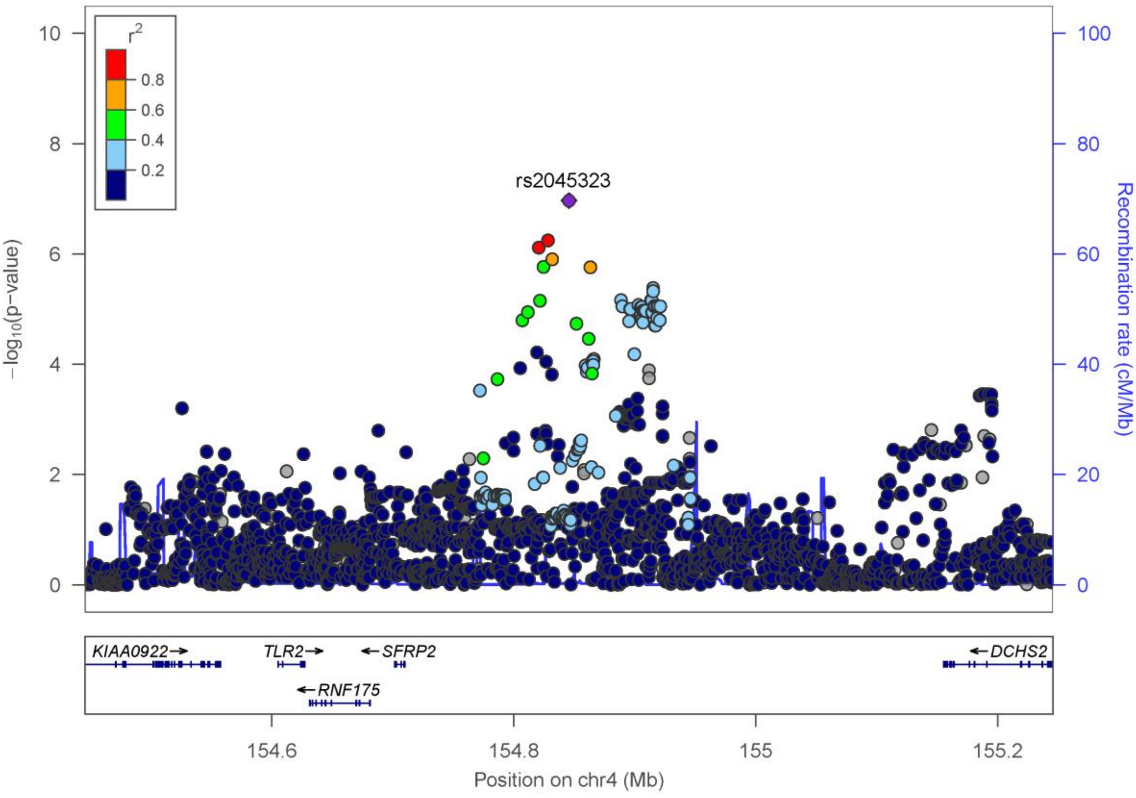

B) Regional association for nose protrusion conditioned on rs2045323.

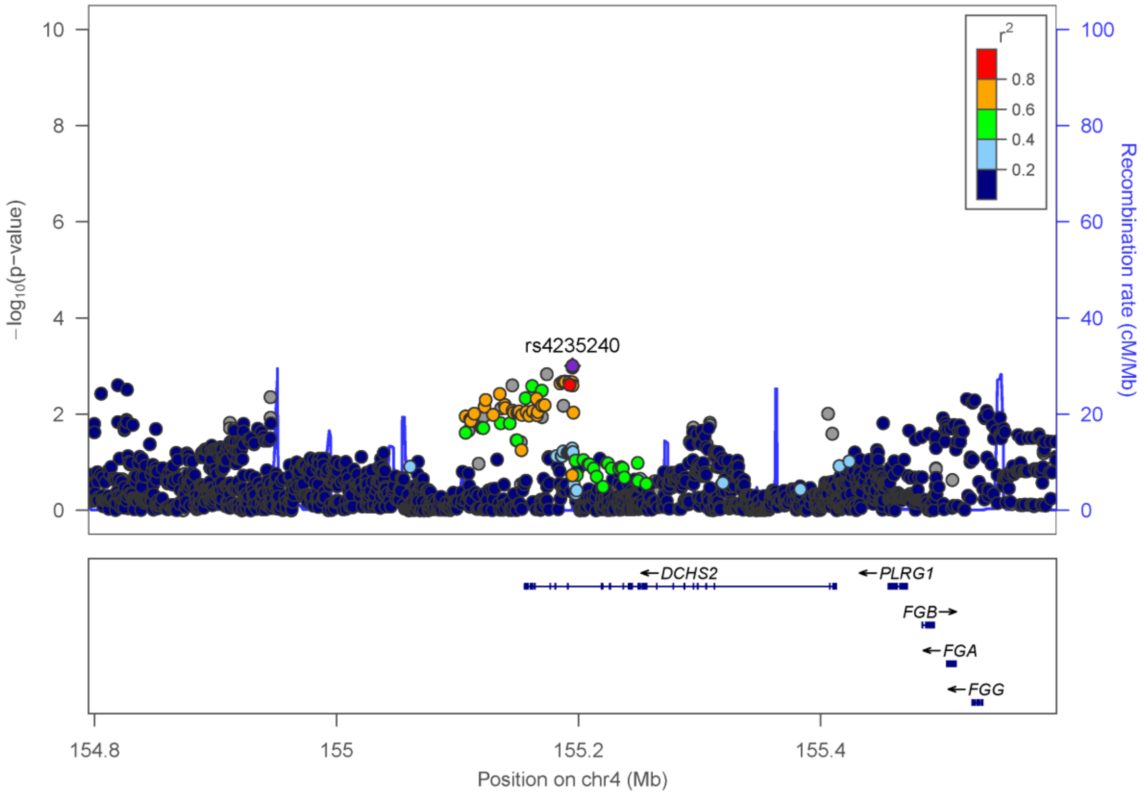

Supplementary Figure 11: Sequence conservation around rs2045323 in the *DCHS2-SFRP2* intergenic region

A UCSC genome-browser screenshot from the (GRCh37/hg19) Assembly for the *DCHS2-SFRP2* intergenic region around rs2045323 (highlighted in black at the bottom), showing enriched values of various conservation scores including GERP.

A) Full view:

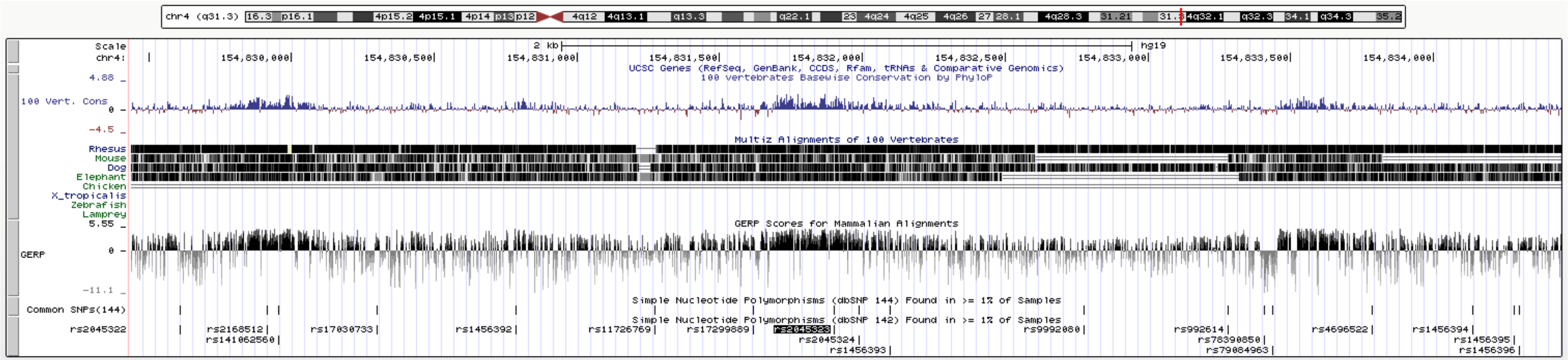

B) Cropped view:

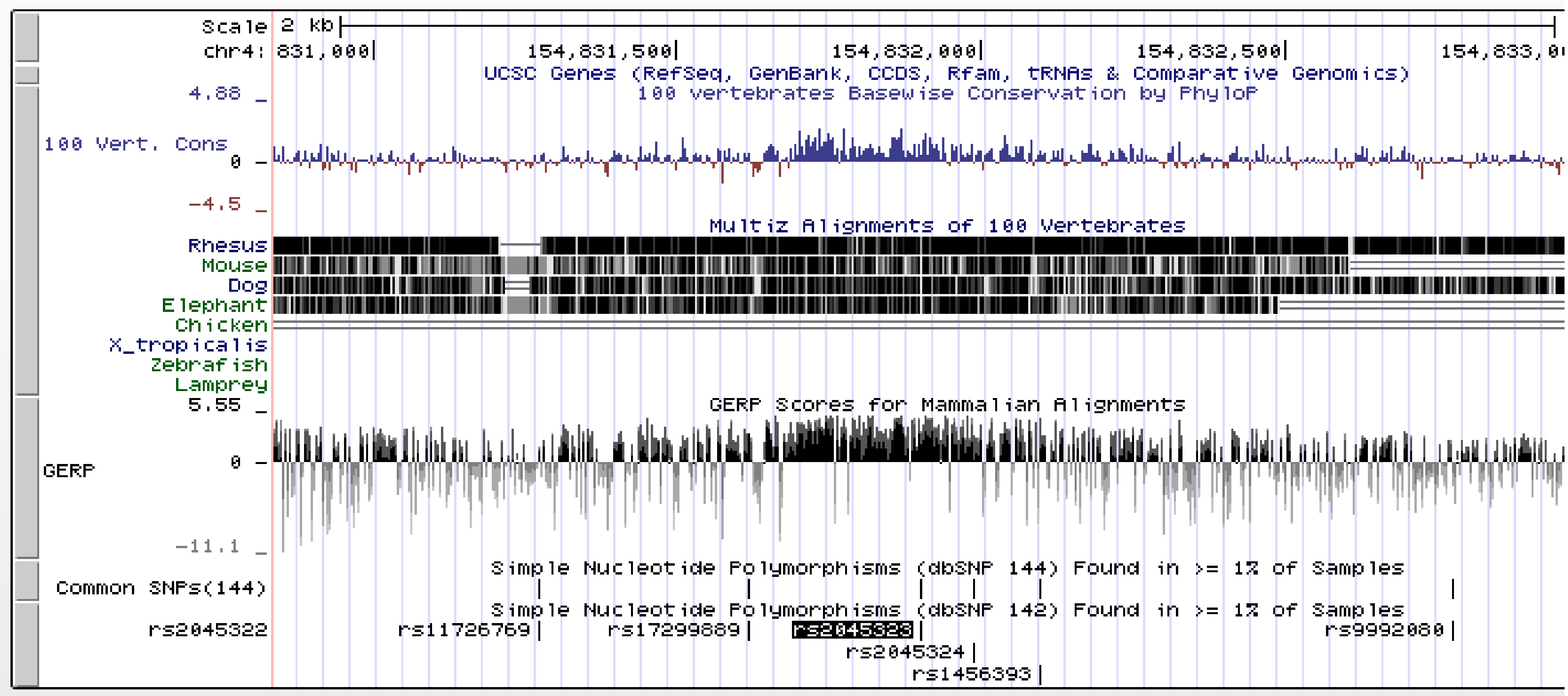

Supplementary Figure 12: SNPs in 6p21 associated with nose bridge breadth and regulatory elements in the *SUPT3H/RUNX2* gene region.

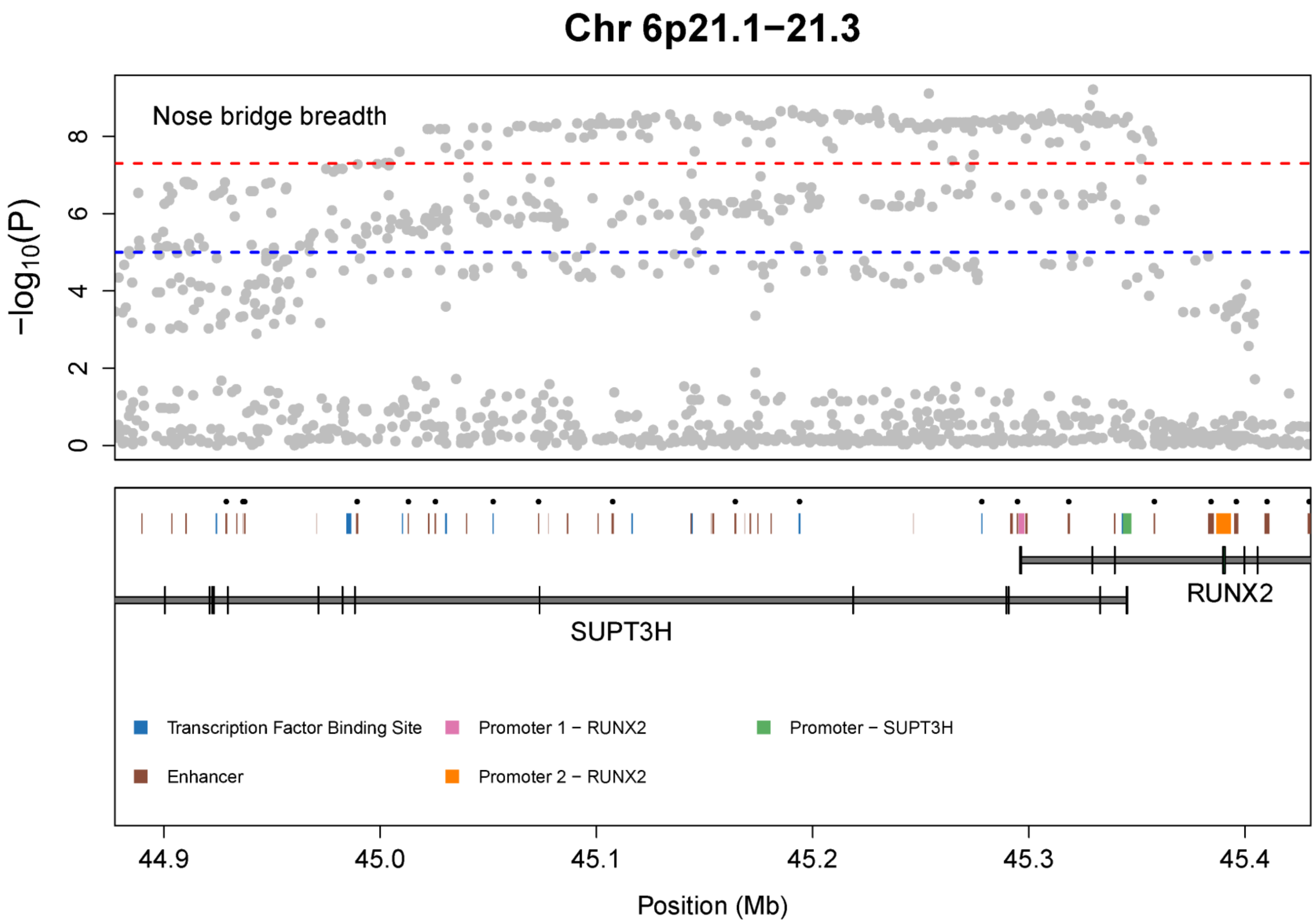

The top panel shows $-\log_{10}$ association *P* values (dashed lines indicate the genome-wide significance threshold of 7.3, and a suggestive threshold of 5). The bottom panel shows the location of exons/introns and of various regulatory elements. Genome annotations were obtained from the Ensembl Genome Bioinformatics database (build GrCh37). Regulatory annotations supported by experimental evidence [2-9] are indicated by black dots.

Supplementary Figure 13: Strong sequence conservation around rs17640804 in *GLI3*

A UCSC genome-browser screen shot from the (GRCh37/hg19) Assembly for the *GLI3* region around rs17640804 (highlighted in black at the bottom), showing enriched values of various conservation scores including GERP.

A) Full view:

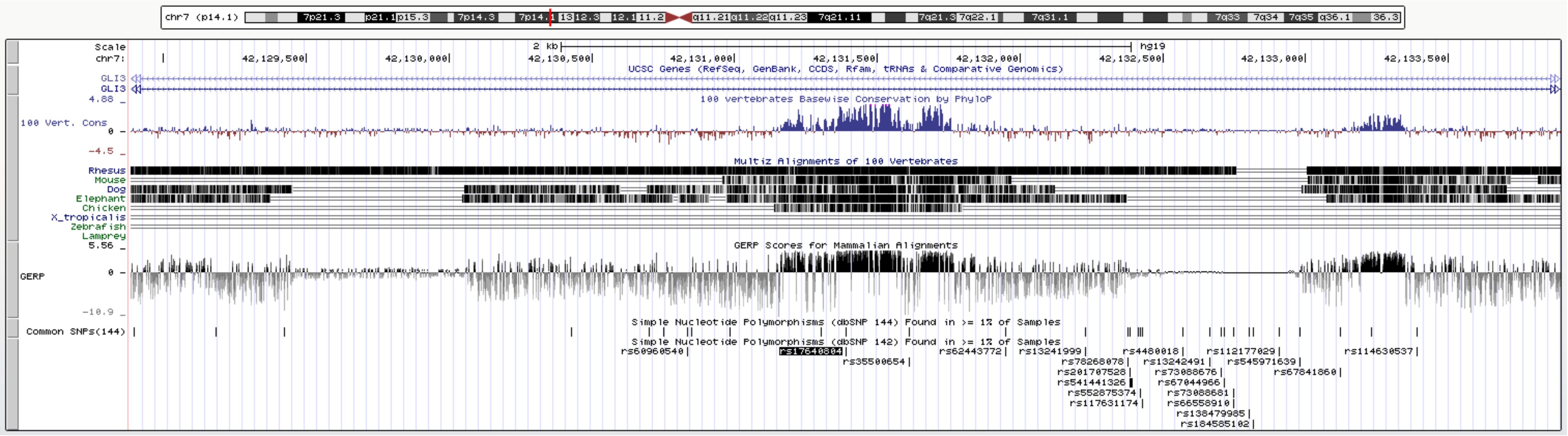

B) Cropped view:

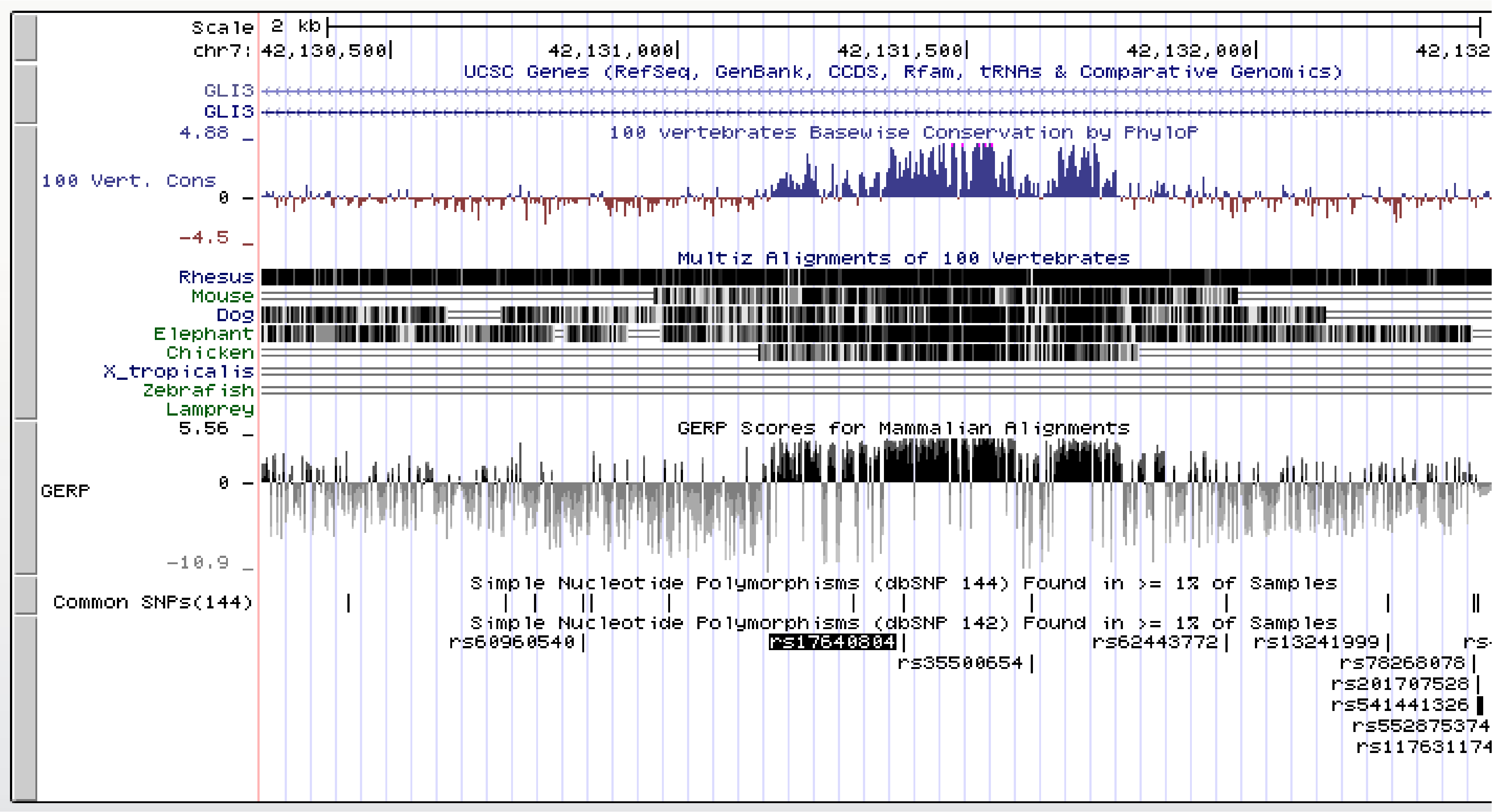

Supplementary Figure 14: SNP quality control and Hardy-Weinberg equilibrium

Hardy-Weinberg Equilibrium (HWE) is often used in GWAS studies as "indicative of a genotyping or genotype-calling error" [10], but deviations from HWE can be caused by other population-level factors such as admixture, substructure, inbreeding or selection [10-14]. When genotyping quality is high, rejection of GWAS SNPs based on HWE can remove real signals [11-13], particularly when, as here, selection at loci associated with our phenotypes is plausible, such as the case for the EDAR functional variant rs3827760 [15-16].

In addition to selection causing deviation from HWE, admixture between three continental ancestries is one of the major reasons for studying our Candela population. This choice brings helpful genetic diversity, and the effects of ancestry are controlled for in our analysis and so cause little problem. Admixed populations are expected to display more deviation from HWE than a genetically homogeneous population, but this is not a cause for concern.

In such situations some have suggested using a more lenient threshold for the HWE *P* value [10, 17], though it has been recommended that manually checking cluster plots produced by the genotype calling software is the preferred way to check for SNP quality [10], which was done for each reported index SNP. We therefore chose not to test for HWE as a check on genotyping errors; instead we perform biological and software-level quality controls (QC) to ensure genotyping quality, which we describe below.

A) Genome-wide deviation from HWE in Latin American samples:

To show that our set of post-QC SNPs show deviation from HWE not as a result of genotyping error but due to population substructure, we performed HWE test on the same set of SNPs for Latin American samples from the 1000 Genomes Phase 3 release, which contains genotypes ascertained from sequencing data and commonly used as a reference. 328 Latin American individuals from this release (after removing related individuals) were used for the HWE *P* value calculation in Plink 1.9. The Q-Q plot of HWE *P* value is shown below:

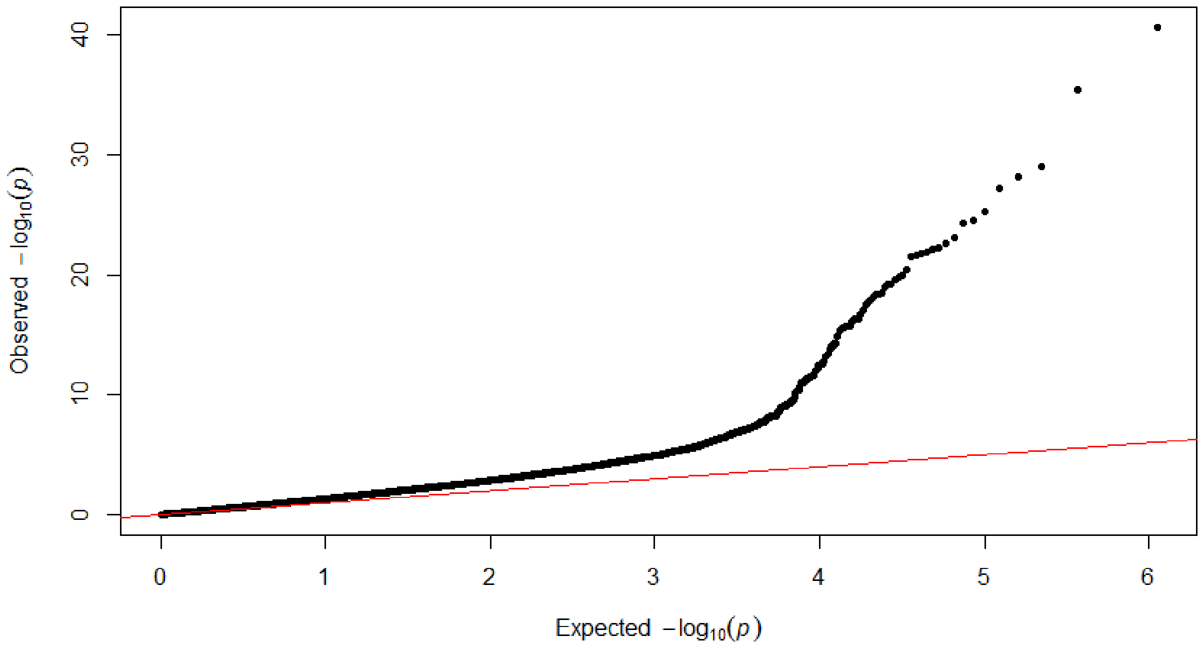

Since *P* values depend to a large extent on sample size, a random subset of Candela individuals were selected separately from each country to match the 1000 Genomes Phase 3 composition. Q-Q plot for HWE *P* value for the same set of SNPs in this set of 328 Candela individuals are shown below:

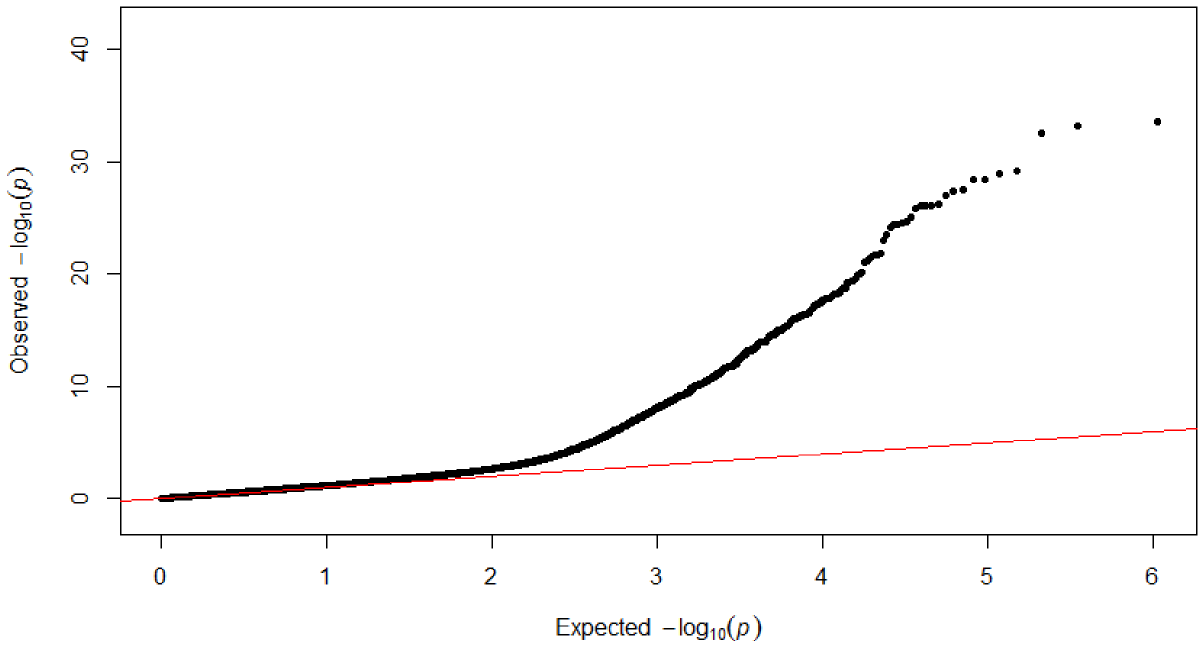

The Q-Q plots show that similar levels of HWE *P* value inflation in Latin Americans are present in both datasets, despite being genotyped in very different ways. Provided that *P* value decreases with increased sample size when effect size remains constant, it is expected that the total Candela sample which has nearly 20 times more samples than this example dataset would have even higher levels of deviation of HWE *P* value. Consider rs3827760 as an example: its F-statistic i.e. 1 – (observed % of heterozygous / expected % of heterozygous) for the 1000 Genomes data of 328 individuals is 0.2455, with a *P* value of 1E-05. With high-coverage exome sequencing in 1000 Genomes, the genotypes for this SNP can be considered reliable. Given that a chi-square statistic increases with sample size, the same frequency distribution of genotypes (giving the same F-statistic) in 6357 individuals will approximately produce a *P* value of 3E-83. In comparison, the HWE *P* value for this SNP in the random subset of 328 Candela individuals is 7E-4 and the whole Candela dataset is 8E-52, at comparable levels with the 1000 Genomes data. The Genotype-calling cluster plots produced by Illumina Genomestudio software for Candela data for rs3827760 is shown below, as an indication that the genotyping calling is of acceptable quality. Each dot represents a sample, the three colours indicate three genotype categories. Black dots indicates samples with uncalled genotypes, in this case it is the blank (water) sample included as a null control. Such manual checks were performed for each index SNP.

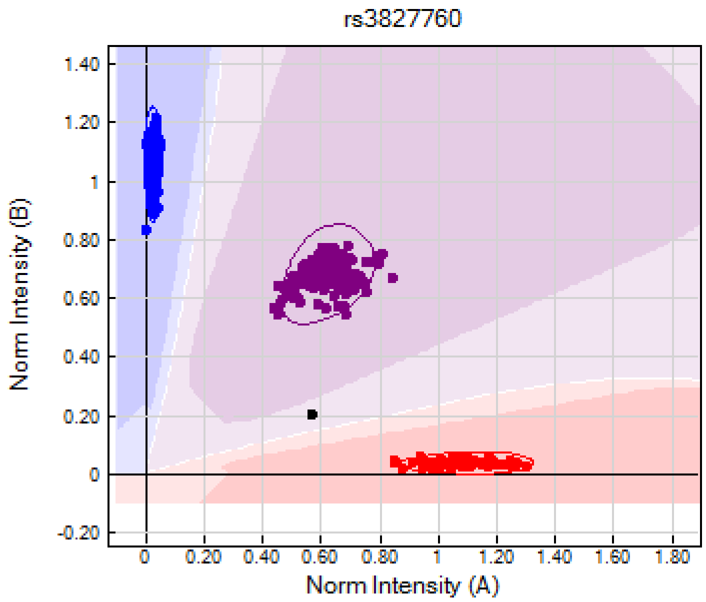

B) Quality control for software genotype calls:

Following recommended protocol of quality control on the Illumina GenomeStudio genotype calls [18–19], the SNP quality metrics generated from the GenCall algorithm in GenomeStudio were investigated. SNPs with low GenTrain score (<0.7), low Cluster Separation score (<0.3) or high heterozygosity values (|het. excess|>0.5) were excluded [18-23]. The heterozygosity excess filter performs a function similar to a HWE check, but is more direct since it is based on the heterozygosity value, which unlike the *P* value doesn't depend on sample size (see part A). Only SNPs that satisfy these criteria across all genotyping plates were retained.

C) Biological quality control:

In each genotyping plate preparation a randomly selected previously genotyped sample is included as a control sample (positioned randomly on the plate) to check for genotyping consistency. Genotyping calls across different plates are compared for each control sample. The consistency rate (i.e. matched proportion of genotypes) was ≥0.9999 in all cases after SNP-level QC. A set of control samples was re-genotyped on different plates after 2x and 4x dilutions or concentrations, and genotyping consistency was checked. In each case the consistency was ≥0.9996.

SUPPLEMENTARY TABLES

Supplementary Table 1: Features of the study sample

	Total	Colombia	Brazil	Chile	Mexico	Peru
**Sample size**	6275	1402	658	1760	1200	1255
**Percentage**	100	22.3	10.5	28.1	19.1	20
**% Female**	54.1	56.3	68.9	39.4	60.3	58.5
**Age (years)**						
Min	18	18	18	18	18	18
Mean	24.2	24	25.8	25.2	24.4	22.2
Max	45	40	45	45	44	44
S.D.	5.7	5.3	6.3	5.8	5.6	5.2
**Age, for Males**						
Min	18	18	18	18	18	18
Mean	24.9	24.7	25.8	25.3	25.1	23
Max	45	40	45	45	44	44
S.D.	5.7	5.5	6.4	5.5	5.6	5.7
**Age, for Females**						
Min	18	18	18	18	18	18
Mean	23.8	23.5	25.4	25.2	24	21.6
Max	45	40	44	45	41	42
S.D.	5.7	5	4.2	6.2	4.7	4.7

Supplementary Table 2: Ordinal face traits examined

Face section	Trait	Categories		
		0	1	2
Upper	Forehead profile	Steep (vertical)	Sloping	-
	Brow ridge protrusion	None	Slightly pronounced	Strongly pronounced
Middle	Cheekbone protrusion	None	Slightly pronounced	Strongly pronounced
	Nasal root breadth	Narrow	Broad	
	Nose bridge breadth	Short	Average	Long
	Nose wing breadth	Narrow	Average	Broad
	Nose profile	Convex	Straight	Concave
	Nose protrusion	Low	Slightly	Strong
	Nose tip shape	Pointed	Round	Bulbous
	Columella inclination	Up	Straight	Down
Lower	Upper lip thickness	Thin	Average	Full
	Lower lip thickness	Thin	Average	Full
	Chin shape	Pointed	Round	Square
	Chin protrusion	Receding	Normal	Pronounced

Supplementary Table 3: Rater reliability for face trait scores

We evaluated the rater reliability for face traits scores by calculating intra-class correlation coefficients (ICC) following the definition of Shrout & Fleiss [24]. This approach uses a two-way mixed effects ANOVA model, with two different scorings (from repeated scoring by one rater or from scores by two raters) as a fixed effect, and variation across subjects as a random effect. Scores from a set of photographs for 450 individuals (>7% of total sample size, combining all 5 constituent countries) were used for calculating ICCs for each facial trait. The photographs were scored twice by two raters, independently, two weeks apart.

Face section	Trait	Rater 1 (M.F.G.)	Rater 2 (I.P.A.)	Inter-rater
Upper	Brow ridge protrusion	0.77	0.78	0.69
	Forehead profile	0.76	0.67	0.48
Middle	Cheekbone protrusion	0.71	0.50	0.44
	Nasal root breadth	0.77	0.46	0.42
	Nose bridge breadth	0.61	0.58	0.48
	Nose wing breadth	0.68	0.72	0.65
	Nose profile	0.66	0.73	0.65
	Nose protrusion	0.75	0.63	0.53
	Nose tip shape	0.80	0.63	0.51
	Columella inclination	0.72	0.81	0.57
Lower	Upper lip thickness	0.80	0.85	0.68
	Lower lip thickness	0.75	0.66	0.69
	Chin shape	0.76	0.66	0.65
	Chin protrusion	0.75	0.59	0.57

Supplementary Table 4: Correlations of ordinal face traits

A) Correlations between face traits:

Pearson correlation coefficients (r) are presented in the lower left triangle (color-coded based on magnitude of correlation), with corresponding permutation *P* values in the upper right triangle. Correlations with significant *P* values (<0.0006, Bonferroni-adjusted threshold) and their corresponding *P* values are highlighted in bold. *P* values were obtained from 2000 permutations. If the observed r value was less than all permuted r values then that *P* value was taken to be <0.0005.

Face section	Trait	FP	BR	CB	NR	NB	NW	NP	NPR	NT	CI	ULT	LLT	CS	CP
Upper	Forehead profile		**<5E-4**	1E-02	**<5E-4**	**<5E-4**	**<5E-4**	2E-02	**<5E-4**	**<5E-4**	**<5E-4**	2E-01	9E-01	**<5E-4**	4E-01
	Brow ridge protrusion	**0.57**		**<5E-4**	**<5E-4**	**<5E-4**	**<5E-4**	**<5E-4**	2E-03	3E-01	**<5E-4**	7E-02	6E-01	**<5E-4**	4E-01
Middle	Cheekbone protrusion	-0.03	**-0.10**		2E-02	**<5E-4**	**<5E-4**	**<5E-4**	1E-02	5E-01	**<5E-4**	6E-02	4E-01	3E-02	**<5E-4**
	Nasal root breadth	**-0.10**	**-0.08**	0.03		**<5E-4**	**<5E-4**	8E-03	**<5E-4**	**<5E-4**	**<5E-4**	**<5E-4**	**<5E-4**	3E-01	7E-03
	Nose bridge breadth	**0.12**	**0.18**	**-0.05**	**0.29**		**<5E-4**	3E-02	**<5E-4**	**<5E-4**	**<5E-4**	**<5E-4**	**<5E-4**	4E-02	1E-01
	Nose wing breadth	**0.10**	**0.17**	**-0.05**	**0.16**	**0.37**		2E-02	**<5E-4**	**<5E-4**	**<5E-4**	**<5E-4**	**<5E-4**	3E-03	7E-03
	Nose profile	-0.03	**-0.05**	**0.05**	0.03	-0.03	-0.03		9E-03	2E-01	**<5E-4**	6E-01	5E-01	9E-01	1E-01
	Nose protrusion	**0.11**	0.04	0.03	**-0.16**	**-0.08**	**-0.14**	0.03		**<5E-4**	**<5E-4**	**<5E-4**	**<5E-4**	7E-01	1E-01
	Nose tip shape	**-0.05**	0.01	0.01	**0.18**	**0.16**	**0.29**	0.02	**-0.25**		**<5E-4**	**<5E-4**	**<5E-4**	4E-01	1E+00
	Columella inclination	**0.08**	**0.11**	**-0.09**	**-0.06**	**0.05**	**0.05**	**-0.20**	**0.10**	**-0.14**		8E-04	**<5E-4**	2E-01	5E-02
Lower	Upper lip thickness	0.02	0.02	-0.02	**0.07**	**0.08**	**0.11**	-0.01	**-0.21**	**0.12**	-0.04		**<5E-4**	3E-01	**<5E-4**
	Lower lip thickness	0.00	-0.01	-0.01	**0.05**	**0.06**	**0.06**	0.01	**-0.13**	**0.08**	**-0.06**	**0.72**		5E-01	**<5E-4**
	Chin shape	**0.06**	**0.08**	0.03	-0.01	0.03	0.04	0.00	0.00	0.01	-0.02	0.01	-0.01		2E-03
	Chin protrusion	-0.01	-0.01	**0.05**	0.03	0.02	0.03	0.02	0.02	0.00	-0.02	**-0.13**	**-0.17**	0.04	

B) Correlation between face traits and covariates:

	Upper		Middle								Lower			
Covariate	FP	BR	CB	NR	NB	NW	NP	NPR	NT	CI	ULT	LLT	CS	CP
Sex	**-0.47**	**-0.62**	**0.24**	**0.16**	**-0.23**	**-0.17**	**0.10**	**-0.05**	**0.05**	**-0.20**	-0.01	0.02	**-0.08**	**0.08**
Age	0.09	**0.06**	0.02	-0.04	-0.01	-0.02	-0.01	**0.08**	**-0.06**	**0.08**	**-0.19**	**-0.24**	0.04	**0.06**
BMI	**0.11**	**0.17**	**-0.16**	**0.07**	**0.10**	**0.10**	-0.02	**-0.15**	**0.11**	0.04	-0.01	**-0.08**	**-0.08**	**0.05**
African anc.	-0.02	-0.01	0.02	**0.06**	**0.06**	**0.06**	0.02	-0.03	0.03	0.00	0.02	**0.06**	0.03	0.03
European anc.	0.02	0.02	0.04	**-0.08**	**-0.05**	**-0.15**	**0.05**	**0.18**	**-0.11**	**-0.06**	**-0.25**	**-0.16**	0.01	**0.12**
American anc.	-0.01	-0.02	-0.04	**0.06**	0.03	**0.13**	**-0.05**	**-0.16**	**0.09**	**0.06**	**0.23**	**0.13**	-0.02	**-0.13**

Corresponding *P* values:

	Upper		Middle								Lower			
Covariate	BR	FP	CB	NR	NB	NW	NP	NPR	NT	CI	ULT	LLT	CS	CP
Sex	**<5E-4**	**<5E-4**	**<5E-4**	**<5E-4**	**<5E-4**	**<5E-4**	**<5E-4**	**<5E-4**	**<5E-4**	**<5E-4**	5E-01	6E-02	**<5E-4**	**<5E-4**
Age	**<5E-4**	**<5E-4**	1E-01	6E-01	3E-03	2E-01	4E-01	**<5E-4**	**<5E-4**	**<5E-4**	**<5E-4**	**<5E-4**	**<5E-4**	7E-04
BMI	**<5E-4**	**<5E-4**	**<5E-4**	**<5E-4**	**<5E-4**	**<5E-4**	2E-01	**<5E-4**	**<5E-4**	5E-04	4E-01	**<5E-4**	**<5E-4**	**<5E-4**
African anc.	4E-01	8E-02	1E-01	**<5E-4**	5E-02	**<5E-4**	6E-02	8E-03	2E-02	9E-01	2E-01	9E-06	5E-02	**<5E-4**
European anc.	7E-02	2E-01	6E-03	**<5E-4**	4E-01	**<5E-4**	**<5E-4**	**<5E-4**	**<5E-4**	**<5E-4**	**<5E-4**	**<5E-4**	**<5E-4**	**<5E-4**
American anc.	2E-01	4E-01	2E-03	2E-02	1E-01	**<5E-4**	**<5E-4**	**<5E-4**	**<5E-4**	**<5E-4**	**<5E-4**	**<5E-4**	**<5E-4**	**<5E-4**

anc. = Continental ancestry estimated from the genetic data (Supplementary Fig. 3).

Sex coded as female=1, male=0.

Correlations with significant *P* values (<0.0005, Bonferroni-adjusted threshold), obtained by permutation, are highlighted in bold.

Supplementary Table 5: Heritability estimates for the 14 ordinal face traits examined

**Face section**	**Trait**	**Heritability**	**S.E.**	***P* value**
Upper	Forehead profile	0.28	0.06	2.6E-14
	Brow ridge protrusion	0.44	0.06	0.0E+00
Middle	Cheekbone protrusion	0.28	0.05	1.8E-15
	Nasal root breadth	0.23	0.05	2.2E-16
	Nose bridge breadth	0.23	0.06	8.5E-13
	Nose wing breadth	0.41	0.05	0.0E+00
	Nose profile	0.22	0.06	4.8E-10
	Nose protrusion	0.47	0.05	0.0E+00
	Nose tip shape	0.27	0.05	0.0E+00
	Columella inclination	0.20	0.06	6.9E-12
Lower	Upper lip thickness	0.46	0.06	0.0E+00
	Lower lip thickness	0.40	0.06	0.0E+00
	Chin shape	0.31	0.06	3.1E-14
	Chin protrusion	0.22	0.05	0.0E+00

Supplementary Table 6: False Discovery Rate multiple testing correction

We applied the False Discovery Rate (FDR) method of multiple testing correction [25] to the combined set of all 14 categorical trait GWAS *P* values. The FDR method controls the overall Type I error rate (false rejection of true null hypotheses) at a specified level 0.05, while not being as overly conservative as the Bonferroni correction method. The Benjamini–Hochberg procedure for FDR was applied, giving a significance threshold of 9.05E-08 for all tests. Using this procedure, 17 SNPs from 4 regions were significant, corresponding to the same reported associated regions in Table 1. The FDR results are presented below, with the four index SNPs from Table 1 highlighted in bold.

Rank	Chromosome	SNP	Trait	*P* value
1	**6**	**rs1852985**	**Nose Bridge Breadth**	**6.15E-10**
2	**20**	**rs927833**	**Nose Wing Breadth**	**1.05E-09**
3	6	rs1285029	Nose Bridge Breadth	5.80E-09
4	**4**	**rs12644248**	**Columella Inclination**	**6.64E-09**
5	6	rs6458435	Nose Bridge Breadth	6.79E-09
6	6	rs6458432	Nose Bridge Breadth	7.14E-09
7	**7**	**rs17640804**	**Nose Wing Breadth**	**8.85E-09**
8	6	rs1284964	Nose Bridge Breadth	9.40E-09
9	4	rs10517589	Columella Inclination	1.25E-08
10	6	rs12529907	Nose Bridge Breadth	1.59E-08
11	7	rs846312	Nose Wing Breadth	1.70E-08
12	4	rs10029359	Columella Inclination	2.40E-08
13	4	rs12651681	Columella Inclination	2.47E-08
14	6	rs35565233	Nose Bridge Breadth	2.73E-08
15	4	rs12506449	Columella Inclination	6.57E-08
16	4	rs6821649	Columella Inclination	7.90E-08
17	6	rs12528232	Nose Bridge Breadth	8.71E-08

Supplementary Table 7: Meta-analysis *P* values for index SNPs

For each index SNP in Table 1 a GWAS was performed independently in each of the five country samples (including calculation of genetic PCs separately for each country). The GWAS *P* values were then combined as a meta-analysis. Cochran's Q statistic was computed in each case to test effect size heterogeneity. It was non-significant in all cases.

A) Overall meta-analysis *P* value:

**Region**	**SNP**	**Allele**	**Trait**	***P* value**	**Beta**	**S.E.**	**Q Stat. *P* value**
4q31	rs12644248	G	Columella Inclination	1.92E-07	-0.0741	0.0142	0.3139
6p21	rs1852985	T	Nose Bridge Breadth	5.64E-10	0.0658	0.0110	0.1238
7p13	rs17640804	C	Nose Wing Breadth	3.69E-09	0.0612	0.0104	0.9355
20p11	rs927833	T	Nose Wing Breadth	5.84E-12	0.0831	0.0121	0.7114

B) Country-wise breakdown of *P* values:

**SNP**	**Trait**	**Composite *P* value (n=5958)**	**Colombia (n=1303)**	**Brazil (n=608)**	**Chile (n=1651)**	**Mexico (n=1165)**	**Peru (n=1231)**
rs12644248	Columella Inclination	1.92E-07	6.16E-01	1.52E-01	1.64E-03	5.87E-02	6.66E-05
rs1852985	Nose Bridge Breadth	5.64E-10	2.55E-03	3.78E-01	3.63E-06	2.26E-03	1.40E-02
rs17640804	Nose Wing Breadth	3.69E-09	1.69E-02	7.22E-02	8.62E-04	3.39E-03	8.88E-03
rs927833	Nose Wing Breadth	5.84E-12	8.96E-05	1.86E-01	5.52E-03	6.39E-04	3.50E-04

Supplementary Table 8: Multivariate association analysis combining all traits

Multivariate regression analysis, an extension of the single-trait regression performed in GWAS, was performed to check if correlations between traits can lead to new or stronger genetic signals. The regression analysis was performed for each SNP to test for association with all traits combined while adjusting for covariates age, sex, BMI and genetic PCs. The method provides a regression coefficient for each trait, and the vector of coefficients is tested jointly using the Wald test [26] to check if it deviates significantly from a zero vector. The test employed here is nearly identical to the Wald test performed in the multivariate linear regression model analysis used in Zhou and Stephens (2014) [27], the only difference being in the use of genetic PCs instead of genetic kinship matrix; the difference is minor since the PCs are top eigenvectors of the kinship matrix.

P values from this test that exceed the suggestive significance threshold of 10^{-5} are reported below, with the four index SNPs from Table 1 highlighted in bold. No new gene regions were found to be significantly associated. This is expected in cases when the correlation between traits is relatively low and effects of SNPs are not shared across phenotypes [28].

Chromosome	SNP	Position	Nearest gene	Wald *P* value	$-\log_{10}(P)$
4	rs9995821	154828366	DCHS2	1.97E-07	6.70
4	**rs12644248**	155235392	DCHS2	5.42E-06	5.27
6	rs542444	44800015	SUPT3H	1.80E-08	7.75
6	rs12528232	44982593	SUPT3H	1.56E-08	7.81
6	rs1285007	45102025	SUPT3H	5.34E-09	8.27
6	rs1285029	45122247	SUPT3H	8.87E-09	8.05
6	rs1284964	45176540	SUPT3H	9.39E-09	8.03
6	rs6458432	45205017	SUPT3H	5.98E-09	8.22
6	rs6458435	45222674	SUPT3H	5.96E-09	8.22
6	rs12529907	45255230	SUPT3H	1.39E-08	7.86
6	**rs1852985**	45329656	SUPT3H| RUNX2	8.65E-10	9.06
6	rs35565233	45349497	RUNX2	2.76E-08	7.56
7	rs846312	42128896	GLI3	2.88E-07	6.54
7	**rs17640804**	42131390	GLI3	2.54E-07	6.59
20	**rs927833**	22041577	PAX1	1.77E-08	7.75

Supplementary Table 9: Measurements defined using 3D facial landmarks

Face section	Trait	Quantitative definition[a,b]
Upper	Forehead profile	–
	Brow ridge protrusion	–
Middle	Nasion position	Distance from landmark 18 to the mid-point of a line joining landmarks 8 and 16
	Cheekbone protrusion	–
	Nasal root breadth	–
	Nose bridge breadth	–
	Nose wing breadth	Distance between landmarks 20 and 22
	Nose profile	–
	Nose protrusion	Distance of landmark 19 to a line joining landmarks 18 and 21
	Nose tip shape	Angle between landmarks 18-19-21
	Columella inclination	Angle between landmarks 19-21-23
Lower	Upper lip thickness	Distance between landmarks 23 and 26
	Lower lip thickness	Distance between landmarks 26 and 29
	Chin shape	–
	Chin protrusion	Ratio of distance between landmarks 30-32 to distance between landmarks 29-32

[a] The quantitative definitions refer to coordinate numbers as shown in Supplementary Figure 5A.

[b] A dash (–) indicates that a measurement corresponding to this ordinal phenotype could not be defined based on the 3-D landmarks.

Supplementary Table 10: Measurements defined using 2-D facial landmarks

Face section	Trait	Quantitative definition[a]
Middle	Nasal root breadth	Distance between landmarks 3 and 4
	Nose bridge breadth	Distance between landmarks 5 and 6

[a] The quantitative definitions refer to coordinate numbers as shown in Supplementary Figure 5B.

Supplementary Table 11: Correlations between categorical and quantitative face traits

Correlations were calculated between categorical and their corresponding quantitative face traits as defined in Supplementary Tables 9 and 10. *P* values were obtained by performing 2000 permutations. If the observed r value was less than all permuted r values then the *P* value was taken to be <0.0005. Boxplots for quantitative vs. categorical traits are presented in Supplementary Figure 6.

Trait	Correlation	*P* value
Nasal root breadth	0.48	<0.0005
Nose bridge breadth	0.37	<0.0005
Nose wing breadth	0.70	<0.0005
Nose protrusion	0.58	<0.0005
Nose tip angle	0.17	<0.0005
Columella inclination	-0.16[a]	<0.0005
Upper lip thickness	0.60	<0.0005
Lower lip thickness	0.70	<0.0005
Chin protrusion	0.58	<0.0005

[a] Correlation for Columella inclination is expected to be negative. Since the categorical trait is coded as 0-1-2 = up-straight-down, which corresponds to the columella angle being wide-moderate-narrow, hence it is negatively correlated with angle magnitude.

Supplementary Table 12: Correlations for quantitative face traits

A) Correlations between traits:

Correlation values are presented in the lower left triangle, with corresponding permutation *P* values in the upper right triangle. Correlations with significant *P* values (<0.0006, Bonferroni-adjusted threshold) and their corresponding *P* values are highlighted in bold.

	NRB	NBB	NWB	NPR	NTA	CI	ULT	LLT	CP	NAP
Nasal root breadth		**<0.0005**	**<0.0005**	**<0.0005**	**<0.0005**	0.008	**<0.0005**	**<0.0005**	0.729	**<0.0005**
Nose bridge breadth	**0.568**		<0.0005	0.005	0.002	**<0.0005**	**<0.0005**	**<0.0005**	0.02	**<0.0005**
Nose wing breadth	**0.213**	0.389		0.011	**<0.0005**	**<0.0005**	0.034	0.053	0.005	**<0.0005**
Nose protrusion	**-0.179**	-0.052	-0.047		**<0.0005**	0.053	**<0.0005**	**<0.0005**	**<0.0005**	**<0.0005**
Nose tip angle	**0.198**	0.057	**-0.068**	**-0.635**		**<0.0005**	**<0.0005**	**<0.0005**	**<0.0005**	**<0.0005**
Columella inclination	-0.049	**-0.111**	**-0.217**	-0.036	**0.443**		**<0.0005**	**<0.0005**	0.008	**<0.0005**
Upper lip thickness	**0.161**	**0.122**	0.039	**-0.115**	**0.119**	**-0.272**		**<0.0005**	**<0.0005**	**<0.0005**
Lower lip thickness	**0.121**	**0.073**	-0.036	**-0.075**	**0.152**	**-0.097**	**0.573**		**<0.0005**	0.001
Chin protrusion	-0.006	0.043	0.052	**0.081**	**-0.126**	0.049	**-0.366**	**-0.499**		**<0.0005**
Nasion position	**-0.17**	**-0.094**	**-0.183**	**0.292**	**-0.148**	**0.138**	**-0.151**	-0.064	**0.093**	

B) Correlation between quantitative face traits and covariates:

	Middle							Lower		
Covariate	NAP	NRB	NBB	NWB	NPR	NTA	CI	ULT	LLT	CP
Sex	**-0.35**	**0.28**	0.06	**-0.11**	**-0.06**	**0.16**	0.00	**0.13**	**0.13**	**-0.08**
Age	0.01	**-0.12**	-0.02	**0.16**	**0.26**	**-0.27**	0.00	**-0.43**	**-0.47**	**0.21**
BMI	**-0.07**	-0.04	-0.02	**0.15**	**-0.06**	**-0.09**	-0.05	**-0.20**	**-0.23**	**0.23**
African anc.	**-0.06**	**0.23**	**0.25**	**0.19**	-0.03	0.06	-0.05	**0.17**	**0.19**	**-0.07**
European Anc.	**0.38**	**-0.12**	**-0.14**	**-0.24**	**0.36**	**-0.19**	**0.19**	**-0.22**	**-0.07**	**0.20**
American anc.	**-0.32**	0.05	**0.07**	**0.17**	**-0.33**	**0.16**	**-0.16**	**0.15**	0.01	**-0.17**

Corresponding *P* values:

	Middle							Lower		
Covariate	NAP	NRB	NBB	NWB	NPR	NTA	CI	ULT	LLT	CP
Sex	**<5E-4**	**<5E-4**	2.E-03	**<5E-4**	**<5E-4**	**<5E-4**	9.E-01	**<5E-4**	**<5E-4**	**<5E-4**
Age	6.E-01	**<5E-4**	2.E-01	**<5E-4**	**<5E-4**	**<5E-4**	9.E-01	**<5E-4**	**<5E-4**	**<5E-4**
BMI	**<5E-4**	4.E-02	4.E-01	**<5E-4**	**<5E-4**	**<5E-4**	2.E-03	**<5E-4**	**<5E-4**	**<5E-4**
African anc.	**<5E-4**	**<5E-4**	**<5E-4**	**<5E-4**	8.E-02	7.E-04	9.E-03	**<5E-4**	**<5E-4**	**<5E-4**
European Anc.	**<5E-4**	**<5E-4**	**<5E-4**	**<5E-4**	**<5E-4**	**<5E-4**	**<5E-4**	**<5E-4**	**<5E-4**	**<5E-4**
American anc.	**<5E-4**	4.E-03	**<5E-4**	**<5E-4**	**<5E-4**	**<5E-4**	**<5E-4**	**<5E-4**	6.E-01	**<5E-4**

anc. = Continental ancestry estimated from the genetic data.

Sex coded as female=1, male=0.

Correlations with significant *P* values (<0.0006, Bonferroni-adjusted threshold) are highlighted in bold.

Supplementary Table 13: Heritability of quantitative face traits

**Face region**	**Trait**	**Heritability**	**S.E.**	***P* value**
Mid-face	Nasion Position	0.80	0.10	0.0E+00
	Nasal root breadth	0.80	0.09	0.0E+00
	Nose bridge breadth	0.89	0.09	0.0E+00
	Nose wing breadth	0.90	0.10	0.0E+00
	Nose protrusion	0.84	0.10	0.0E+00
	Nose tip shape	0.67	0.10	0.0E+00
	Columella inclination	0.77	0.11	0.0E+00
Lower face	Upper lip thickness	0.63	0.10	0.0E+00
	Lower lip thickness	0.46	0.10	2.3E-15
	Chin protrusion	0.45	0.09	0.0E+00

Supplementary Table 14: Fraction of trait variance explained by the index SNPs of Tables 1 and 2.

For all traits we calculated the fraction of trait variance explained by the covariates and by all index SNPs. A similar regression model to that used in the GWAS was used to estimate the R^2 of the model (representing the fraction of the variance of the trait explained by all regressors). The following models were applied:

Model 1: Trait ~ age + sex + BMI + PC1 … PC5

Model 2: Trait ~ age + sex + BMI + PC1 … PC5 + index SNP

Model 3: Trait ~ age + sex + BMI + PC1 … PC5 + all index SNPs

Model 2 was applied separately for each index SNP.

From models 2 and 3, the fraction of trait variance explained by the covariates (i.e. Model 1) was subtracted to get the additional contribution of the SNP(s).

SNPs showing genome-wide significant associations to a trait are highlighted in bold.

A) Ordinal traits:

	R^2 explained by (%)								
Trait	Covariates	rs3827760	rs7559271	rs2045323	rs12644248	rs1852985	rs17640804	rs927833	All SNPs
Forehead profile	23.60	0.00	0.01	0.14	0.12	0.02	0.05	0.04	0.29
Brow ridge protrusion	39.81	0.01	0.02	0.02	0.22	0.24	0.00	0.00	0.46
Cheekbone protrusion	8.75	0.03	0.00	0.01	0.01	0.02	0.08	0.02	0.09
Nasal root breadth	5.84	0.07	0.17	0.02	0.02	0.21	0.02	0.00	0.45
**Nose bridge breadth**	7.08	0.01	0.01	0.00	0.09	**0.71**	0.06	0.01	0.92
**Nose wing breadth**	9.61	0.00	0.08	0.01	0.14	0.01	**0.62**	**0.66**	1.53
Nose profile	4.77	0.00	0.01	0.14	0.11	0.02	0.04	0.00	0.26
Nose protrusion	1.93	0.00	0.10	0.01	0.06	0.03	0.00	0.05	0.27
Nose tip shape	11.57	0.03	0.02	0.01	0.00	0.01	0.01	0.03	0.11
**Columella inclination**	5.29	0.00	0.07	0.44	**0.49**	0.09	0.03	0.00	0.86
Upper lip thickness	12.09	0.02	0.14	0.09	0.22	0.01	0.01	0.03	0.51
Lower lip thickness	9.61	0.08	0.09	0.01	0.19	0.04	0.00	0.01	0.41
Chin shape	2.77	0.00	0.03	0.03	0.09	0.02	0.01	0.03	0.14
Chin protrusion	4.83	0.15	0.01	0.03	0.03	0.02	0.03	0.09	0.36

B) Quantitative traits:

	R^2 explained by (%)								
Trait	Covariates	rs3827760	rs7559271	rs2045323	rs12644248	rs1852985	rs17640804	rs927833	All SNPs
Nasal root breadth	19.31	0.16	0.03	0.14	0.39	0.17	0.01	0.12	0.94
**Nose bridge breadth**	13.84	0.00	0.10	0.09	0.15	**1.18**	0.26	0.24	1.80
**Nose wing breadth**	15.27	0.03	0.13	0.05	0.17	0.10	**1.15**	**0.57**	2.28
**Nose protrusion**	17.72	0.00	0.28	**0.95**	0.29	0.01	0.01	0.07	1.32
**Nose tip angle**	10.15	0.01	0.07	**1.08**	0.52	0.05	0.02	0.02	1.37
**Columella inclination**	6.06	0.01	0.14	**0.63**	**0.44**	0.13	0.00	0.01	1.13
Upper lip thickness	16.75	0.09	0.10	0.09	0.39	0.08	0.02	0.00	0.32
Lower lip thickness	13.66	0.22	0.06	0.18	0.22	0.07	0.00	0.07	0.35
**Chin protrusion**	11.35	**1.32**	0.11	0.12	0.07	0.09	0.03	0.02	1.09
**Nasion position**	30.21	0.01	**1.33**	0.22	0.15	0.09	0.01	0.04	1.15

Supplementary Table 15: Testing for association of NSCL/P loci with the facial traits examined here

A) SNP-level tests:

We selected index SNPs from the 15 regions reported to be associated with NSCL/P (the selected SNPs were chosen from references [29-37]) and tested their association with the face traits examined here. For each SNP the smallest *P* value across traits is presented below, in addition to the Bonferroni-corrected (adjusted by the number of traits) *P* value. In each case we also indicate the trait with strongest association.

<u>Ordinal traits</u>

Region	Chromosome	Gene	SNP	Strongest associated trait	min P	min P (adjusted)
1	1p36.13	PAX7	rs560426[1,3]	Nasal root breadth	0.0275	0.3846
2	1p22.1	ABCA4	rs742071[1,2]	Nose wing breadth	0.0285	0.3983
3	1q32.2	IRF6	rs2235371[4,6]	Brow ridge protrusion	0.0019	0.0269
4	2p21	THADA	rs7590268[1,7]	Nose protrusion	0.0744	1.0000
5	3p11.1	EPHA3	rs7632427[1]	Nose protrusion	0.0342	0.4792
6	3p12.3	COL8A1/FILIP1L	rs793464[2]	Nose wing breadth	0.0281	0.3933
7	8q21.3	DCAF4L2	rs12543318[1]	Brow ridge protrusion	0.0287	0.4012
8	8q24.21	--	rs987525[1,3,8,9]	Nose protrusion	0.0727	1.0000
9	10q25.3	VAX1	rs7078160[1,6,7]	Nose bridge breadth	0.0316	0.4425
10	13q31.1	SPRY2	rs8001641[1]	Nasal root breadth	0.0361	0.5051
11	15q22	TMP1	rs1873147[1]	Chin shape	0.0416	0.5817
12	16p13.3	ADCY9	rs8049367[6]	Nose wing breadth	0.0122	0.1708
13	17p13.1	NTN1	rs479177[6]	Nose protrusion	0.0207	0.2901
14	17q22	NOG	rs227731[1,7]	Chin protrusion	0.0131	0.1838
15	20q12	MAFB	rs13041247[1,3,6]	Nasal root breadth	0.0335	0.4693

Quantitative traits

Region	Choromosome	Gene	SNP	Trait	min P	min P (adjusted)
1	1p36.13	PAX7	rs560426[1,3]	Nose wing breadth	0.00082	0.00816
2	1p22.1	ABCA4	rs742071[1,2]	Nasion position	0.08715	0.87150
3	1q32.2	IRF6	rs2235371[4,6]	Upper lip thickness	0.00661	0.06612
4	2p21	THADA	rs7590268[1,7]	Nose bridge breadth	0.02832	0.28320
5	3p11.1	EPHA3	rs7632427[1]	Chin protrusion	0.10080	1.00000
6	3p12.3	COL8A1/FILIP1L	rs793464[2]	Lower lip thickness	0.02898	0.28980
7	8q21.3	DCAF4L2	rs12543318[1]	Columella inclination	0.01585	0.15850
8	8q24.21	--	rs987525[1,3,8,9]	Nose wing breadth	0.05817	0.58170
9	10q25.3	VAX1	rs7078160[1,6,7]	Nasal root breadth	0.24310	1.00000
10	13q31.1	SPRY2	rs8001641[1]	Nasion position	0.23940	1.00000
11	15q22	TMP1	rs1873147[1]	Nasal root breadth	0.00089	0.00889
12	16p13.3	ADCY9	rs8049367[6]	Nasal root breadth	0.02382	0.23820
13	17p13.1	NTN1	rs479177[6]	Columella inclination	0.03354	0.33540
14	17q22	NOG	rs227731[1,7]	Nasion position	0.02255	0.22550
15	20q12	MAFB	rs13041247[1,3,6]	Nasal root breadth	0.00002	0.00017

B) Kolmogorov-Smirnov test:

Combining all SNPs and all traits, a one-sided Kolmogorov-Smirnov test for deviation from the null hypothesis of no association was significant both for ordinal and for quantitative face traits. For both types of traits, a plot of the empirical cumulative distribution function (eCDF, in blue) of all individual P-values is shown next to the expected CDF (in purple) of P-values under the null of no association.

<u>Ordinal traits</u>

The one-sided Kolmogorov-Smirnov test had a P-value of 0.0011.

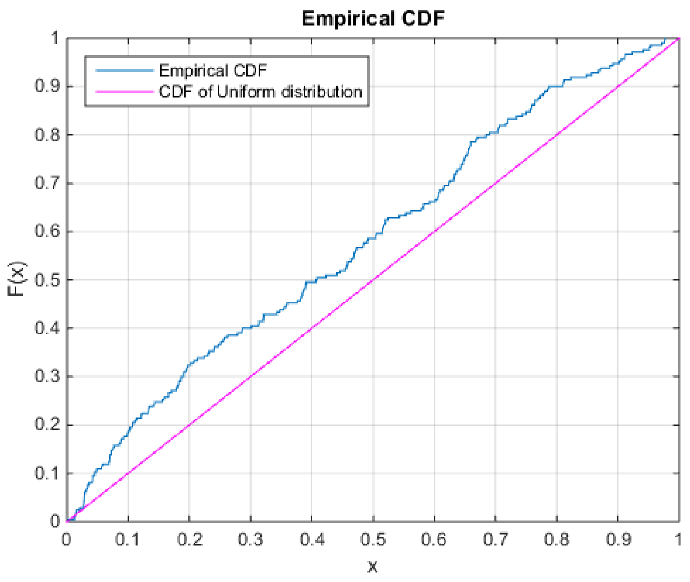

<u>Quantitative traits</u>

The one-sided Kolmogorov-Smirnov test had a P-value of 0.0046.

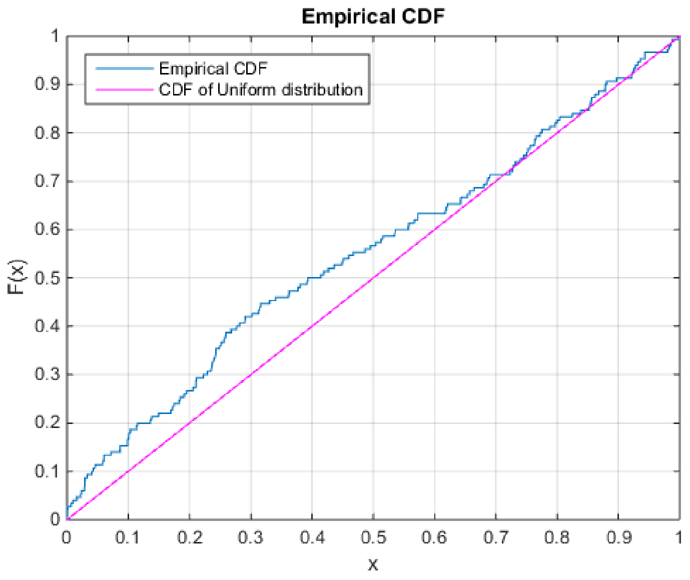

C) Polygenic risk score:

After regressing each SNP with a trait (controlling for covariates), regression coefficients from the 15 SNPs were combined into a composite Polygenic Risk Score (PRS). Each trait was then regressed against their corresponding PRS (controlling for covariates). The P-value from this regression is referred to below as the unadjusted P-value. Since the second regression uses a PRS score that combines the individual best-fit regression coefficients from the first regression, using this PRS score as a single variable in the same regression model results in overfitting. As a remedy, 2,000 simulations were performed for each trait, in which phenotypes were randomized across genotypes of each SNP (to maintain the same genotype distribution but under the null of no association with the phenotypes). These simulated genotypes and phenotypes were used to calculate PRSs and obtain an empirical distribution of the PRS test statistic. For each trait, the observed PRS test statistic was compared to this empirical distribution to obtain an 'adjusted' P-value.

Ordinal traits

Trait	Unadjusted P-value	Adjusted P-value
Forehead profile	9.26E-04	0.2310
Brow ridge protrusion	9.91E-07	0.0790
Cheekbone protrusion	8.98E-06	0.1985
Nasal root breadth	6.59E-08	0.0115
Nose bridge breadth	5.91E-06	0.1655
Nose wing breadth	2.13E-08	0.0070
Nose profile	3.44E-05	0.3105
Nose protrusion	2.15E-08	0.0110
Nose tip shape	3.65E-06	0.1195
Columella inclination	9.04E-03	0.9635
Upper lip thickness	4.16E-05	0.3485
Lower lip thickness	5.12E-05	0.3520
Chin shape	4.73E-06	0.1350
Chin protrusion	1.64E-06	0.0945

Quantitative traits

Trait	Unadjusted P-value	Adjusted P-value
Nasal root breadth	5.25E-12	<0.0005
Nose bridge breadth	1.15E-08	0.0060
Nose wing breadth	3.24E-07	0.0365
Nose protrusion	1.97E-04	0.5125
Nose tip shape	1.41E-03	0.8110
Columella inclination	1.88E-05	0.2420
Upper lip thickness	4.22E-05	0.3315
Lower lip thickness	3.16E-04	0.6015
Chin protrusion	3.10E-04	0.5925
Nasion Position	4.22E-05	0.3420

Supplementary Table 16: Effect of *Edar* genotype on mouse mandible length.

P values and regression coefficients (beta) for the linear regression between mandible length (as a proportion of head size) and *Edar* genotype are given below (controlling for age and sex). For each landmark on the mandible (5-7, Supplementary Figure 8) two types of distances were measured – direct and projected – with reference to landmark 10 (Supplementary Figures 8-9). These distances were divided by head size to convert into a proportion. All the *P* values were significant, suggesting that overall mandible length is affected by *Edar* genotype. The negative beta implies that mandible length becomes shorter with increased *Edar* function, consistent with the effect we see in humans.

Type	Distance	beta	*P* value
Projected	5–10	-0.01916	1.7E-04
Projected	6–10	-0.01543	2.6E-03
Projected	7–10	-0.01809	1.1E-04
Direct	5–10	-0.01680	1.3E-03
Direct	6–10	-0.01255	1.8E-02
Direct	7–10	-0.01136	1.3E-02

R regression code and output is shown below for the analysis of projected mandible distance 5–10 as an example. ‘mandible’ represents the projected distance, ‘head_length’ represents head size, ‘geno’ represents the coded *Edar* genotype, ‘age’ is a binary variable indicating whether age was 15 days or 14 days, and ‘sexF’ is a binary variable indicating whether sex was female or male.

```
> fit=lm(mandible/head_length~geno+age+sexF, data=data); summary(fit);

Coefficients:
             Estimate Std. Error t value Pr(>|t|)
(Intercept)  0.397934   0.008560  46.487  < 2e-16 ***
geno        -0.019155   0.004569  -4.192 0.000171 ***
age         -0.017585   0.008067  -2.180 0.035893 *
sexF        -0.001517   0.007997  -0.190 0.850657
---
Signif. codes:  0 ‘***’ 0.001 ‘**’ 0.01 ‘*’ 0.05 ‘.’ 0.1 ‘ ’ 1

Residual standard error: 0.02392 on 36 degrees of freedom
Multiple R-squared:  0.3579,     Adjusted R-squared:  0.3044
F-statistic: 6.689 on 3 and 36 DF,  p-value: 0.001052
```

Supplementary Table 17: Gene-gene interaction tests

Tests of interaction of pairwise SNPs were carried out for each trait by performing linear regressions of the trait value on covariates + index SNPs + pairwise interactions between index SNPs. In total 7 index SNPs were considered combining Tables 1 and 3. Other than the two cases for which there is biological evidence, no other interactions were found to be significant.

A) Interaction of *GLI3* with *RUNX2* and nose bridge breadth

Since Gli3 is known to interact with Runx2 in the regulation of mouse osteoblast differentiation, we tested for statistical interaction in our dataset. SNPs in *RUNX2* are significantly associated with categorical nose bridge breadth, so we ran a regression analysis for this trait including the index SNPs for *RUNX2* (rs1285029) and for *GLI3* (rs17640804) and an interaction term. The interaction term was adjusted beforehand to remove correlations with the main effects. The interaction term was highly significant, even though the GLI3 index SNP was not, providing support that the effect of *GLI3* on nose bridge breadth is through interaction only. The R regression output is provided below:

```
Call:
lm(formula = Nose_Bridge_Breadth ~ age + BMI + SEX + PC1 + PC2 +
    PC3 + PC4 + PC5 + rs1852985 + rs17640804 + interaction,
    data = big)

Coefficients:
                  Estimate Std. Error t value Pr(>|t|)
(Intercept)       1.841654   0.063317  29.086  < 2e-16 ***
age              -0.002469   0.001263  -1.955 0.050653 .
BMI               0.010840   0.002157   5.026 5.18e-07 ***
SEX              -0.241279   0.014267 -16.911  < 2e-16 ***
PC1               0.066260   0.578029   0.115 0.908742
PC2              -4.352393   0.547472  -7.950 2.25e-15 ***
PC3              -0.022513   0.522886  -0.043 0.965660
PC4               0.148741   0.542587   0.274 0.783992
PC5              -1.805611   0.533361  -3.385 0.000716 ***
rs1852985         0.066973   0.010853   6.171 7.27e-10 ***
rs17640804        0.016687   0.009929   1.681 0.092898 .
interaction       0.041660   0.014320   2.909 0.003638 **
---
Signif. codes:  0 ‘***’ 0.001 ‘**’ 0.01 ‘*’ 0.05 ‘.’ 0.1 ‘ ’ 1
```

A similar regression was performed for nose wing breadth (since that trait is associated with *GLI3*), but the interaction term was not significant in that case.

B) Interaction of *GLI3* with *PAX1* and nose wing breadth

Since experimental data show that Gli3 interacts with Pax1, we checked for a statistical interaction in our dataset for categorical nose wing breadth, which is associated with SNPs in both of these genes. The interaction effect between the *GLI3* and *PAX1* index SNPs (rs17640804 and rs927833 respectively) was also significant, in addition to their main effects. The R regression output is provided below:

```
Call:
lm(formula = Nose_Wing_Breadth ~ age + BMI + SEX + PC1 + PC2 +
    PC3 + PC4 + PC5 + rs927833 + rs17640804 + interaction,
    data = big)

Coefficients:
                 Estimate Std. Error t value Pr(>|t|)
(Intercept)      2.114152   0.067138  31.489  < 2e-16 ***
PC1             -4.761126   0.587589  -8.103 6.49e-16 ***
PC2             -8.774973   0.562619 -15.597  < 2e-16 ***
PC3              0.661149   0.556373   1.188  0.23476
PC4              0.895311   0.576295   1.554  0.12034
PC5             -1.666798   0.564107  -2.955  0.00314 **
SEX             -0.213757   0.015171 -14.090  < 2e-16 ***
age             -0.001481   0.001342  -1.104  0.26976
BMI              0.012685   0.002292   5.535 3.25e-08 ***
rs927833         0.077470   0.012282   6.308 3.04e-10 ***
rs17640804       0.064821   0.010621   6.103 1.11e-09 ***
interaction      0.048300   0.017203   2.808  0.00501 **
---
Signif. codes:  0 ‘***’ 0.001 ‘**’ 0.01 ‘*’ 0.05 ‘.’ 0.1 ‘ ’ 1
```

SUPPLEMENTARY REFERENCES

[1]. Quinto-Sánchez et al. (2015), Facial asymmetry and genetic ancestry in Latin American admixed populations. Am. J. Phys. Anthropol., 157: 58–70. doi: 10.1002/ajpa.22688.

[2]. An integrated encyclopedia of DNA elements in the human genome. Nature. 2012;489(7414):57-74.

[3]. Romanoski CE, Glass CK, Stunnenberg HG, Wilson L, Almouzni G. Epigenomics: Roadmap for regulation. Nature. 2015;518(7539):314-6.

[4]. Rojas A, Aguilar R, Henriquez B, Lian JB, Stein JL, Stein GS, et al. Epigenetic Control of the Bone-master Runx2 Gene during Osteoblast-lineage Commitment by the Histone Demethylase JARID1B/KDM5B. The Journal of biological chemistry. 2015;290(47):28329-42.

[5]. Drissi H, Luc Q, Shakoori R, Chuva De Sousa Lopes S, Choi JY, Terry A, et al. Transcriptional autoregulation of the bone related CBFA1/RUNX2 gene. Journal of cellular physiology. 2000;184(3):341-50.

[6]. Zambotti A, Makhluf H, Shen J, Ducy P. Characterization of an osteoblast-specific enhancer element in the CBFA1 gene. The Journal of biological chemistry. 2002;277(44):41497-506.

[7]. Lee MH, Kim YJ, Yoon WJ, Kim JI, Kim BG, Hwang YS, et al. Dlx5 specifically regulates Runx2 type II expression by binding to homeodomain-response elements in the Runx2 distal promoter. The Journal of biological chemistry. 2005;280(42):35579-87.

[8]. Tai PW, Wu H, Gordon JA, Whitfield TW, Barutcu AR, van Wijnen AJ, et al. Epigenetic landscape during osteoblastogenesis defines a differentiation-dependent Runx2 promoter region. Gene. 2014;550(1):1-9.

[9]. Napierala D, Garcia-Rojas X, Sam K, Wakui K, Chen C, Mendoza-Londono R, et al. Mutations and promoter SNPs in RUNX2, a transcriptional regulator of bone formation. Molecular genetics and metabolism. 2005;86(1-2):257-68.

[10]. Anderson et al. (2010), Data quality control in genetic case-control association studies. Nature Protocols 5, 1564–1573. doi:10.1038/nprot.2010.116.

[11]. Deng et al. (2001), Population admixture: detection by Hardy-Weinberg test and its quantitative effects on linkage-disequilibrium methods for localizing genes underlying complex traits. Genetics. 157:885–897.

[12]. Waples (2015), Testing for Hardy–Weinberg Proportions: Have We Lost the Plot? J Hered (2015) 106 (1): 1-19. doi: 10.1093/jhered/esu062.

[13]. Karlsson and Mork (2005), Deviation from Hardy–Weinberg equilibrium, and temporal instability in allele frequencies at microsatellite loci in a local population of Atlantic cod. ICES J. Mar. Sci. 62 (8): 1588-1596. doi: 10.1016/j.icesjms.2005.05.009.

[14]. Schraiber et al. (2012), Genomic tests of variation in inbreeding among individuals and among chromosomes. Genetics 192, 1477-82.

[15]. Fujimoto et al. (2008), A replication study confirmed the EDAR gene to be a major contributor to population differentiation regarding head hair thickness in Asia. Hum Genet 124, 179-85.

[16]. Sabeti et al. (2007), Genome-wide detection and characterization of positive selection in human populations. Nature 449, 913-8.

[17]. The Wellcome Trust Case Control Consortium (2007), Genome-wide association study of 14,000 cases of seven common diseases and 3,000 shared controls. Nature 447, 661–678.

[18]. Illumina Inc. (2008), GenomeStudioTM Genotyping Module v1.0 User Guide.

[19]. Guo et al. (2014), Illumina human exome genotyping array clustering and quality control. Nature Protocols 9, 2643–2662, doi:10.1038/nprot.2014.174.

[20]. Illumina Inc. (2004), Technical Note: Infinium® Genotyping Data Analysis.

[21]. Grove et al. (2013), Best Practices and Joint Calling of the HumanExome BeadChip: The CHARGE Consortium. PLoS ONE 8(7): e68095. doi:10.1371/journal.pone.0068095.

[22]. Ingersoll et al. (2011) Factors Associated with Success of Custom SNP Assays. The Genetic Resources Core Facility (GRCF), Johns Hopkins School of Medicine.

[23]. Muglia et al. (2010), Population-based linkage analysis of schizophrenia and bipolar case–control cohorts identifies a potential susceptibility locus on 19q13. Molecular Psychiatry 15, 319–325; doi:10.1038/mp.2008.100.

[24]. P. E. Shrout and Joseph L. Fleiss (1979). "Intraclass Correlations: Uses in Assessing Rater Reliability". Psychological Bulletin 86 (2): 420–428.

[25]. Dudoit, S. and Laan, M. J. v. d. Multiple testing procedures with applications to genomics. (Springer, 2008).

[26]. C. R. Rao (2008): Linear Statistical Inference and its Applications. 2nd Ed. John Wiley and Sons, Inc.

[27]. X Zhou and M Stephens (2014): Efficient multivariate linear mixed model algorithms for genome-wide association studies. Nature Methods 11, 407–409. doi:10.1038/nmeth.2848

[28]. M Stephens (2013): A Unified Framework for Association Analysis with Multiple Related Phenotypes. PLoS ONE 8(7): e65245. doi:10.1371/journal.pone.0065245

[29]. Ludwig KU, Mangold E, Herms S, Nowak S, Reutter H, Paul A, et al. Genome-wide meta-analyses of nonsyndromic cleft lip with or without cleft palate identify six new risk loci. Nature genetics. 2012;44(9):968-71.

[30]. Beaty TH, Taub MA, Scott AF, Murray JC, Marazita ML, Schwender H, et al. Confirming genes influencing risk to cleft lip with/without cleft palate in a case-parent trio study. Human genetics. 2013;132(7):771-81.

[31]. Beaty TH, Murray JC, Marazita ML, Munger RG, Ruczinski I, Hetmanski JB, et al. A genome-wide association study of cleft lip with and without cleft palate identifies risk variants near MAFB and ABCA4. Nature genetics. 2010;42(6):525-9.

[32]. Zucchero TM, Cooper ME, Maher BS, Daack-Hirsch S, Nepomuceno B, Ribeiro L, et al. Interferon Regulatory Factor 6 (IRF6) Gene Variants and the Risk of Isolated Cleft Lip or Palate. New England Journal of Medicine. 2004;351(8):769-80.

[33]. Rahimov F, Marazita ML, Visel A, Cooper ME, Hitchler MJ, Rubini M, et al. Disruption of an AP-2alpha binding site in an IRF6 enhancer is associated with cleft lip. Nature genetics. 2008;40(11):1341-7.

[34]. Sun Y, Huang Y, Yin A, Pan Y, Wang Y, Wang C, et al. Genome-wide association study identifies a new susceptibility locus for cleft lip with or without a cleft palate. Nature communications. 2015;6:6414.

[35]. Mangold E, Ludwig KU, Birnbaum S, Baluardo C, Ferrian M, Herms S, et al. Genome-wide association study identifies two susceptibility loci for nonsyndromic cleft lip with or without cleft palate. Nature genetics. 2010;42(1):24-6.

[36]. Birnbaum S, Ludwig KU, Reutter H, Herms S, Steffens M, Rubini M, et al. Key susceptibility locus for nonsyndromic cleft lip with or without cleft palate on chromosome 8q24. Nature genetics. 2009;41(4):473-7.

[37]. Grant SF, Wang K, Zhang H, Glaberson W, Annaiah K, Kim CE, et al. A genome-wide association study identifies a locus for nonsyndromic cleft lip with or without cleft palate on 8q24. The Journal of pediatrics. 2009;155(6):909-13.
